# The Intertidal North‐South Split: Oceanographic Features and Life History Shape the Phylogeography of Chiton *Acanthochitona rubrolineata*

**DOI:** 10.1111/eva.70095

**Published:** 2025-03-31

**Authors:** Shaobing Zong, Huijie Liu, Lingjing Xu, Dezhou Yang, Junlong Zhang

**Affiliations:** ^1^ Laboratory of Marine Organism Taxonomy and Phylogeny, Qingdao Key Laboratory of Marine Biodiversity and Conservation Institute of Oceanology, Chinese Academy of Sciences Qingdao China; ^2^ CAS Key Laboratory of Ocean Circulation and Waves Institute of Oceanology, Chinese Academy of Sciences Qingdao China; ^3^ University of Chinese Academy of Sciences Beijing China; ^4^ Marine Biological Museum Chinese Academy of Sciences Qingdao China

**Keywords:** genetic differentiation, ocean modeling, Polyplacophora, population structure, SNPs

## Abstract

The genetic structure and demographic history of marine organisms are shaped by a variety of factors including biological and ecological characteristics, ocean currents, and the palaeogeological effects of sea‐level fluctuations. Here we present a comprehensive method combining population genomics, laboratory experiments, and ocean modelling in 13 populations of the chiton *Acanthochitona rubrolineata* along the coast of China. Based on demographic and population genomic analyses, significant divergence was observed between the Northern and Southern population groups, which are separated by the Yangtze River Estuary. The numerical circulation model simulation showed that gene flow and population connectivity were strongly influenced by ocean currents and the larval dispersal ability of chiton *A. rubrolineata* . These data thus clearly revealed the presence of two separately evolving lineages in chiton— *A. rubrolineata* northern and *A. rubrolineata* southern. Our study highlights that a robust understanding of organisms in the intertidal zone requires a comprehensive consideration of factors that influence gene flow and genetic structure, including the life‐history traits, coastal currents, geographic isolation, and habitat suitability. The life history of marine organisms, together with local oceanographic features, could ultimately drive the population divergence and lead to speciation. These findings provide a guideline for future analyses of non‐model and potentially threatened species and will aid in the conservation of biodiversity.

## 1 Introduction

Understanding the factors that limit connectivity between marine populations is crucial for elucidating the evolutionary processes leading to divergence and speciation (Binks et al. 2011; Palumbi 1994), but also for their conservation and management (von der Heyden 2023). Accurately characterizing the genetic structure of marine species is a major challenge due to the large effective population sizes and high levels of gene flow facilitated by the lack of physical barriers (do Prado et al. 2018; Zhang et al. 2019; Zhao et al. 2018; Zong et al. 2021). Numerous factors, including historical disturbance, have been suggested to influence the population structure of marine organisms (Cheng et al. 2020; Stiller et al. 2021; Wilson and Eigenmann Veraguth 2010). Climate change during the Pleistocene may have caused geological and environmental changes in the marginal seas of the Northwest Pacific (Ni et al. 2020) that shaped the geographical distribution and establishment of refugial populations for a variety of coastal marine species during interglacial periods (Ni et al. 2020, 2014). In the Northwest Pacific, recent research has demonstrated the potential importance of the glacial–interglacial cycles as a driver of genetic diversity in marine species (Cheng et al. 2020; Chiu et al. 2023; Shen et al. 2011; Wang et al. 2022).

In addition to historical processes, contemporary factors are thought to influence the population genetic structure and distribution patterns of marine species (Li et al. 2017), including oceanographic barriers (Muñoz‐Ramírez et al. 2020), habitat discontinuities (Lee and Boulding 2009), variations in salinity and density of seawater (Yu et al. 2014), and species‐specific life histories (Ni et al. 2020). The Bohai Sea, Yellow Sea, and East China Sea are influenced by two distinct ocean current systems: a coastal current system and a warm current system. The former originates in the Bohai Sea and moves southwards along the Chinese coast, while the latter moves northwards and northeastwards (Guan 1994). The adults of many intertidal invertebrates are either sessile or relatively immobile, so they rely primarily on the mobility of larvae transported by oceanographic currents to connect among different populations (Cheng et al. 2020; Scheltema 1971). Although there is evidence to date suggesting that historical climate variability and contemporary factors can influence the genetic structure within the bivalve, gastropod, and cephalopod molluscan taxa (Abyzova et al. 2018; Guo et al. 2023; Williams et al. 2015), much less is known about how these factors shape phylogeographic patterns in species of other molluscan species, particularly polyplacophorans.

The chiton *Acanthochitona rubrolineata* is a polyplacophoran mollusc naturally distributed along the northwestern Pacific coast (Saito 2017; Zhang et al. 2015), and inhabiting hard substrates in intertidal zones. Although chitons are important members of the molluscan clade, our understanding of this group is spartan (Irisarri et al. 2020). Spawning of the chiton *A. rubrolineata* is typically annual, with the reproductive period extending from June to July (Yu et al. 2006). Sexual maturity is reached in less than two years, and the life span is typically four years or more (Hyman 1967). The planktonic nature of the larvae facilitates their dispersal via ocean currents, thereby promoting connectivity and gene flow between populations along the Chinese coast. There is currently a lack of direct data on the pelagic larval duration (PLD) of chiton *A. rubrolineata* . Evidence drawn from a number of other chiton species suggests that the trochophore stage typically has a total planktonic duration of less than one week (Ni et al. 2020; Pearse 1979). The chiton *A. rubrolineata* is therefore thought to have a limited dispersal capacity, which could potentially contribute to significant population structure between populations. Direct tracking of larval dispersal is difficult due to their small size. A commonly used indirect method is biophysical modelling, which estimates dispersal probabilities over a single generation and how the metapopulation is influenced by circulation patterns and dispersal barriers (Galindo et al. 2006). Genetic methods that estimate realized gene flow and hence dispersal are also commonly used (Blakeslee et al. 2021; Śmietanka et al. 2014). Consequently, a thorough genetic assessment is essential to clarify how these life‐history traits have interacted with ocean current systems to influence phylogeographic patterns in chiton *A. rubrolineata* and drive the speciation process.

To date, a large number of studies have been conducted to investigate the population structure and phylogeographic patterns of chiton *A. rubrolineata* based on mitochondrial and nuclear gene markers (Ni et al. 2020; Wang et al. 2019; Xu, Chu et al. 2020). Wang et al. (2019) analyzed the mitochondrial COI gene sequences for nine regional populations in the Bohai Rim and found no obvious phylogeographic structure. However, Xu, Chu et al. (2020) utilized concatenated COI and 16S rRNA gene sequences from eight sites along the Chinese coast, and the findings indicated a significant genetic differentiation between the northern populations and southern populations. Similarly, Ni et al. (2020) used COI and 16S rRNA mitochondrial gene fragments to show that the presumed limited dispersal ability of chiton *A. rubrolineata* , coupled with northeasterly currents, may have facilitated genetic differentiation among the three coastal regions of Korea, Japan, and China. While the mitochondrial genes provide valuable insights into the phylogeographic history of a species, they lack recombination and are typically inherited from only one parent, and have certain limitations in resolving population structure. Therefore, a genome‐wide sequence analysis is essential to provide a more comprehensive understanding of the population genetic relationships and demographic history of chiton *A. rubrolineata* .

The advent of next‐generation sequencing technology has made it possible to obtain thousands of molecular markers across the entire genome, which has greatly improved the accuracy and efficiency of population genetic studies conducted at the genomic level. The objective of this study is to investigate the fine‐scale genetic structure of chiton *A. rubrolineata* populations along the Chinese coast and to identify potential factors shaping population structure using thousands of SNPs obtained by reduced‐representation sequencing (RRS). The aims of this study were (1) to reveal the variations in genetic diversity and genetic structure among the populations, (2) to clarify the evolutionary history and gene flow, and (3) to elucidate the factors shaping phylogeographic patterns. The detailed population structure identified in this study may provide a new perspective on the population genetics of polyplacophorans along the Chinese coast, as well as the mechanisms that maintain their population structure. Furthermore, our results will serve as a basis for the future conservation of genetic diversity in marine organisms, which may be useful for monitoring and quantifying changes under warming coastal waters.

## 2 Methods

### 2.1 Sampling, DNA Extraction, and RAD‐Tag Sequencing

**FIGURE 1:**
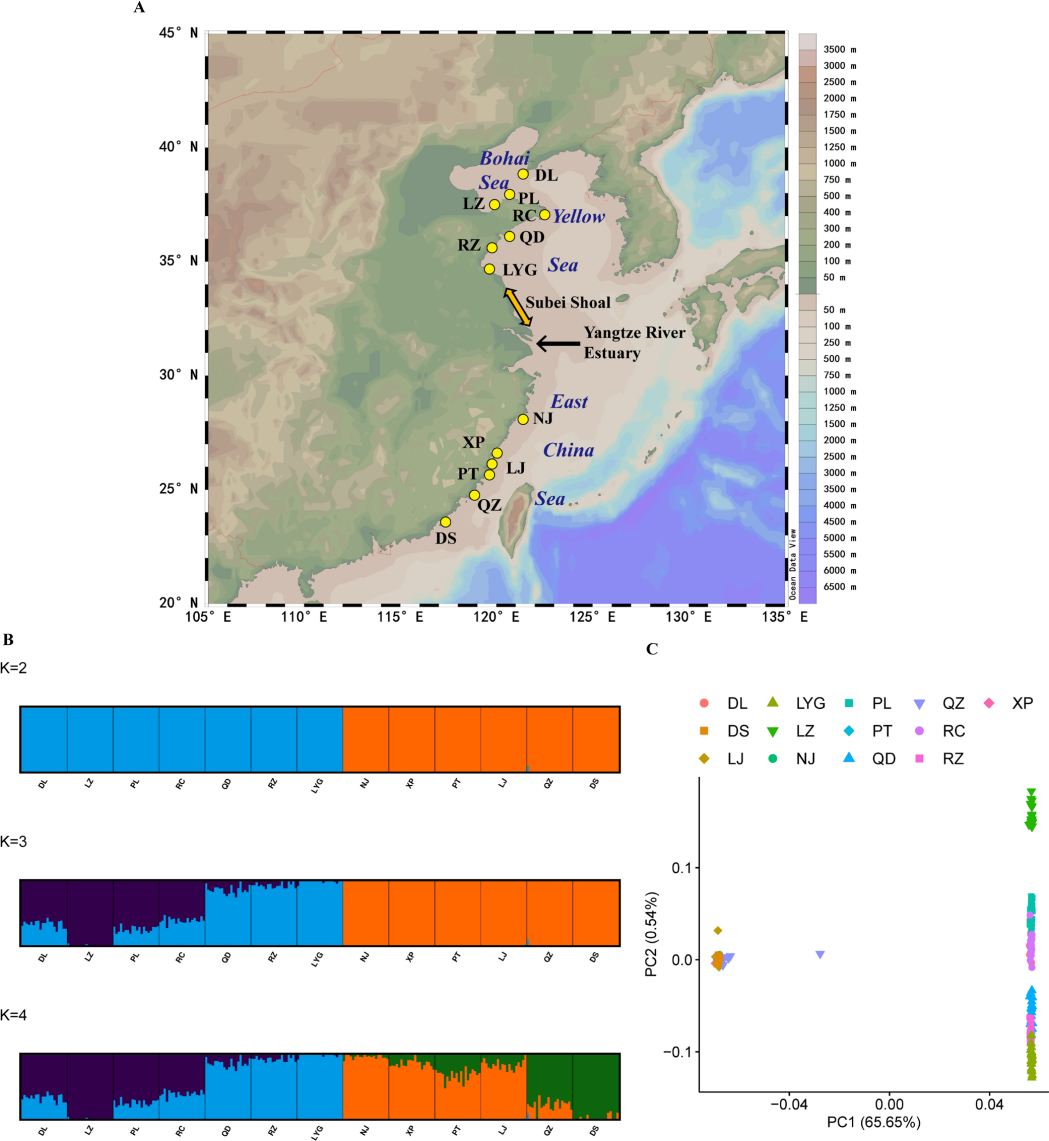
A map delineating the sampling locations for the thirteen populations, from which twenty individuals were collected at each location (A). Population genetic structure based on neutral loci for thirteen populations. (B) Admixture plot for *K* values from 2 to 4. (C) Principal component analysis (PCA) demonstrating the clustering of individuals. The first two PCs are displayed, with each individual represented by a unique symbol and color label corresponding to their respective populations of origin. DL, Dalian; DS, Dongshan; LJ, Lianjiang; LYG, Lianyungang; LZ, Laizhou; NJ, Nanji; PL, Penglai; PT, Pingtan; QD, Qingdao; QZ, Quanzhou; RC, Rongcheng; RZ, Rizhao; XP, Xiapu.

A total of 260 specimens of the chiton *A. rubrolineata* individuals were collected from 13 different localities covering Northern (Bohai Sea: DL, LZ and PL; Yellow Sea: RC, QD, RZ and LYG) and Southern (East China Sea: NJ, XP, LJ, PT, QZ and DS) populations along the Chinese coast (Figure 1 and Table S1). All populations were collected alive during 2019–2021 and identified based on morphological characteristics (Zhang et al. 2015). Muscle tissues from the foot were collected and preserved in 95% ethanol, then stored at −80°C until DNA extraction. Genomic DNA was extracted using the TIANamp Marine Animals DNA Kit (TIANGEN Biotech) following the manufacturer's protocol. RNase A treatment was used to remove RNA from the genomic DNA samples. The 2b‐RAD sequencing libraries were constructed using the *Bsa*XI restriction enzyme and sequenced on the Illumina NovaSeq 6000 system with 150 bp paired‐end reads at Qingdao OE Biotech Co. Ltd. (Qingdao, China), as described in detail by Wang et al. (2016).

### 2.2 RAD Data Process, SNP Genotyping, and Filtering

Raw sequence reads were first filtered using cutadapt v1.16 (Martin 2011) to remove read pairs with adaptors for quality control. Low quality reads (Phred score < 13) were removed using “process_radtags” in Stacks v1.48 (Catchen et al. 2011). PCR duplicated read pairs were removed using “clone_filter” in Stacks. A draft genome of the chiton *A. rubrolineata* (Zong et al. 2024) was used to map reads from each individual using BWA MEM v0.7.15‐r1140 (Li 2013) with default parameters. After mapping, SNPs were called using the Bayesian method implemented in the package SAMtools v1.9 and BCFtools v1.9 (Li et al. 2009). The command was as follows: samtools mpileup ‐Ou ‐b bam.lst ‐f genome_purged.fa ‐r ctg01:1–1,000,000 ‐q 1 ‐a DP, AD, SP, INFO/AD ‐p ‐‐ff UNMAP, SECONDARY, QCFAIL, DUP 2 > > ref_map//log/bcftools/bcftools.logs | bcftools call ‐M ‐Ob ‐f GQ ‐v ‐m ‐G popmap.txt ‐o ref_map//SNP/ctg01:1‐1,000,000.bcf 2 > > ref_map//log/bcftools/bcftools.logs. To obtain robust results in the subsequent analyses, SNPs of high quality were further filtered using the following criteria: (1) SNPs should be genotyped in at least 80% of individuals within each population; (2) SNP loci should be genotyped with a depth of coverage equal to or greater than 6; (3) an overall SNP quality score of 30 or greater and a genotyping score of 15 or greater; (4) SNPs with an observed heterozygosity greater than 0.5 for each population were also eliminated; (5) SNPs with a global minimum allele frequency (MAF) of less than 0.05 were discarded; (6) SNPs with a local MAF of greater than 0.2 in at least one population or the global MAF of 0.05 or greater across all the thirteen populations were retained. The filtered VCF file containing the SNPs was then converted to other required formats using PGDspider v2.1.1.5 (Lischer and Excoffier 2012).

For population genetic and demographic analyses assuming a set of random and unlinked markers, only one SNP within a 10 Kb region was retained to eliminate potential linkage disequilibrium (LD) using VCFtools v0.1.16 (Danecek et al. 2011). Unless otherwise stated, this random and unlinked data set was used as the primary data set for subsequent population genetic analyses (ADMIXTURE, principal component analysis (PCA), discriminant analysis of principal components (DAPC), neighbor‐joining tree, and *F*_ST_) and demographic analysis.

### 2.3 Summary Statistics and Population Genetic Structure

Genetic diversity statistics, including observed (*H*_O_) and expected (*H*_E_) heterozygosity of SNPs within all 13 sampling locations, were calculated using *populations* in Stacks v1.48 (Catchen et al. 2011). The fixation index (*F*_ST_) between each pair of populations was estimated using Arlequin v3.5.2.2 (Excoffier and Lischer 2010), and the significance was examined using an exact test with 10,000 permutations. Significance was then adjusted using the FDR‐BY method.

The population structure of 260 chiton *A. rubrolineata* individuals from 13 locations was assessed and visualized by four methods. First, population structure was inferred by using the model‐based program ADMIXTURE v1.3.0 (Alexander et al. 2009). The number of clusters (K) from 1 to 14 was tested with 10 replicates, and the optimal number of K was determined using StructureSelector (Li and Liu 2018) based on both cross‐validation and Puechmaille methods. Second, PCA was performed using GCTA software (Yang, Lee et al. 2011), and the scatterplot of the first and second components was generated using R (https://www.r‐project.org/). Third, DAPC (Jombart et al. 2010) implemented in the R package adegenet v.2.1.1 (Jombart 2008) was used to identify clusters of genetically related individuals. The optimal number of retained principal components for the DAPC was determined using the “optim.a.score” function. Finally, pairwise distance (*p*‐dist) matrices between each pair of individuals were calculated using VCF2DIS v.1.42 (https://github.com/BGI‐shenzhen/VCF2Dis), and the Neighbor‐Joining tree was constructed by FastME (Lefort et al. 2015) and visualized using the R package “ape” (Paradis and Schliep 2018).

In order to investigate the genomic heterogeneity of differentiation, we identified the most differentiated regions of the genome between the two major lineages and between sublineages. Using the “‐‐weir‐fst‐pop” function of the VCFtools v0.1.16 (Danecek et al. 2011), we computed the *F*_ST_ statistic for each locus between the Northern and Southern group, the Bohai Sea and Yellow Sea clusters, and Northern and Southern East China Sea clusters. These pairwise *F*_ST_ values were used to generate the scatter plot and *F*_ST_—*F*_ST_ coplots, following the approach of Lapègue et al. (2023). Additionally, we computed the *F*_ST_ values between LZ and LYG populations, and NJ and DS populations, where genetic differences between the two population pairs were particularly pronounced. To find out whether there were parallel patterns of differentiation, we computed Patterson et al.'s f4 statistics (Patterson et al. 2012) using the formula: f4 = (p_BS ‐p_YS) (p_NESC ‐p_SESC), where p_BS, p_YS, p_NESC, and p_SESC represent the allele frequency in the Bohai Sea, Yellow Sea, Northern, and Southern East China Sea genetic clusters, respectively. By definition, f4 yields positive values when differentiation is parallel and negative values when differentiation is antiparallel.

### 2.4 Estimating Historical Relationships

TreeMix was used to infer the evolutionary relationships between sampling sites (including population splits and migration events) (Pickrell and Pritchard 2012). To avoid bias associated with missing data, the neutral dataset was filtered for this analysis. The population graph was run allowing between 0 and 14 migration events, and the likelihood was monitored to determine when it plateaued for estimating the number of migration events.

To infer the historical demographic processes among different populations of chiton *A. rubrolineata* , a demographic analysis was performed using the Approximate Bayesian Computation method as implemented in DIYABC v2.1.0 (Cornuet et al. 2014). DIYABC uses a coalescent framework to simulate complex evolutionary scenarios without the need to estimate the underlying likelihood function. For the demographic simulations, we utilized 1000 SNPs randomly selected from the unlinked dataset three times. We evaluated four distinct scenarios to infer the historical demographic processes (see Results section for details). DIYABC provides mean values for each event in generations, which we converted into dates using a generation time of two years for chiton *A. rubrolineata* (Cherns 1999). Four historical scenarios tested are shown in Figure S1 and further parameter details of each model are described in Table S2.

Bayesian skyline plot (BSP) analysis was performed on a randomly selected dataset of 1000 SNPs using BEAST v2.7.6 (Bouckaert et al. 2014) to infer the demographic history of effective population size for chiton *A. rubrolineata* populations. These analyses were performed separately with genetically differentiated geographic groups of populations identified in this study as follows: Northern groups (seven populations) and Southern groups (six populations). We chose the GTR model with Empirical Frequencies. For the clock model, we used a Strict Clock with a Mean clock rate of 1.0e‐7. The dimension of bPopSizes and bGroupSizes was set to 4 in the Initialization panel. We used the Coalescent Bayesian Skyline model as priors with the gammaShape parameter set to Exponential. We ran an MCMC chain length of 100 million iterations per group, sampling every 1000 generations to estimate the effective population size change. We discarded the first 10% of each run (burn‐in period) and averaged the runs to obtain reasonably high effective sample sizes (ESS ≥ 200). Results were checked and visualized using Tracer 1.7.1 (available from http://beast.bio.ed.ac.uk/Tracer).

In order to complement the demographic reconstruction analysis, GADMA was implemented to assess the directionality of gene flow (Noskova et al. 2020). GADMA uses the joint site frequency spectrum (JSFS) to infer the demographic history of multiple populations (Noskova et al. 2020). GADMA2 (Noskova et al. 2022) estimates a JSFS for each population and evaluates the parameter space to find the most likely parameter values through comparisons to the observed SFS from the composite‐likelihood scores and Akaike Information Criterion (AIC) statistics. Our data revealed two geographically distinct genetic clusters: the Northern group and the Southern group. We inferred the demographic histories of each of these regions through two replicate site comparisons. Specifically, we chose to compare LYG (north) vs. NJ (south) due to their close geographic proximity, and DL (north) vs. DS (south) to determine whether results remained consistent over a broader spatial extent.

Joint site frequency spectrums for each pair of sites were generated from VCF files using the easy SFS python script (https://github.com/isaacovercast/easySFS). We used one SNP per locus, assuming loci were unlinked, with an effective sequence length (*L*) of 1,302,336 bp. Given the absence of precise mutation rate (*μ*) data for this species, we used 1 × 10^−9^ substitutions per site per generation (Pogson and Zouros 1994; Zhao et al. 2014), assuming a generation time of 2 years (Cherns 1999). This mutation rate is a widely used value, yet may significantly deviate from reality (Duda Jr. 2021). Running parameters were Theta (*θ*): 0.0052, which was calculated as 4*μ*L, as in Gutenkunst et al. (2009).

*Moments* (Jouganous et al. 2017) was selected to simulate allele frequency spectra from demographic models due to its robustness and computational efficiency (Noskova et al. 2020). The initial model structure was set to one time interval before the population split and one after (1,1). The final model structure was also set to (1,1), with remaining parameters as default. We imposed no constraints on divergence times. The genetic algorithm (GA) parameters were left as default, as recommended by the software developers and validated across various datasets and demographic scenarios (Noskova et al. 2020). The BFGS_log method was used for local model optimization after each GA run. Each demographic model was run with 50 repeats, and the best run was selected based on Log‐likelihood. Although the demographic modeling provides time estimates for events, potential biases in using variable sites and the lack of an accurate *θ* estimate (i.e., the expected number of mutations per site per generation) may introduce errors in the absolute timing of demographic events (Noskova et al. 2020). In addition to simple models, we also tested more complex demographic scenarios, including one time interval before the split and two after (1,2). Together, these models covered a range of demographic scenarios, including the four most common inferred from genomic analyses: strict isolation (SI), ancient migration (AM), isolation with migration (IM), and secondary contact (SC).

### 2.5 Estimating Optimal Pelagic Duration of *A. rubrolineata* and Larval Dispersal Modelling

Adult specimens of the chiton *A. rubrolineata* (Lischke 1873) were obtained from intertidal rocks in Qingdao, China. Upon arrival at the laboratory, the specimens were placed in a 100‐mL plastic beaker filled with fresh seawater. During their reproductive season from June to August, a subset of these individuals began spawning within approximately three hours post‐transfer (Xia et al. 2023). Sperm was introduced into the suspended oocytes for artificial fertilization, and the resulting zygotes were cultured in filtered seawater (FSW) at a temperature of 25°C in an incubator. Developmental stages were expressed as hours post fertilization (hpf).

To test the optimal pelagic duration of chiton *A. rubrolineata* larvae, we carried out a laboratory experiment at our experimental facilities (hereafter referred to as the “settlement experiment”). The trochophore larvae hatched after 8.5 hpf (Xia et al. 2023). The larvae usually settle down when they encounter a solid object and can no longer swim. In our settlement trials without suitable conditions for settlement, the larvae will be pelagic and until dead with no food supply. According to the climatic conditions of Qingdao from June to August, the temperatures of FSW were set and maintained at 17°C, 20°C, 23°C, 25°C, and 28°C controlled by the thermostatic incubators. Healthy larvae were harvested from the upper half of the water column at approximately 10 hpf. Approximately 200 larvae per treatment were placed into a 10 cm culture plate. The five culture plates were randomly assigned to the incubators. Change the FSW with pre‐set temperatures every day. Larvae were observed daily for survival (alive or dead) using a binocular microscope.

A larval dispersal model was developed specifically for oceanic conditions in the Northwest Pacific. This model involved the “release” of virtual particles, representing *A. rubrolineata* larvae, from sampling locations along the Chinese coast. These particles were then transported by simulated ocean currents throughout their designated pelagic larval phase. The diffusion and advection processes of this biological tracer within the ocean were simulated using a regional ocean general circulation model. This model was originally developed by Yang et al. (2012) and is based on the Regional Ocean Model System (ROMS) (Shchepetkin and Mcwilliams 2005). Our model domain extended from 21° N to 41° N and from 116° E to 126° E. The model grid featured a horizontal resolution of 1/12° × 1/12° and incorporated 26 non‐linear terrain‐following layers vertically (Xu, Yang et al. 2020). Vertical mixing was represented using the K‐Profile Parameterization (KPP) scheme (Large et al. 1994), with background vertical viscosity and diffusivity set to 10^−5^ m^2^ s^−1^ (Pereira et al. 2002). Both initial and open boundary fields were sourced from a larger domain climatology model, as described in Yang, Yin et al. (2011). The monthly mean wind stress for the climatology was computed from data obtained from the Comprehensive Ocean–Atmosphere Data Set (COADS) (Diaz et al. 2002).

To delineate the density and trajectory of simulated larval dispersal, passive tracers with a concentration of 100 million floats/m^3^ were deployed at a depth of 2.5 m at sampling locations along the Chinese coastline. The movement of the passive tracers is governed by the advection–diffusion equation: $\frac{\partial P_{t}}{\partial t}+\vec{u}∙\nabla P_{t}=\frac{\partial }{\partial z}\left(K_{P_{t}}\frac{\partial P_{t}}{\partial z}\right)+D_{P_{t}}+S_{P_{t}}$, where $P_{t}$ denotes the concentration of the passive tracer, $\vec{u}$ signifies the current velocity derived from the model domain, $K_{P_{t}}$ stands for vertical diffusivity, $D_{P_{t}}$ symbolizes the horizontal diffusion term, and $S_{P_{t}}$ represents source terms. Both the horizontal and vertical mixing schemes used for the tracer are consistent with those used in the momentum equations. By introducing the passive tracer at specific sampling locations, it becomes possible to track the diffusion path of the larvae and determine their density through the tracer concentration. The experiments were designed to simulate optimal PLD based on the results of the settlement tests, utilizing daily‐mean tracer concentration fields for subsequent analysis.

## 3 Results

### 3.1 Genetic Variation

Illumina sequencing generated ~2.7 billion reads, of which 2.4 billion (average of 9.3 million reads per sample) remained after filtering out low quality reads. A total of 3,088,866 RAD tags passed the filters, surveying a total of 83,399,948 bases (~8.1% of the 1034 Mb unpublished chiton *A. rubrolineata* genome assembly). A total of 1,209,435 single nucleotide polymorphisms (SNPs) were identified by comparison with the reference genome. Following a stringent filtering procedure, 18,886 SNPs were retained for subsequent analysis.

### 3.2 Population Structure

The observed and expected heterozygosity (*H*_O_ and *H*_E_) were comparable among the Northern (DL, LZ, PL, RC, QD, RZ, and LYG) populations and the Southern (NJ, XP, PT, LJ, QZ, and DS) *A. rubrolineata* populations, respectively. However, the Southern populations exhibited higher levels of heterozygosity than their Northern counterparts (Table 1). *H*_O_ ranged from 0.095 ± 0.001 in the LYG population to 0.145 ± 0.002 in the LJ population, while *H*_E_ varied from 0.093 ± 0.001 in the LYG population to 0.133 ± 0.001 in the XP and LJ populations.

**TABLE 1:** Summary of genetic diversity statistics for thirteen distinct populations of chiton *Acanthochitona rubrolineata*.

Population	Code	*H*_O_	*H*_E_	*π*	*F*_IS_
Dalian	DL	0.096 ± 0.001	0.095 ± 0.001	0.097 ± 0.001	0.004 ± 0.008
Laizhou	LZ	0.096 ± 0.001	0.094 ± 0.001	0.096 ± 0.001	0.002 ± 0.009
Penglai	PL	0.097 ± 0.001	0.096 ± 0.001	0.099 ± 0.001	0.007 ± 0.005
Rongcheng	RC	0.097 ± 0.001	0.095 ± 0.001	0.098 ± 0.001	0.004 ± 0.011
Qingdao	QD	0.098 ± 0.001	0.096 ± 0.001	0.099 ± 0.001	0.002 ± 0.010
Rizhao	RZ	0.097 ± 0.001	0.096 ± 0.001	0.099 ± 0.001	0.005 ± 0.008
Lianyungang	LYG	0.095 ± 0.001	0.093 ± 0.001	0.096 ± 0.001	0.003 ± 0.008
Nanji	NJ	0.137 ± 0.001	0.130 ± 0.001	0.133 ± 0.001	−0.006 ± 0.013
Xiapu	XP	0.144 ± 0.002	0.133 ± 0.001	0.137 ± 0.001	−0.015 ± 0.007
Pingtan	PT	0.136 ± 0.001	0.131 ± 0.001	0.135 ± 0.001	0.001 ± 0.010
Lianjiang	LJ	0.145 ± 0.002	0.133 ± 0.001	0.137 ± 0.001	−0.017 ± 0.007
Quanzhou	QZ	0.135 ± 0.001	0.131 ± 0.001	0.135 ± 0.001	0.004 ± 0.010
Dongshan	DS	0.133 ± 0.001	0.130 ± 0.001	0.133 ± 0.001	0.005 ± 0.008

*Note:* Summary statistics included the mean of observed (*H*_O_), expected (*H*_E_) heterozygosity, nucleotide diversity (*π*) and inbreeding coefficient (*F*_IS_).

The genome‐wide fixation indices (*F*_ST_) among the Northern populations were predominantly low yet significant, with an average of 0.04. The exception was observed in the comparison between QD and RZ populations (Table 2). The *F*_ST_ among the Southern populations was similar to those among the Northern populations, with a lower mean value of 0.02, except for the comparison between NJ and XP populations. The *F*_ST_ between Northern and Southern populations of *A. rubrolineata* was remarkably high and statistically significant, averaging at 0.80.

**TABLE 2:** Population pairwise *F*_ST_ used by neutral loci.

	DL	LZ	PL	RC	QD	RZ	LYG	NJ	XP	PT	LJ	QZ	DS
DL	0												
LZ	**0.048**	0											
PL	**0.020**	**0.035**	0										
RC	**0.019**	**0.043**	**0.011**	0									
QD	**0.032**	**0.054**	**0.029**	**0.023**	0								
RZ	**0.037**	**0.058**	**0.033**	**0.026**	0.004	0							
LYG	**0.050**	**0.072**	**0.048**	**0.045**	**0.033**	**0.031**	0						
NJ	**0.797**	**0.799**	**0.796**	**0.796**	**0.796**	**0.796**	**0.799**	0					
XP	**0.794**	**0.796**	**0.793**	**0.794**	**0.793**	**0.794**	**0.796**	0.004	0				
PT	**0.796**	**0.798**	**0.795**	**0.795**	**0.795**	**0.795**	**0.798**	**0.009**	**0.005**	0			
LJ	**0.795**	**0.797**	**0.794**	**0.794**	**0.794**	**0.794**	**0.797**	**0.005**	**0.003**	**0.003**	0		
QZ	**0.796**	**0.798**	**0.795**	**0.795**	**0.795**	**0.795**	**0.798**	**0.024**	**0.017**	**0.009**	**0.015**	0	
DS	**0.797**	**0.799**	**0.796**	**0.796**	**0.796**	**0.796**	**0.799**	**0.036**	**0.030**	**0.020**	**0.027**	**0.008**	0

*Note:* Significant values after FDR‐BY correction (*p* < 0.01012) are highlighted in bold.

The ADMIXTURE results suggested that genetic variation was predominantly segregated by geographic region, with the strongest support for *K* = 2 (Figure 1B and Figure S1). With *K* = 2, the Northern populations coalesced into one distinct cluster, while the Southern populations formed another. With *K* = 4, among the Northern group, DL, LZ, PL, and RC populations formed one cluster, and QD, RZ, and LYG populations formed the second cluster. Among the Southern group, NJ, XP, PT, and LJ populations formed the third cluster, while QZ and DS populations formed the fourth cluster. The PCA also recovered two groupings (Figure 1C), i.e., the Northern group and the Southern group, where the first and second eigenvectors had cumulative variability values of 66.19%. For the Northern group, three subtle subdivisions can be found, while for the Southern group, no subdivision showed high gene flow between populations. DAPC plots confirmed the results obtained with ADMIXTURE for the main North–South subdivision, but also suggested a further clustering within the Northern group (Figure S2B). The Neighbor‐Joining tree of *A. rubrolineata* populations reveals two primary clusters: the Northern group coalesces into one cluster, while the Southern group populations form the other (Figure S2C). Among the Northern group, individuals from each population clustered independently. Notably, for the Southern group, only the individuals from NJ, QZ, and DS populations clustered independently. The remaining individuals from XP, PT, and LJ populations demonstrated irregular clustering patterns with each other, indicating a high degree of gene flow among these populations. All analyses strongly supported the subdivision of chiton *A. rubrolineata* along the Chinese coast into two distinct groups. Within the Northern group, two distinct subgroups were clearly separated by geographic region, specifically the Bohai Sea and the Yellow Sea. Conversely, the genetic variation in the Southern group was remarkably low.

**FIGURE 2:**
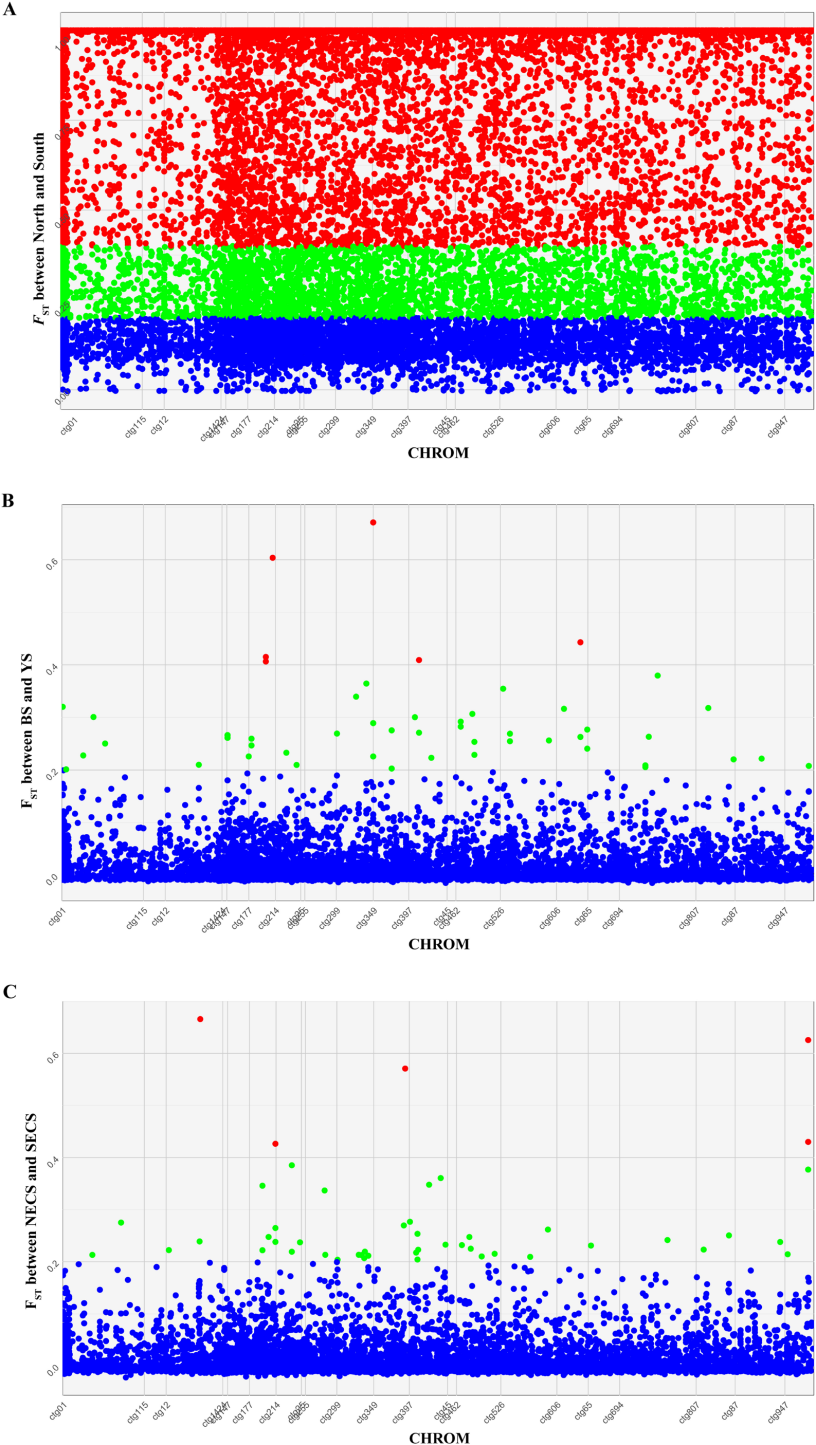
Scatter plot of *F*_ST_ estimates against genome position (contigs) calculated between the Northern and Southern group (A), the Bohai Sea (BS) and Yellow Sea (YS) clusters (B), and Northern (NESC) and Southern East China Sea (SESC) clusters (C). Blue dots represent *F*_ST_ values less than 0.2, green dots represent *F*_ST_ values greater than 0.2 but less than 0.4, and red dots represent *F*_ST_ values greater than 0.4.

The scatter plots (Figure 2A) revealed highly differentiated genetic regions between the Northern and Southern populations, distributed across the entire genome based on our unpublished genome assembly results. Within each population, however, genetic differentiation was lower: six and five SNP loci exhibited relatively high genetic differentiation within the Northern and Southern populations, respectively, located on different contigs (Figure 2B,C). The *F*_ST_ values for most loci were less than 0.4, with some were even below 0.2, confirming low genetic differentiation within the Northern and Southern populations but significantly higher between the two groups. Patterson et al.'s f4 statistics showed that 93.7% of f4 data was zero, with only 3.1% positive and 3.2% negative, indicating predominantly antiparallel differentiation. *F*_ST_ – *F*_ST_ coplots for the 18,886 SNP loci (Figure S3) also did not show a tendency for genetic parallelism. Notably, the most differentiated loci between the Bohai Sea and Yellow Sea clusters exhibited very low *F*_ST_ values (being 0) between Northern and Southern East China Sea clusters. This pattern was consistent between LZ and LYG populations, as well as between NJ and DS populations. However, a few SNP loci did not follow this antiparallel differentiation pattern and instead highlighted local differentiation of one genetic cluster against the other three.

### 3.3 Demographic History

**FIGURE 3:**
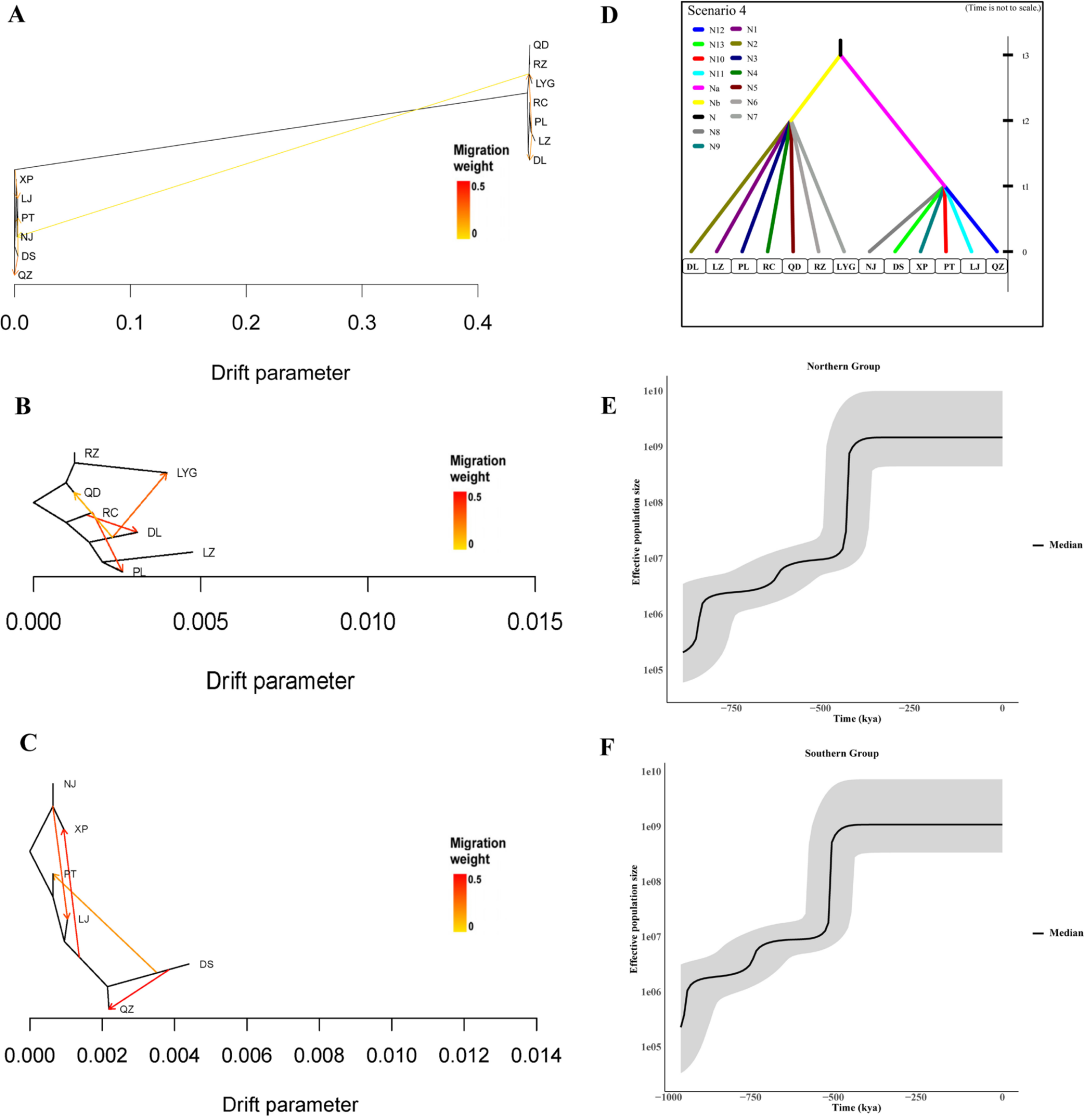
Maximum likelihood tree using the thirteen populations (A), Northern group (B) and Southern group (C) populations. The arrows in each chart denote 6, 4, and 4 migration events from the origin to the recipient population. Migration arrows are assigned a color proportional to their weight. (D) Scenario 4 simulated in DIYABC to evaluate the population demographic history of chiton *A. rubrolineata* . Bayesian skyline plots depicting the historical demography of Northern populations (E) and Southern populations (F), respectively. The mean value is denoted by a solid line, while the 95% highest posterior density is indicated by a dotted line. DL, Dalian; DS, Dongshan; LJ, Lianjiang; LYG, Lianyungang; LZ, Laizhou; NJ, Nanji; PL, Penglai; PT, Pingtan; QD, Qingdao; QZ, Quanzhou; RC, Rongcheng; RZ, Rizhao; XP, Xiapu. In the scenario, t# represents the time‐scale in terms of the number of generations, and N# represents the effective population size of a population. N: Effective population size (*N*_e_) of an ancestral population, N1: *N*_e_ of Dalian, N2: *N*_e_ of Laizhou, N3: *N*_e_ of Penglai, N4: *N*_e_ of Rongcheng, N5: *N*_e_ of Qingdao, N6: *N*_e_ of Rizhao, N7: *N*_e_ of Lianyungang, N8: *N*_e_ of Nanji, N9: *N*_e_ of Xiapu, N10: *N*_e_ of Pingtan, N11: *N*_e_ of Lianjiang, N12: *N*_e_ of Quanzhou, N13: *N*_e_ of Dongshan.

The TreeMix analysis clustered two subdivisions into Northern and Southern groups (Figure 3A), essentially corroborating the results of previous analyses. The TreeMix analysis revealed six migration events (*m* = 6) for all 13 populations. Stronger connections were observed between the Bohai Sea and Yellow Sea, while the connections between populations from the Northern and Southern groups were relatively weak. The results of the tree model agreed with the population state (Figure S2C) and a *K* = 2 in the ADMIXTURE stacking map (Figure 1B). Gene flow between the Northern and Southern populations was minimal, as evidenced by an almost nonexistent migration event. In the Northern clade, the TreeMix analysis identified four migration events (*m* = 4, Figure 3B). Among them, there were stronger connections among the Bohai Sea (RC to PL, and RC to DL population migration events). The connection between the Bohai Sea and the Yellow Sea (DL to LYG population migration events) was also strong. A weaker migration was detected from the DL population to the QD population. The RC population had the greatest convergence among all Northern populations. For the Southern clade, the TreeMix analysis revealed four migration events (*m* = 4, Figure 3C). Among them, there were strong connections among the East China Sea (DS to QZ, DS to PT, DS to XP, and NJ to LJ population migration events). The DS population had the greatest convergence among all Southern populations.

The scenarios were constructed using the results of population structure analyses, the geographic history of the Yangtze River, and insights into oceanic currents in DIYABC analyses. To reduce computation, two populations from each group (Northern group: DL and LYG populations, Southern group: NJ and DS populations) were chosen to estimate posterior probabilities of demographic history scenarios. In particular, four different scenarios were assessed to estimate the divergence time between the Northern and Southern populations of *A. rubrolineata* (Figure S4). The demographic processes among the intra‐group were trivial and ignored. The prior distributions of the demographic parameters for the simulations are presented in Table S2. For simplicity, each scenario was simulated with 1e6 runs (total number of simulations = 3 × 4 × 1e6). The posterior probability of scenario 4 was significantly higher than the other scenarios (posterior probability = 0.996, 95% CI: 0.972–1.000; Table S3). The common ancestral population of the Northern and Southern groups diverged at time t3 from an ancestral population of size N. Subsequently, the Northern group populations were derived simultaneously from the common ancestral population at time t2. The populations of the Southern group were simultaneously derived from the common ancestral population at time t1. Finally, all the thirteen populations were used in scenario 4 to estimate the divergence time between the Northern and Southern groups (Figure 3D). The common ancestral population of the Northern and Southern populations diverged at 578,000 (95% CI: 445,000–720,000) generations ago. Using a generation time of two years, this corresponds to a divergence time of approximately 1,156,000 (95% CI: 890,000–1,440,000; Table S4) years ago.

The BSP results showed strong signals of sudden increases in population size. The shape of the BSPs was quite similar across the two geographic groups, showing nearly synchronous population expansions. The population size of the Northern and Southern groups experienced a significant increase at 0.86 Mya (Northern group) and 0.96 Mya (Southern group) after their initial split (Figure 3E,F). Both lineages then underwent a second, more recent population expansion, beginning at 0.44 Mya (Northern group) and 0.52 Mya (Southern group), after an extended period of population stagnation. Furthermore, the BSPs generally exhibited very similar plateau phases with no change in population size after the second expansion until the present.

We identified the regional genetic structure between the Northern and Southern *A. rubrolineata* and inferred the demographic histories of two population pairs (i.e., replicates): LYG (north) vs. NJ (south) and DL (north) vs. DS (south). The list of models and parameter space for each dataset are shown in Tables S5 and S6. For simple model replicates, the best‐ranked model identified population divergence via isolation with asymmetric or no migrations (Tables S5, Figures S5 and S6). The estimated timing of divergence from the ancestral population for LYG vs. NJ was 59 Kya, and for DL vs. DS was 415 Kya (Figure S5). Estimated migration rates were higher from the south to the north for the DL vs. DS comparison. Migration rates from DS (south) to DL (north) were 1.97 × 10^−7^, whereas migration in the inverse direction was 9.71 × 10^−7^. For the LYG versus NJ model, the effective size of the ancestral population was larger in the north than in the south (73,523 versus 42,515, respectively). For the DL versus DS model, the effective size of the ancestral population was one order of magnitude smaller in the north than in the south (45,437 versus 238,636, respectively). Population contractions after divergence were observed in both comparisons. In the south, contractions were linear with contemporary effective population sizes of 3,883 and 2,983 at NJ and DS, respectively. In the north, contemporary effective population sizes were at 3288 and 10,110 for LYG and DL, respectively. Population contractions at LYG were exponential, whereas contractions at DL were linear (Figure S5).

**FIGURE 4:**
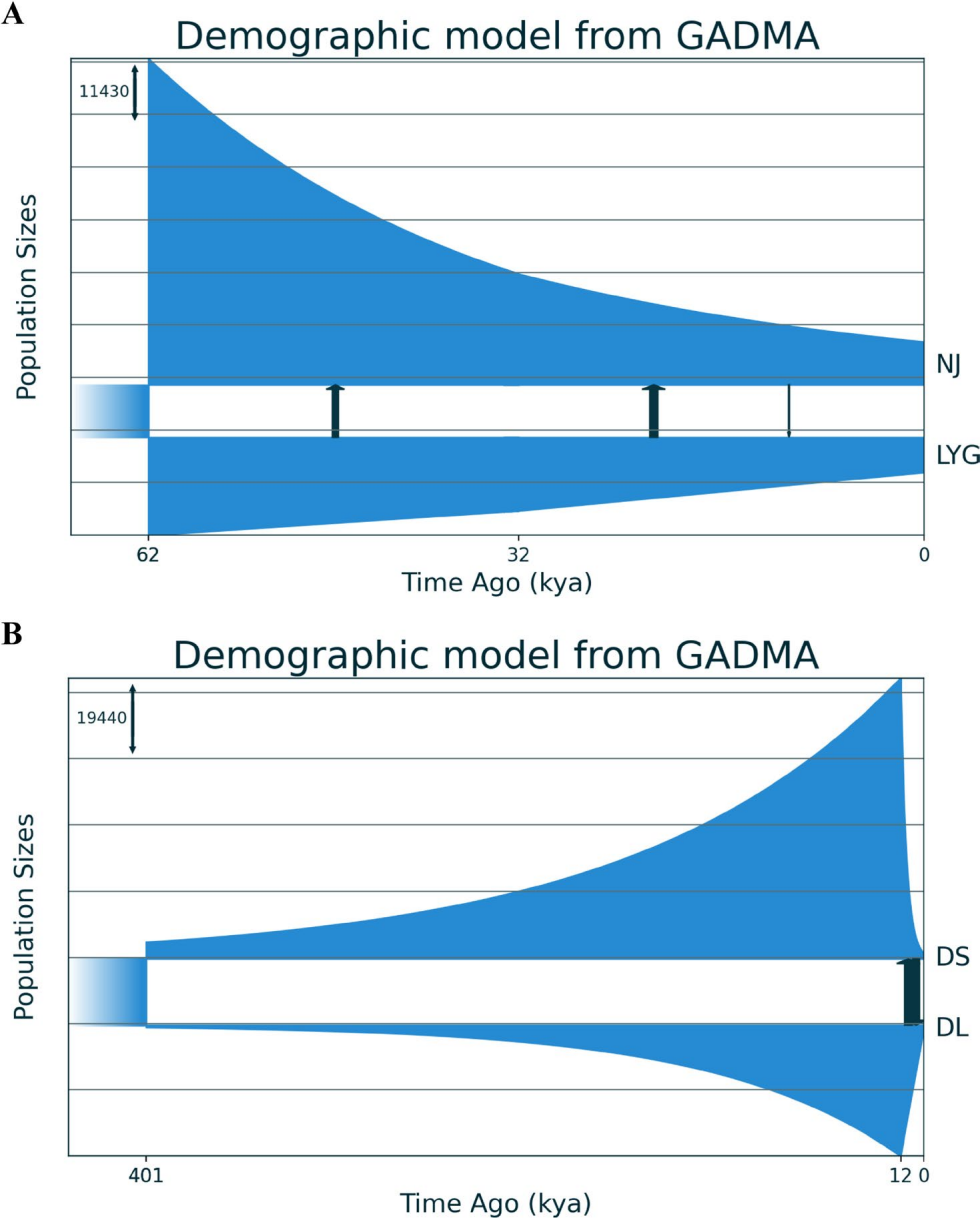
Best‐ranked “complex” demographic models describing divergence and changes in population size between (A) LYG (north) and NJ (South), and (B) DL (north) and DS (South). Demographic models were constructed using the diffusion approximation method (*moments*; Jouganous et al. 2017) implemented in the program GADMA2 (Noskova et al. 2022). Joint site frequency spectra for empirical and inferred data are shown in Figure S7.

Complex model comparisons resulted in a slightly better fit to SFSs than simple models (Tables S6, Figures 4 and S7). For the LYG vs. NJ comparison, GADMA identified allopatric divergence followed by secondary contact at different times as the most probable demographic scenario (log‐likelihood = −160.32; Figure 4A). The split between the north and south groups occurred at approximately 62 Kya. The north group experienced a linear population size decrease, while the south group showed an exponential decline. After an initial period of unidirectional migration from LYG to NJ, bidirectional migration was detected starting around 32 Kya, with higher migration rates from north to south, indicating asymmetrical gene flow. For the DL vs. DS comparison, GADMA inferred an initially low effective population size followed by a population split between DL and DS around 388 Kya (log‐likelihood = −186.08; Figure 4B). After a period of isolation, bidirectional migration was detected starting around 12 Kya, with comparable migration rates in both directions.

### 3.4 Optimal Pelagic Duration Assessment and Larval Dispersal Modelling

The last survival of larvae in each culture plate was used to assess the optimal pelagic duration. All of the larvae in the culture plate at 28°C were dead at 18 days post fertilization (dpf) (Figure S8). At 25°C, all the larvae were dead at 20 dpf. The longest survival of larvae occurred at 17°C at 30 dpf. Larvae survival increased with the decreasing culture temperatures in the settlement assay. This suggested that the optimal pelagic duration of larvae can last 30 days at the appropriate temperature.

**FIGURE 5:**
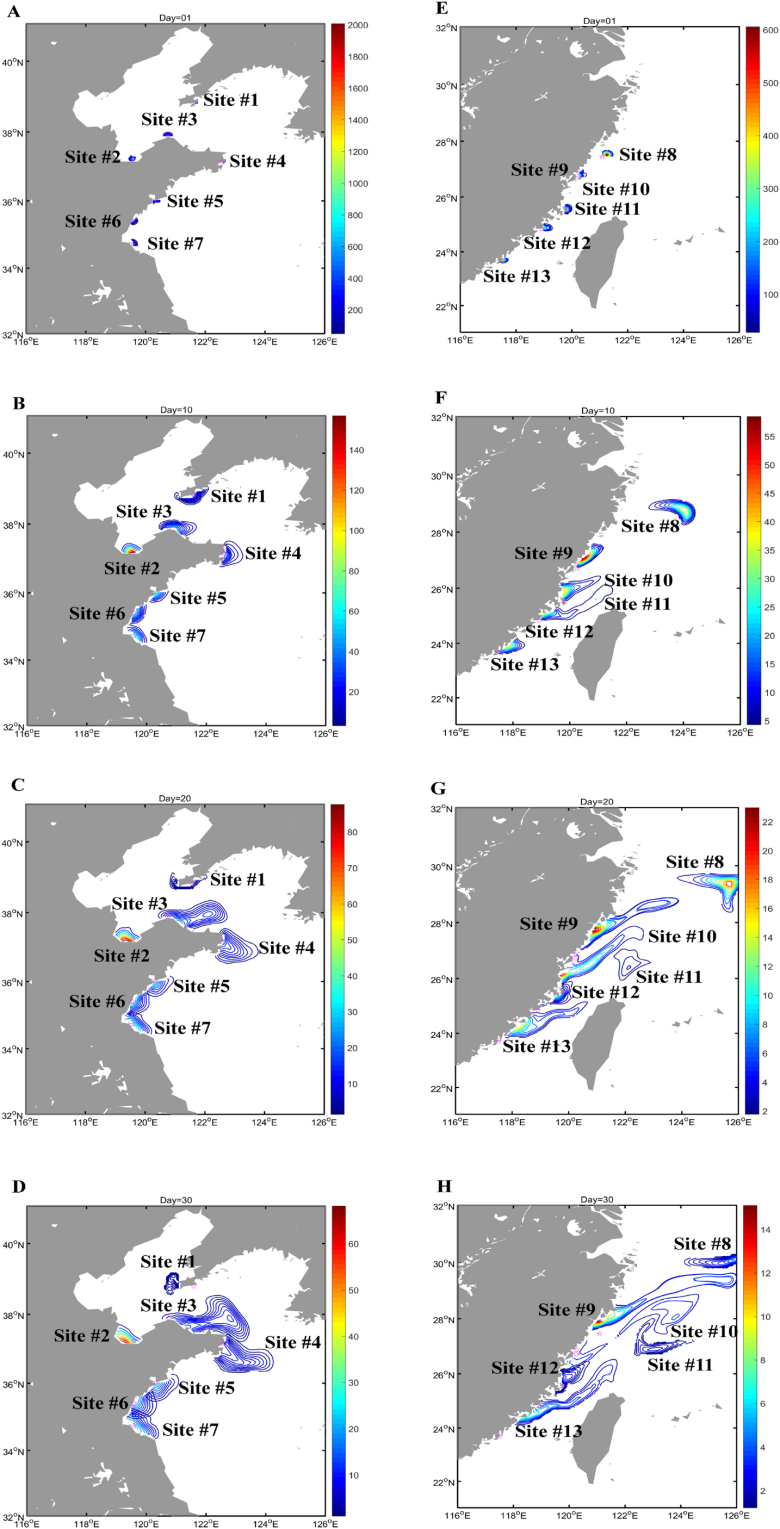
Dispersal path and relative content of particles originating from seven distinct localities within the Northern populations (A–D) and six distinct localities within the Southern populations (E‐H) were modeled using the OGCM for days 1, 10, 20, and 30. For easy identification, Site #1: DL, Site #2: LZ, Site #3: PL, Site #4: RC, Site #5: QD, Site #6: RZ, Site #7: LYG, Site #8: NJ, Site #9: XP, Site #10: LJ, Site #11: PT, Site #12: QZ, Site #13: DS. DL, Dalian; DS, Dongshan; LJ, Lianjiang; LYG, Lianyungang; LZ, Laizhou; NJ, Nanji; PL, Penglai; PT, Pingtan; QD, Qingdao; QZ, Quanzhou; RC, Rongcheng; RZ, Rizhao; XP, Xiapu.

According to the result of population genetic structure, the thirteen populations of *A. rubrolineata* were grouped into two distinct clusters as the origin of the model. The numerical model developed in this study aimed to elucidate the gene flow patterns from an oceanographic perspective and to illustrate how larvae were interconnected by ocean currents. The larvae were treated as passive floating particles within the Ocean General Circulation Model (OGCM), simulating their dispersal during the optimal PLD of 30 days. The complete results of the larval dispersal modelling were shown in Figure 5 and Movies S1,S2. In the Northern populations, larvae from site #6 RZ dispersed to site #7 LYG on day 12. On day 16, larvae from site #5 QD spread to site #6 RZ. Meanwhile, on day 28, larvae from site #3 PL spread to site #4 RC (Figure S9). While in Southern populations, at the very early beginning, larvae from site #9 XP and site #10 LJ met due to the relatively close geographical distance between them (Figure S10). On day 8, larvae from site #12 QZ spread to site #11 PT. On day 24, larvae from site #13 DS spread to site #12 QZ, and larvae from site #9 XP and site #10 LJ spread to site #8 NJ. Notably, the simulated larvae would be relatively faster in the Southern populations due to the stronger northeasterly surface current in July. This result of the simulated larval dispersal model supported the TreeMix gene flow analysis to a considerable extent.

## 4 Discussion

RRS offers a potential avenue for understanding the population structure and demographic history of marine species (Gagnaire et al. 2015). In this study, we investigated the population structure, demographic history, and population connectivity of the chiton *A. rubrolineata* using an integrated approach that combines population genomics, laboratory experiments, and ocean modeling. Our findings highlight the complex interplay between historical and contemporary oceanography, life‐history traits, geographic isolation, and habitat discontinuity that have played important roles in shaping the demographic trajectories and genetic structure of the chiton *A. rubrolineata* .

### 4.1 Genetic Diversity

Genetic diversity plays a pivotal role in enabling natural populations to adapt to environmental fluctuations (Vera et al. 2022). The level of heterozygosity (*H*_O_, *H*_E_) serves as an indicator of the genetic consistency within populations and directly reflects the diversity of genes. Populations with elevated heterozygosity and nucleotide diversity generally indicate a higher degree of genetic variation, which contributes to their genetic robustness and helps them to resist the threat of demographic fluctuations caused by environmental changes (Kool et al. 2013). Overall, both *H*_E_ and *H*_O_ were similar across each population, with thirteen populations demonstrating high levels of genetic variation. In particular, the XP and LJ populations exhibited the highest levels. Nevertheless, the LYG population had the lowest values. Genetic diversity was similar across Northern populations and Southern populations. In general, Southern populations displayed significantly higher genetic diversity than those of Northern populations with higher *H*_E_, *H*_O_, and *π* values. This finding coincides with previous studies by Wang et al. (2019) and Xu, Chu et al. (2020) that investigated the same species along the Chinese coast using a few nuclear and mitochondrial molecular markers. All samples, with the exception of the NJ, XP, and LJ populations, exhibited a consistent, albeit non‐significant, slight heterozygote deficit (*F*_IS_ > 0). This is likely due to a technical genotyping issue associated with null alleles, as previously documented in mollusks (Pino‐Querido et al. 2015).

### 4.2 Population Structure, Gene Flow, and Population Connectivity

The results showed that *F*_ST_ values between intra‐group of Northern and Southern populations were generally less than 0.05, with only three exceptions, indicating low genetic differentiation and high genetic connectivity among these populations. In contrast, *F*_ST_ values between inter‐group of Northern and Southern populations exceeded 0.79, revealing significant genetic differentiation and low genetic connectivity between the two groups. Scatter plots (Figure 2A) further revealed highly differentiated genetic regions between the Northern and Southern groups, distributed across the entire genome. This pattern likely reflects distinct evolutionary pressures or restricted gene flow between the groups. However, within each group (Figure 2B,C), low *F*_ST_ values suggested substantial shared genetic variation, potentially due to recent common ancestry or ongoing gene flow (Gagnaire 2020; Gagnaire et al. 2015). These findings, supported by ADMIXTURE, PCA, and DAPC analyses, have important implications for understanding the species' evolutionary history and adaptive potential.

These results suggest that genetic connectivity and gene flow are limited or at least partially hindered between populations of the two groups, leading to significant population structure. The duration of the larval stage is a critical factor influencing dispersal distance in many marine species (Cowen and Sponaugle 2009). For chiton *A. rubrolineata* , the reproductive cycle occurs from late July to September each year (Yu et al. 2006). During this period, increased freshwater discharge from the Yangtze River reduces estuarine salinity, affecting larval dispersal along coastal currents and impeding gene flow between populations. Additionally, the Subei Shoal in the southwestern Yellow Sea, a unique mudflat zone, acts as a geographical barrier for the rocky‐shore species *A. rubrolineata* along the Chinese coast (Wang et al. 2015). This barrier likely contributes to the long‐term interruption of gene flow between the Northern and Southern populations. TreeMix analysis further supports these findings, revealing possible migration events within the groups but minimal (very weak) gene flow between the Northern and Southern clade. Previous studies have reported similar population structures in chiton *A. rubrolineata* along the Chinese coast, with the Yangtze River Estuary acting as a major divider (Li 2019; Wang 2019; Xu, Chu et al. 2020). Similar patterns have also been observed in other molluscan species, such as *Cellana toreuma* (Dong et al. 2012), *Siphonaria japonica* (Wang et al. 2015) and *Meretrix petechialis* (Wang et al. 2017) using various molecular markers. Given the limited dispersal capacity of chiton *A. rubrolineata* (as inferred from the life‐history traits of many other chiton species), the geographic barrier, and the discontinuous habitat, the significant population structure observed is not surprising.

In contrast to the inter‐group genetic structure, substantial genetic homogeneity was observed within the Northern and Southern groups of chiton *A. rubrolineata* , with no significant population structure detected (Table 2). This homogeneity may primarily result from the absence of dispersal barriers between the Yellow Sea and the Bohai Sea, as well as among the East China Sea. Genetic connectivity within each group is likely facilitated by the present‐day surface currents along the Chinese coastline, which enhance gene flow by transporting planktonic larvae and/or rafting adults among local populations, thereby reducing genetic differentiation. The reproductive cycle of chiton *A. rubrolineata* predominantly occurs in summer, when the East China Sea (ECS) surface current is northeasterly due to the southwest monsoon (Xu, Yang et al. 2020). The Zhejiang–Fujian Coastal Current, which is strong and southward in spring and winter, weakens and turns northward in summer and fall (Lu et al. 2023). Despite the weak northerly currents during the spawning season, most larvae from the Southern ECS are transported into the northern region (Xu, Yang et al. 2020). These larvae from Southern populations disperse along these currents, facilitating gene exchange and resulting in relatively little genetic differentiation between populations. A similar pattern is observed in the Northern populations of chiton *A. rubrolineata* . Moreover, relatively short distances between populations and habitat continuity within each group likely contribute to their genetic homogeneity. The remarkable differentiation between the Northern and Southern populations of chiton *A. rubrolineata* is likely due to population divergence influenced by ocean currents, the Yangtze River discharge, and geographic habitat isolation. This hypothesis is plausible given that pelagic larval dispersal is the primary conduit for contemporary gene flow, thereby shaping the distribution of chiton *A. rubrolineata* . Consequently, we emphasize the importance of in situ conservation for *A. rubrolineata* across its distribution range and along its pelagic larval dispersal route to preserve genetic diversity and evolutionary potential.

### 4.3 Demography History

The DIYABC analysis revealed that the ancestral population of chiton *A. rubrolineata* probably first diversified into two genetic groups at about 1.44–0.89 Mya, with one distributed along the Southern coast and the other along the Northern coast of China. The diversification of *A. rubrolineata* northern and *A. rubrolineata* southern occurred during the Middle Pleistocene epoch (1.8–0.774 Mya), and they passed through the Mid‐Late and Late Pleistocene periods (0.350–0.012 Mya) when sea levels underwent significant and recurrent fluctuations between glacial and interglacial periods. The fall in sea level during the Pleistocene Ice Age interrupted the connection between the marginal seas, limiting the dispersal of populations. Due to long periods of isolation, restricted gene flow leads to significant differentiation between populations and thus to speciation. The Northern and Southern populations of chiton *A. rubrolineata* underwent expansions around 0.86–0.44 Mya and 0.96–0.52 Mya, respectively. These expansions corresponded with the Dagu‐Mount Lu Interglacial of the Quaternary Interglacial in China, a finding consistent with previous research based on COI and 16S rRNA sequences (Xu, Chu et al. 2020). Following these expansions, continuous gene flow between the populations resulted in a genealogical contact zone in the adjacent areas between the Bohai Sea and the Yellow Sea, as well as within the East China Sea. Consequently, no significant genetic structure was observed between the Northern and Southern populations. In conclusion, *A. rubrolineata* northern and *A. rubrolineata* southern groups can be robustly separated with strong population genetic and demographic evidence.

GADMA detected low migration rates from north to south around 62 Kya, likely due to geographic proximity. However, our demographic model also revealed an asymmetric migration signal around 32 Kya, with higher migration rates from north to south than from south to north. This pattern can be explained by the changing cycles during the Pleistocene epoch (Ludt and Rocha 2015). For example, coastal areas contracted during sea level falls but expanded again during subsequent sea level rises (Ludt and Rocha 2015). Our demographic model also indicated population expansions of both DL and DS around 401 Kya, which likely increased the chances of contact between the north and south. Secondary contact around 12 Kya can be attributed to changes during the last glacial period (LGP, 110–115 Kya) (Severinghaus and Brook 1999). The abrupt climate change and subsequent sea‐level fluctuations during the LGP significantly influenced the demographic history of contemporary marine species globally (Hewitt 2004) and were particularly impactful for marine species in this region (Ni et al. 2020). Despite secondary contact, the long allopatric period between the north and south might have been sufficient to accumulate divergence through genetic drift. This conclusion is supported by the presence of high *F*_ST_ values across the entire genome, rather than in specific regions, indicating that genome‐wide divergence had been accumulating over an extended period (Feder et al. 2012).

Although our results from DIYABC and GADMA did not reveal identical demographic patterns in recent times, these discrepancies may stem from the inherent limitations of both programs. For instance, GADMA assess multiple models involving changes in populations size and various migration events using the best likelihood score and associated parameters, minimizing the number of parameters to reduce the risk of overfitting the model to the JSFS data (Noskova et al. 2022, 2020). In contrast, DIYABC enables the consideration of complex population histories, including any combination of population divergence events, admixture events and changes in past population size based on a priori hypotheses, and then different evolutionary scenarios are evaluated (Cornuet et al. 2014). Despite these differences, both approaches agreed on key aspects: a smaller effective ancestral population size relative to the simulated derived populations, and multiple but weak gene flow events between the two simulated populations.

There were some debates about the divergence time between the Northern and Southern groups of chiton *A. rubrolineata* , which was approximately 1.44–0.89 Mya as estimated in this study. This finding contradicts a study published earlier in 2019, which showed that the estimated divergence time was 216.99–204.37 Mya as inferred by molecular clock and coalescence theory (Li 2019). However, the estimates may be subject to significant errors since the calibration points retrieved from other published papers were not accurate. The choice of calibrations is an experimental decision that has a major impact on the divergence time estimate (Villaverde et al. 2021). Furthermore, multiple calibrations seem to provide more robust estimates than single or few calibrations (Duchêne et al. 2014). Therefore, the choice of calibration points should be made cautiously and meticulously. The divergence time between *A. rubrolineata* northern and *A. rubrolineata* southern is comparable to other intertidal mollusks (*Coelomactra antiquata* (Kong and Li 2009), *Atrina pectinata* (Liu et al. 2011)), whose intraspecific lineage differentiation was initiated a few million years ago.

### 4.4 Speciation With Gene Flow in the Chiton *A. rubrolineata*

Traditional views consider marine environments as open systems due to the lack of physical barriers and the extensive dispersal capabilities afforded by prolonged larval stages in many marine organisms (Palumbi 1992). However, empirical studies have revealed diversification without complete isolation and the prevalence of cryptic speciation in marine settings (Bierne et al. 2003; Peres et al. 2022). Indeed, diversification processes are marked not solely by stringent isolation but also encompass scenarios involving isolation coupled with migration, secondary contact, or intermittent connectivity (De Jode et al. 2023). The interplay between geographic barriers and dispersal potential can result in speciation with gene flow as a likely scenario in marine species (Potkamp and Fransen 2019), which also seems to be the case for chiton *A. rubrolineata* .

Speciation is often a continuous process leading to complete reproductive isolation (Abbott et al. 2013). Under a general model of speciation with gene flow, the initial stage involves positive selection acting on a limited number of genes, while most of the genome remains largely undifferentiated (Feder et al. 2012). Subsequently, divergence hitchhiking creates “islands of differentiation”, eventually leading to substantial genome‐wide differentiation (Gagnaire et al. 2015; Meyer et al. 2024). In our study, genomic differentiation across the genome exhibits multiple highly divergent regions and can be considered an early/intermediate moment in the speciation process. Therefore, the *F*_ST_ values not only show levels of differentiation consistent with different species (> 0.80), but the population structure also appears to show a similar pattern, suggesting undocumented speciation within the chiton *A. rubrolineata* .

### 4.5 Conservation Implications

Understanding genetic diversity and connectivity of marine populations is crucial not only for improving our basic understanding of evolution, but also for establishing conservation priorities (Allendorf et al. 1997; Fraser and Bernatchez 2001; Gagnaire et al. 2015). Based on our findings, the chiton *A. rubrolineata* (Lischke 1873) should be divided into two lineages (i.e., northern and southern lineages), occupying different latitudinal ranges along the Chinese coast, as a result of life‐history traits and along with local oceanographic features. Therefore, it is imperative to manage the chiton *A. rubrolineata* populations from the northern and southern regions according to the genetic differentiation pattern identified in this study. Recent molecular phylogenetic analyses have elucidated cryptic diversity within polyplacophorans (Choi et al. 2021; Ibáñez et al. 2019). We believe that there may be other cases in marine invertebrates that have yet to be discovered. In conclusion, our data provide a valuable first step for future studies of the genetic basis of adaptation and conservation in the chiton *A. rubrolineata* species.

## 5 Conclusions

In this study, we conducted RRS of chiton *A. rubrolineata* from thirteen regions along the Chinese coast to investigate the genetic diversity and population structure. Our analyses of genome‐wide SNPs revealed substantial genetic divergence between Northern and Southern populations. Our findings through empirical and numerical results also revealed that the interplay of historical and contemporary oceanography, life‐history traits, Changjiang Diluted Water, and Subei Shoal has played an important role in shaping the demographic trajectories and genetic structure of chiton *A. rubrolineata* , revealing the presence of two separately evolving lineages. In conclusion, these results deepen our understanding of genetic diversity in polyplacophoran molluscs and provide a guideline for future analyses of non‐model and potentially threatened species, and will contribute to biodiversity conservation.

## Conflicts of Interest

The authors declare no conflicts of interest.

## Supporting information


Data S1.

**Movies S1.** Dispersal path and relative content of particles originating from seven distinct localities within the Northern populations were modeled using the OGCM over 30 days. From top to bottom, the locations marked with pink pentagon stars are as follows: DL, Dalian; LYG, LianyungangLZ, Laizhou; PL, Penglai; QD, Qingdao; RC, Rongcheng; RZ, Rizhao.
**Movie S2.** Dispersal path and relative content of particles originating from six distinct localities within the Southern populations were modeled using the OGCM over 30 days. From top to bottom, the locations marked with pink pentagon stars are as follows: DS, Dongshan; LJ, Lianjiang; NJ, Nanji; PT, Pingtan; QZ, Quanzhou; XP, Xiapu.

## Data Availability

Data is publicly accessible at GenBank under Bioproject accession number PRJNA1032285 and Biosample accession numbers SAMN38150271–SAMN38150530.
