# Supplementary material for: The Intertidal North‐South Split: Oceanographic Features and Life History Shape the Phylogeography of Chiton Acanthochitona rubrolineata
Source: Evol Appl. 2025 Mar 31;18(4):e70095. doi: 10.1111/eva.70095 (PMC11955844; doi:10.1111/eva.70095)
Supplement: Supplementary file 1 — Data S1. [file EVA-18-e70095-s002.docx]

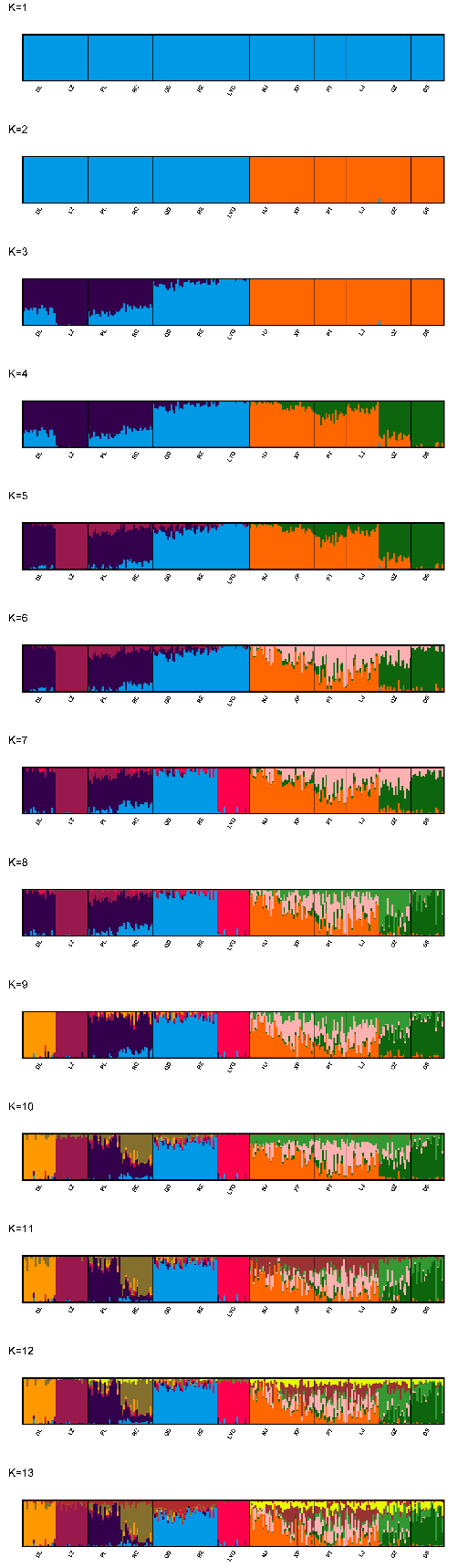


Figure S1 CLUMPAK major mode plots for the ADMIXTURE results (K = 1 to 13 after the removal of spurious clusters) based on neutral SNPs.

DL: Dalian, LZ: Laizhou, PL: Penglai, RC: Rongcheng, QD: Qingdao, RZ: Rizhao, LYG: Lianyungang, NJ: Nanji, XP: Xiapu, PT: Pingtan, LJ: Lianjiang, QZ: Quanzhou and DS: Dongshan.


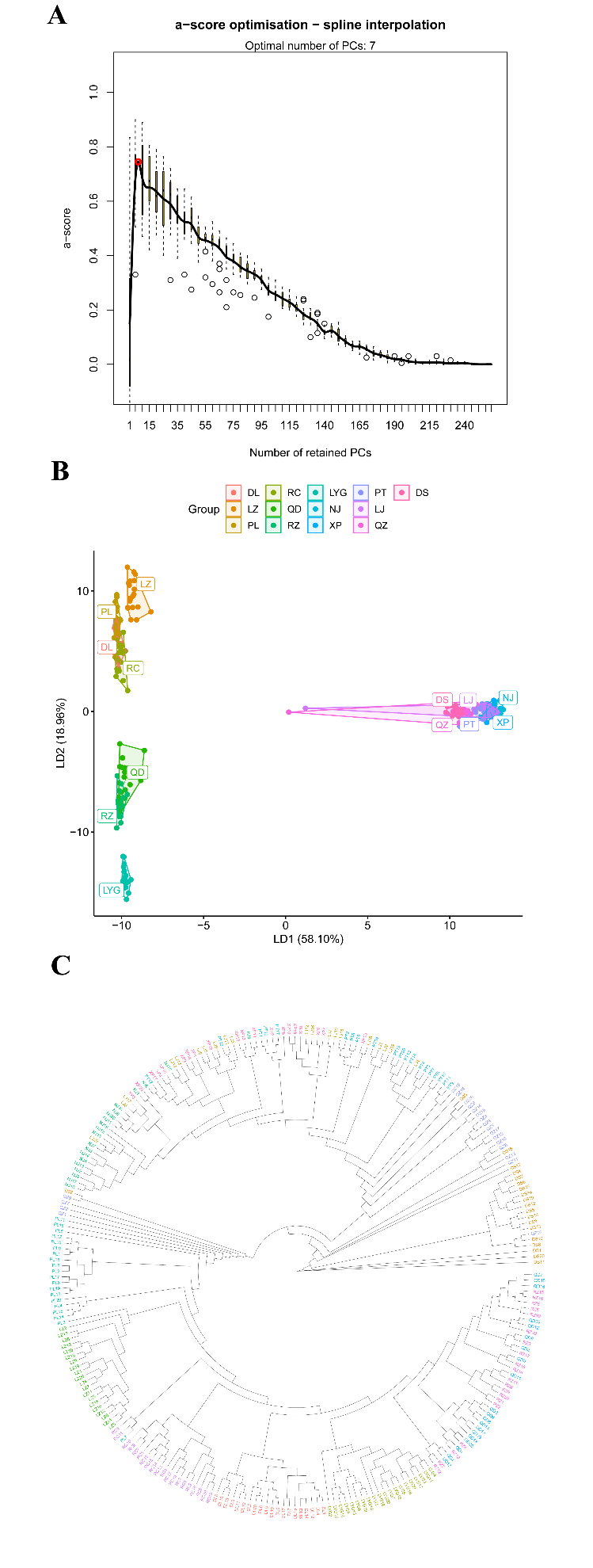


## Figure S2 Population genetic structure based on neutral loci in for thirteen populations. (A) a-score result. (B) Discriminant analysis of principal components (DAPC) describing the variation between the sampling sites. Inertia ellipses encapsulate the variation per sampling site, which the eigenvalues for the first seven PCs are depicted in bar plot insets. Each individual represented by a unique color label corresponding to their respective populations of origin. (C) Neighbor-Joining phylogenetic tree constructed based on p-distances between each pair of individuals.

DL: Dalian, LZ: Laizhou, PL: Penglai, RC: Rongcheng, QD: Qingdao, RZ: Rizhao, LYG: Lianyungang, NJ: Nanji, XP: Xiapu, PT: Pingtan, LJ: Lianjiang, QZ: Quanzhou and DS: Dongshan.


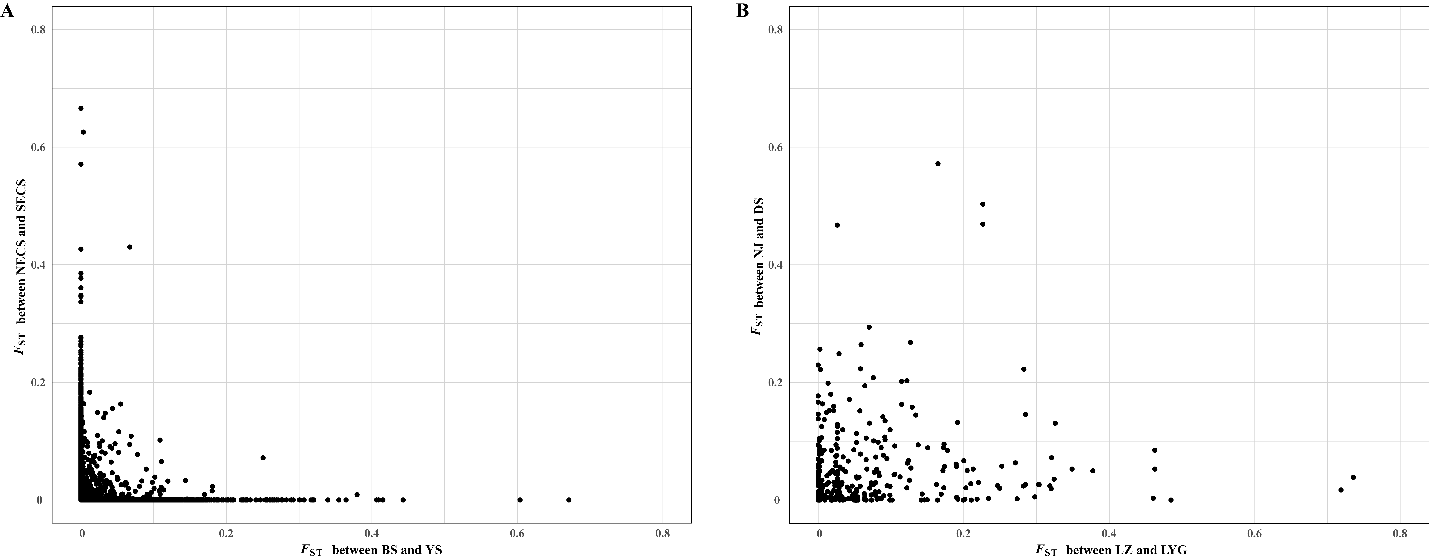


## Figure S3 Plotting of the *F*_ST_ - *F*_ST_ coplots between the Bohai Sea (BS) and Yellow Sea (YS) clusters, and Northern (NESC) and Southern East China Sea (SESC) clusters (A), and between LZ and LYG populations, and NJ and DS populations (B). Each dot is a SNP marker.

LZ: Laizhou, LYG: Lianyungang, NJ: Nanji, and DS: Dongshan.


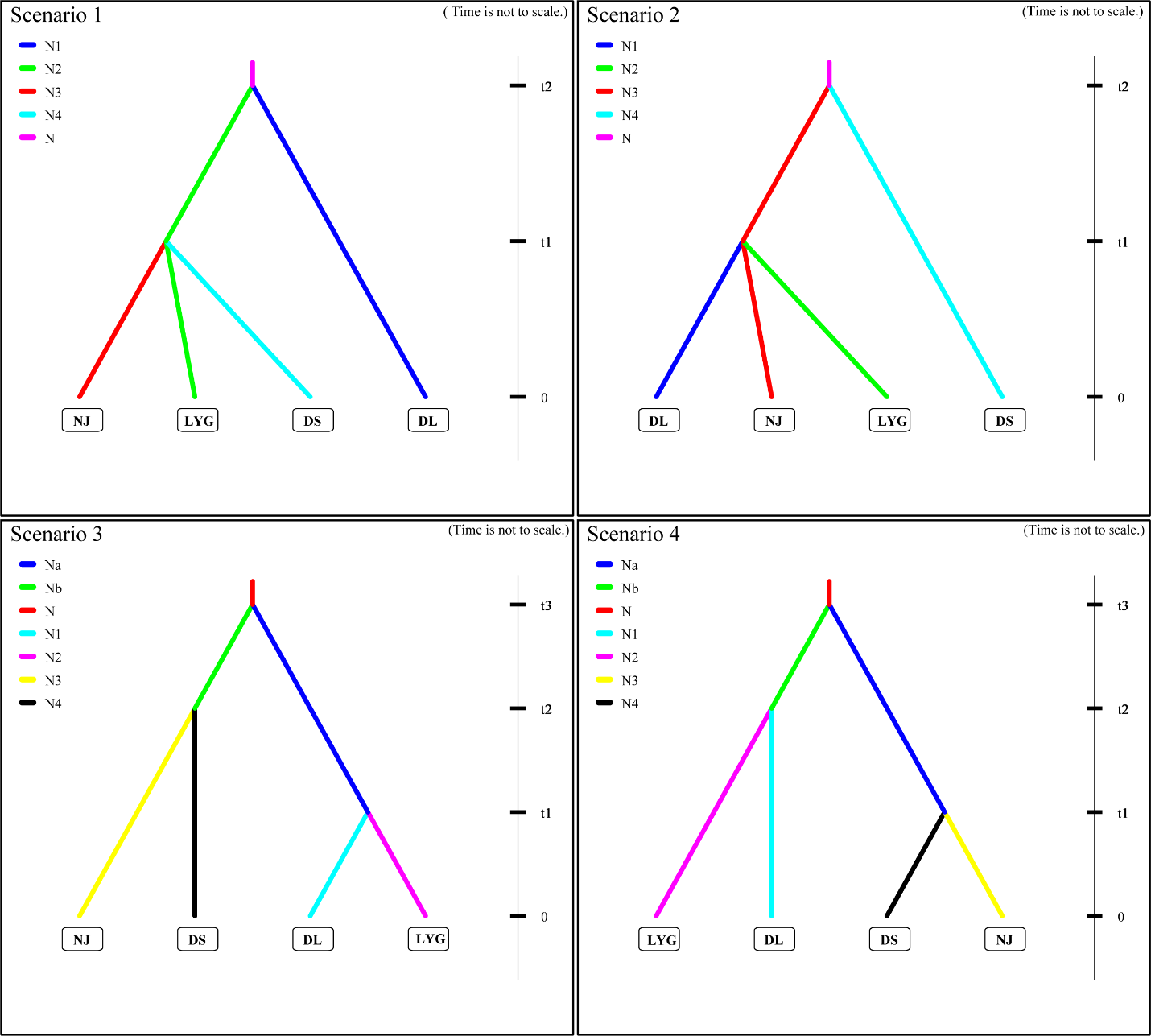


Figure S4 Four scenarios simulated in DIYABC to evaluate the population demographic history of *A. rubrolineata*.

In the scenario, t# represents the time-scale in terms of the number of generations, and N# represents the effective population size of an unknown ancestral population. N1: effective population size (*N*_e_) of DL; N2: *N*_e_ of LYG; N3: *N*_e_ of NJ; N4: *N*_e_ of DS. Set t3 > t2 > t1.

DL: Dalian, LYG: Lianyungang, NJ: Nanji, DS: Dongshan.

Scenario 1: Two populations of DL and LYG diverged at time t2 from an ancestral population of size N. At time t1, NJ and DS populations were derived from LYG population.

Scenario 2: Two populations of NJ and DS diverged at time t2 from an ancestral population of size N. At time t1, DL and LYG populations were derived from NJ population.

Scenario 3: The common ancestral population of the Northern (*N*_a_) and Southern groups (*N*_b_) diverged at time t3 from an ancestral population of size N. Then the Southern group populations were simultaneously derived from the common ancestral population (*N*_b_) at time t2. The Northern group populations were simultaneously derived from the common ancestral population (*N*_a_) at time t1.

Scenario 4: The common ancestral population of the Northern (*N*_a_) and Southern groups (*N*_b_) diverged at time t3 from an ancestral population of size N. Then the Northern group populations were simultaneously derived from the common ancestral population (*N*_a_) at time t2. The Southern group populations were simultaneously derived from the common ancestral population (*N*_b_) at time t1.


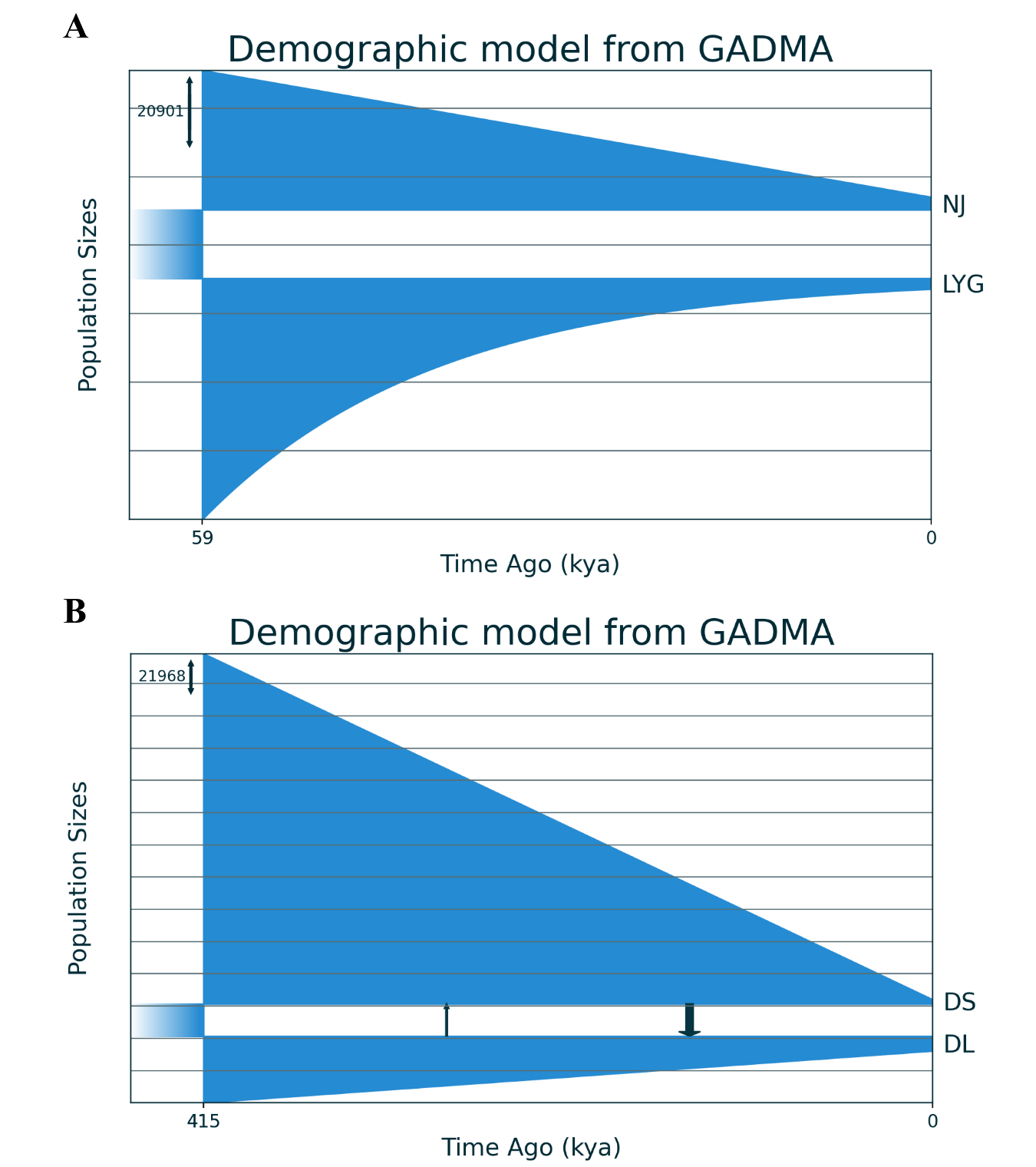


## Figure S5 Best ranked ‘simple’ demographic models describing divergence and changes in population size between (A) LYG (north) and NJ (South), and (B) DL (north) and DS (South). Demographic models were constructed using the diffusion approximation method (*moments*; Jouganous et al., 2017) implemented in the program GADMA2 (Noskova et al., 2022). Joint site frequency spectra for empirical and inferred data are shown in Figure S6.


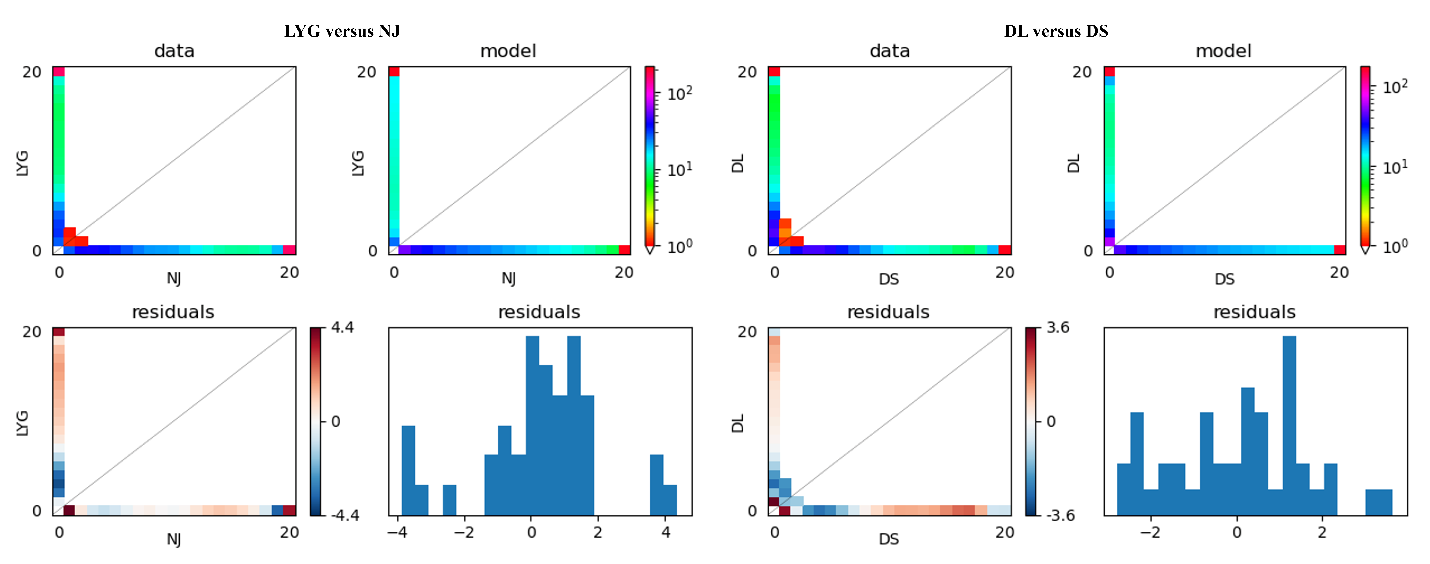


## Figure S6 Empirical and inferred joint site frequency spectra (top row) and residual plots (bottom row) for each replicate comparison (LYG versus NJ and DL versus DS) with ‘simple’ model. Inferred joint site frequency spectra were constructed using the diffusion approximation method (*moments*; Jouganous et al., 2017) implemented in the program GADMA2 (Noskova et al., 2022).


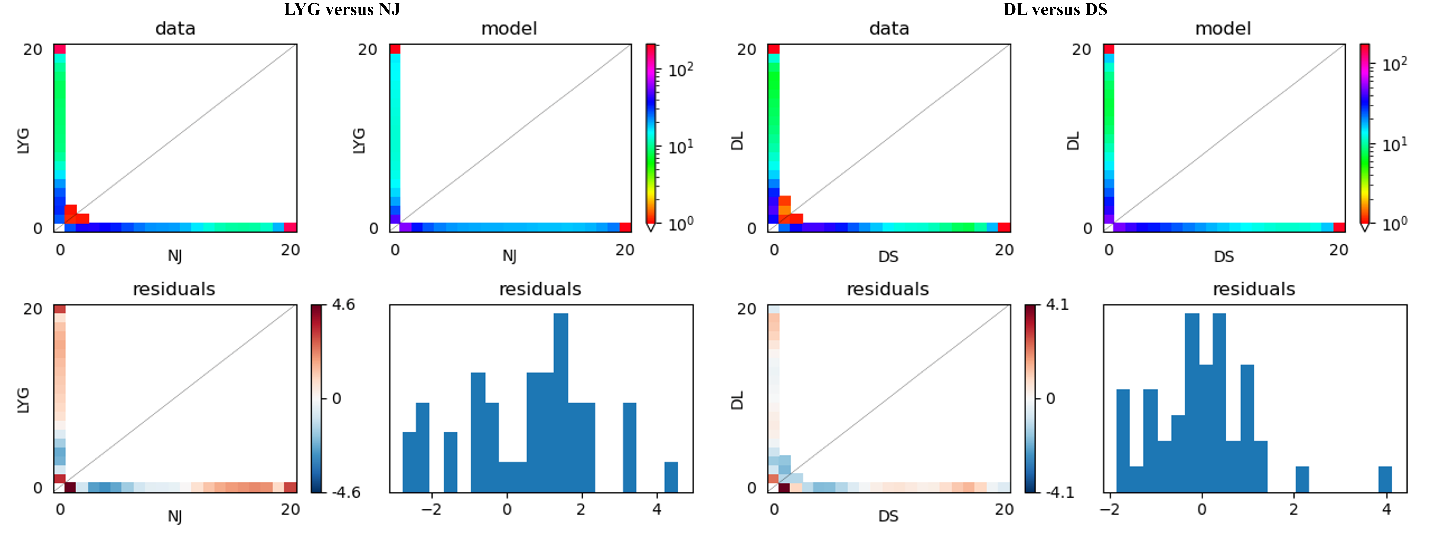


## Figure S7 Empirical and inferred joint site frequency spectra (top row) and residual plots (bottom row) for each replicate comparison (LYG versus NJ and DL versus DS) with ‘complex’ model. Inferred joint site frequency spectra were constructed using the diffusion approximation method (*moments*; Jouganous et al., 2017) implemented in the program GADMA2 (Noskova et al., 2022).

## Figure S8 Observed planktonic larval durations (PLD) represented by survival days for chiton *A. rubrolineata* at different culture temperatures.


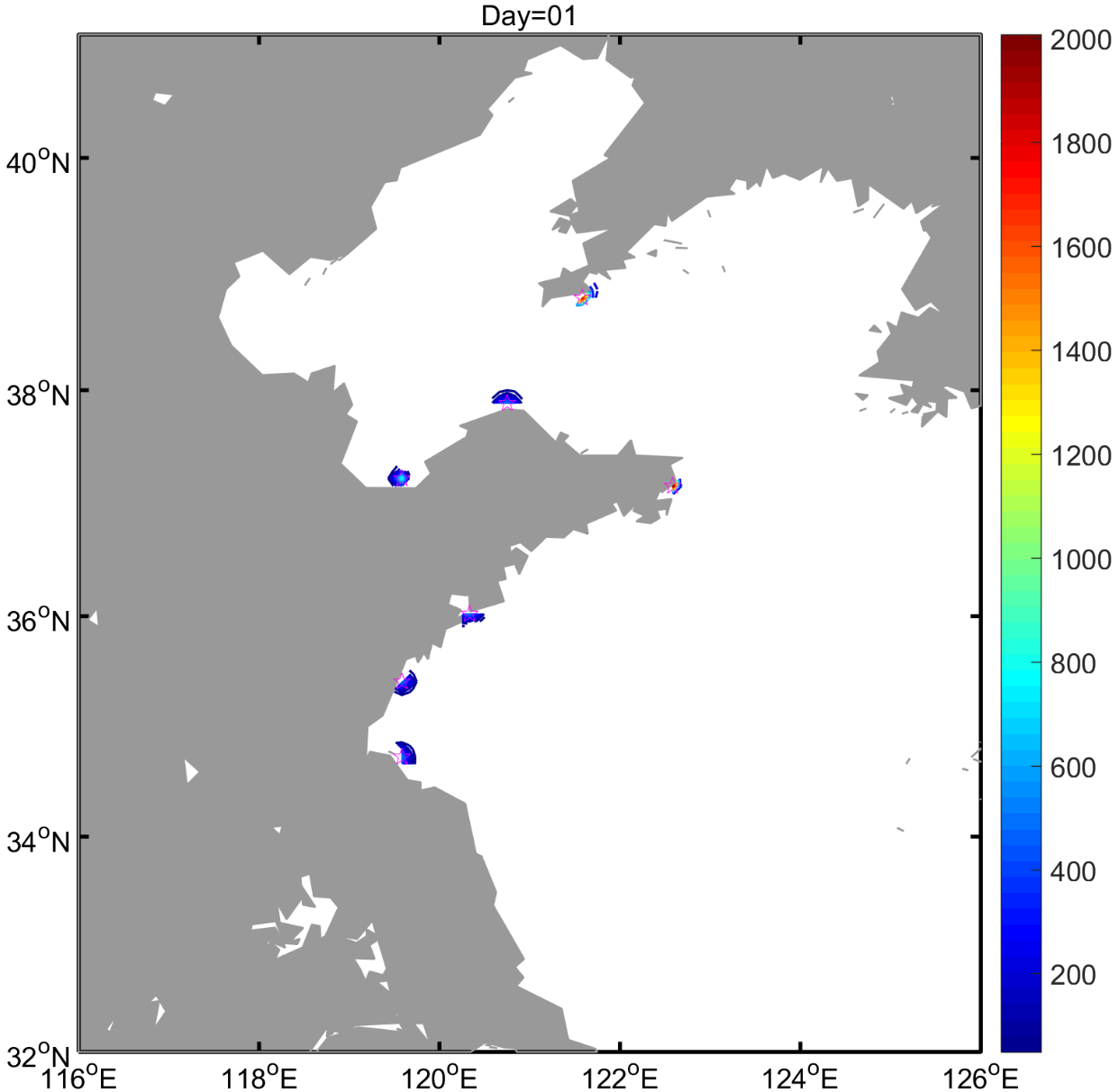

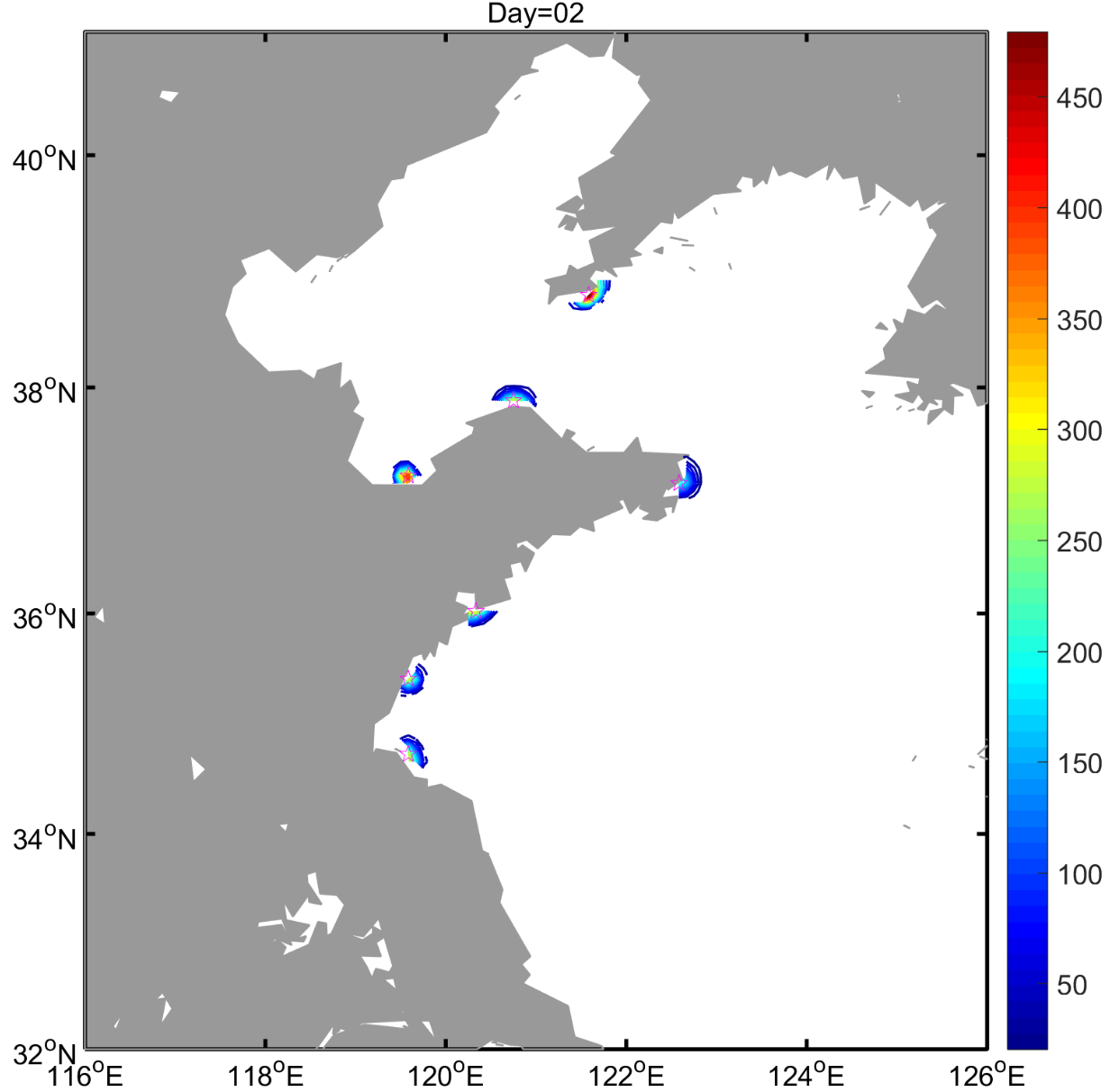

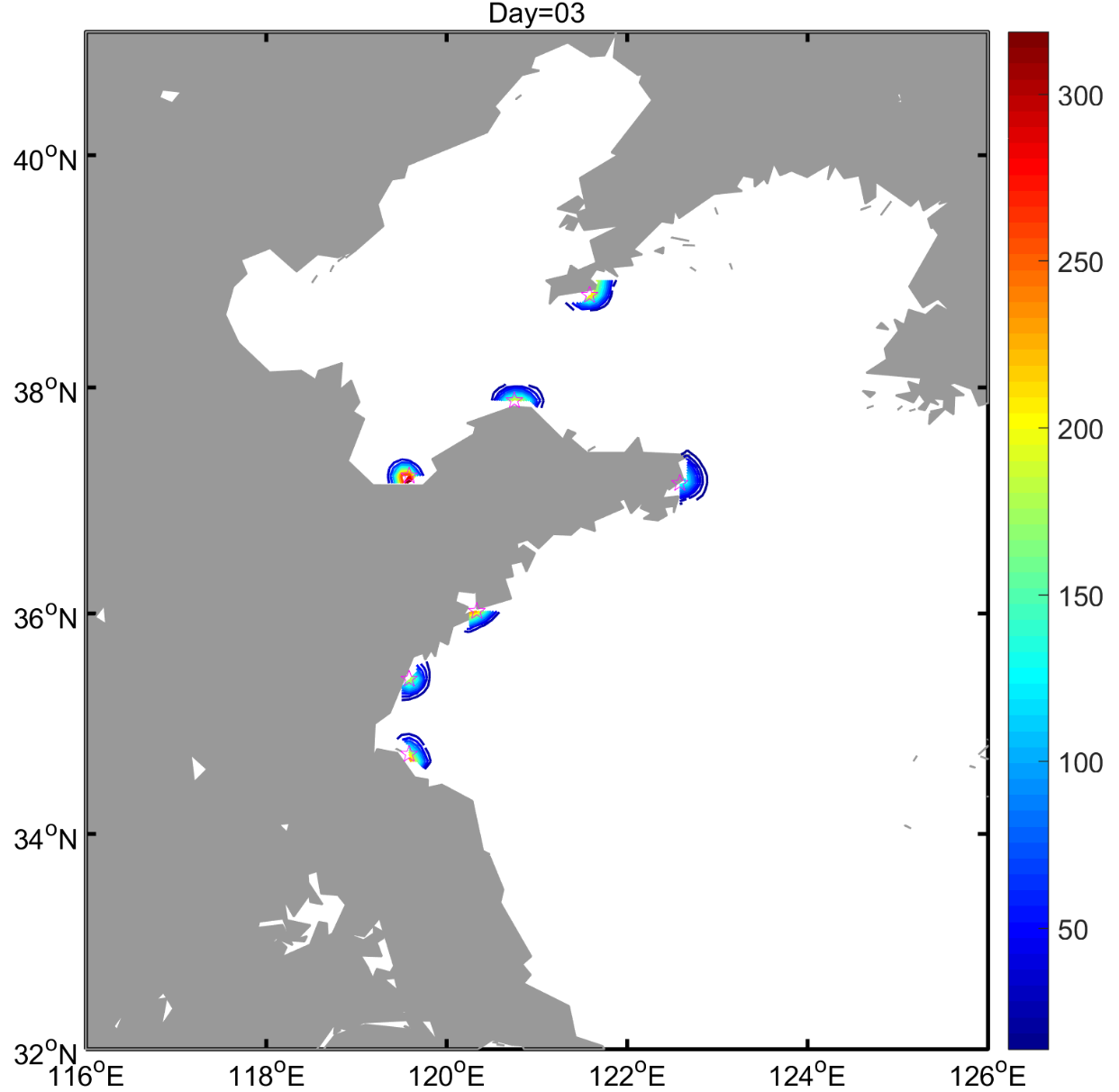

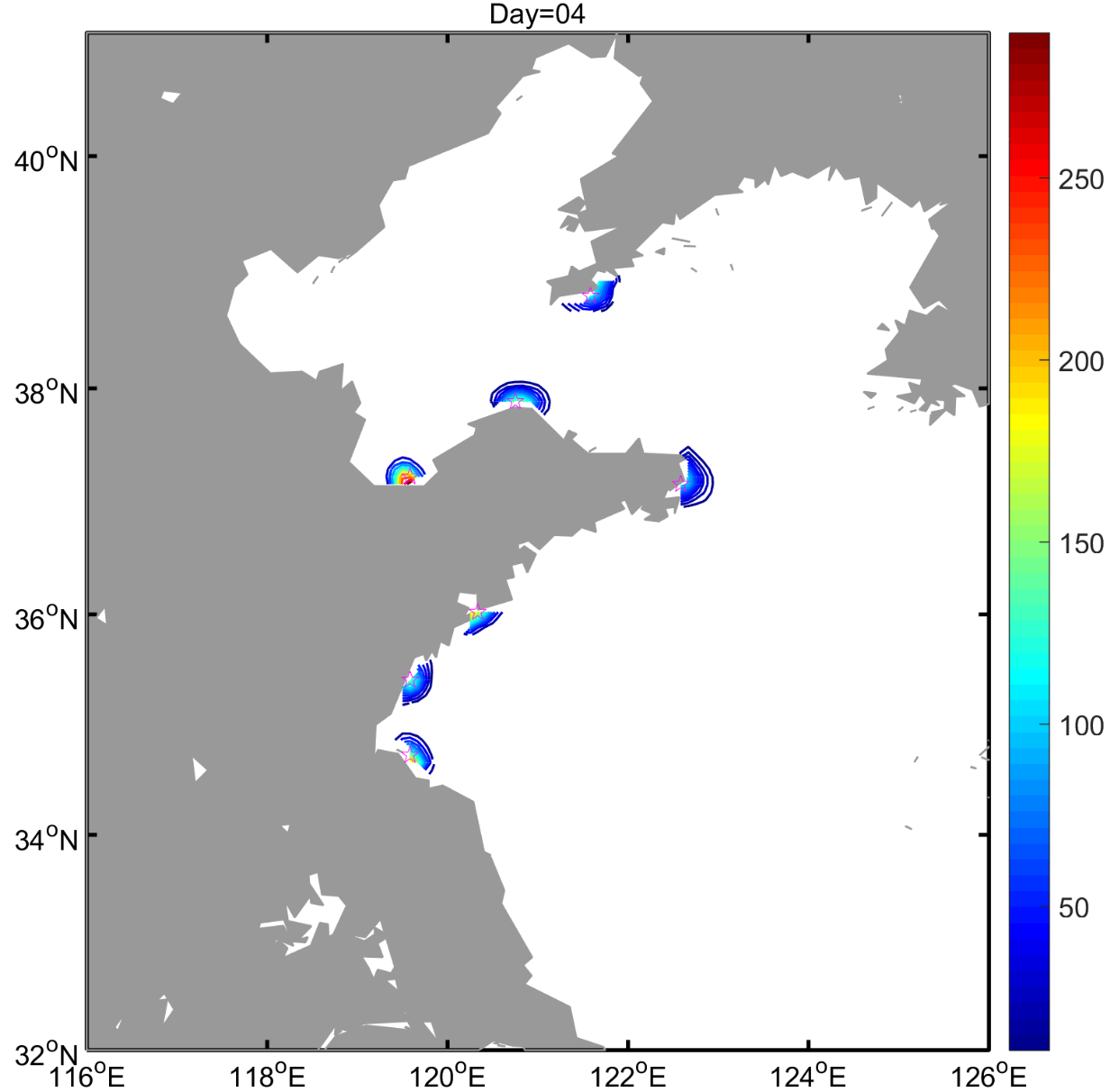

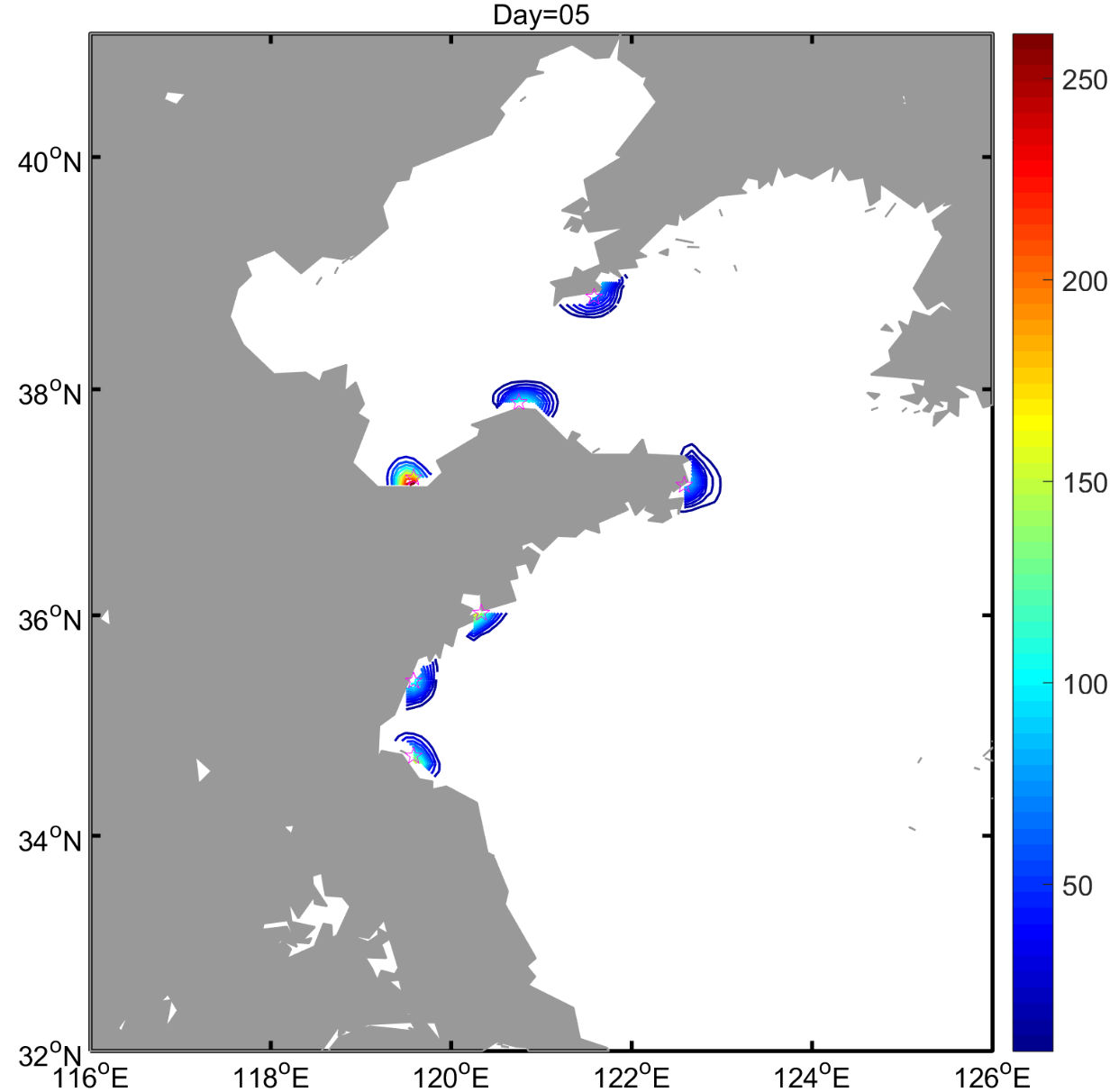

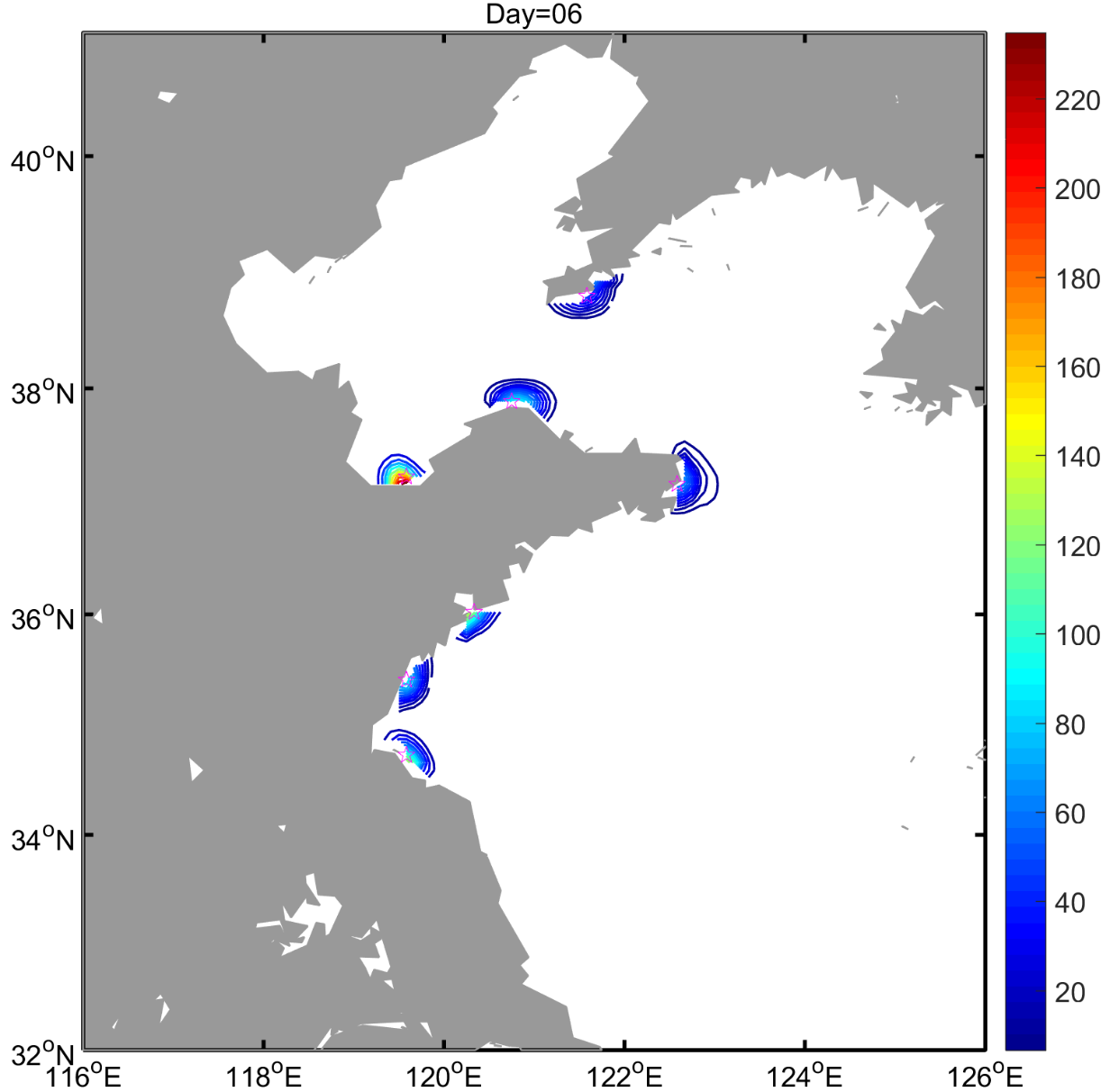

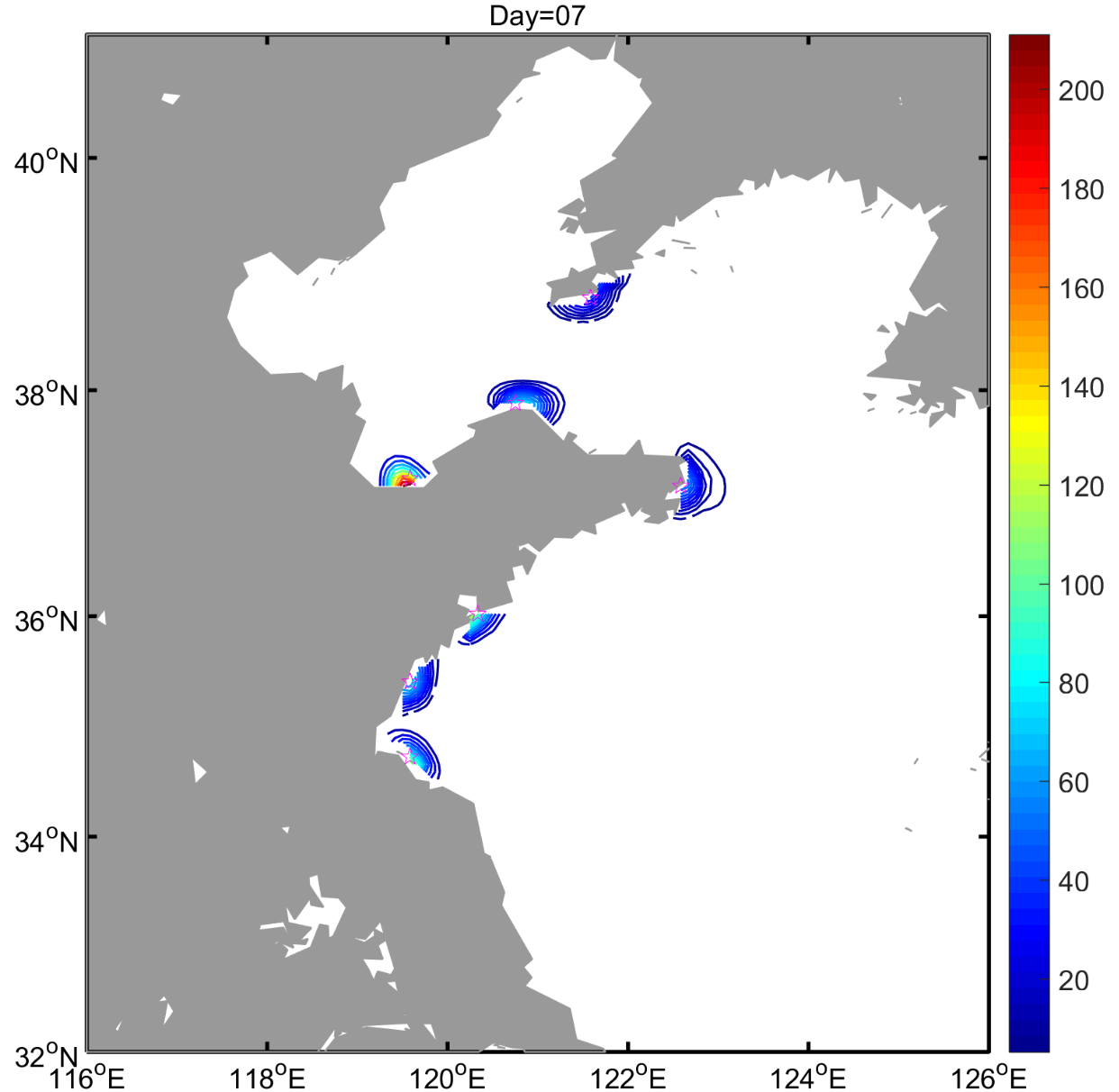

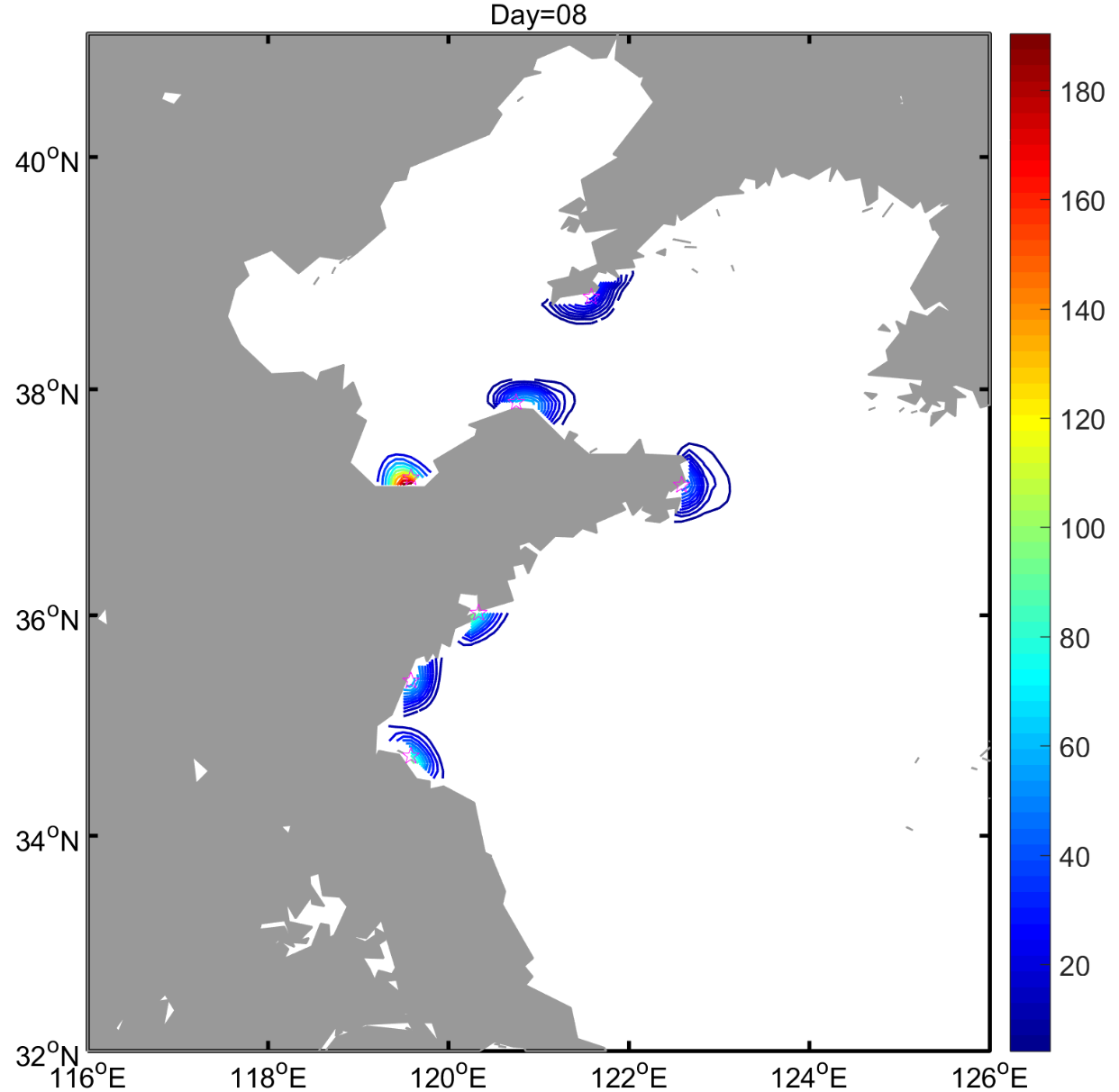

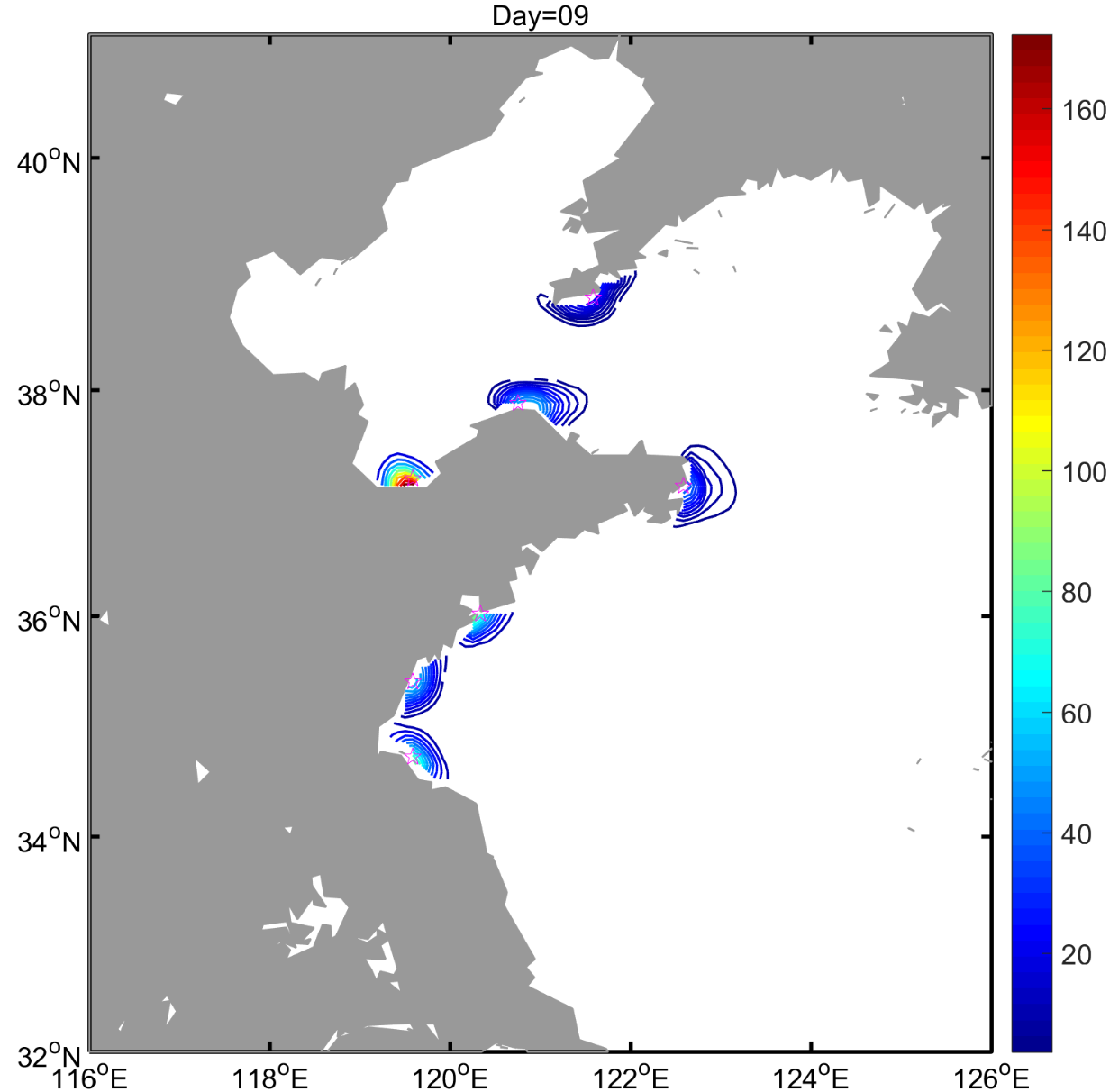

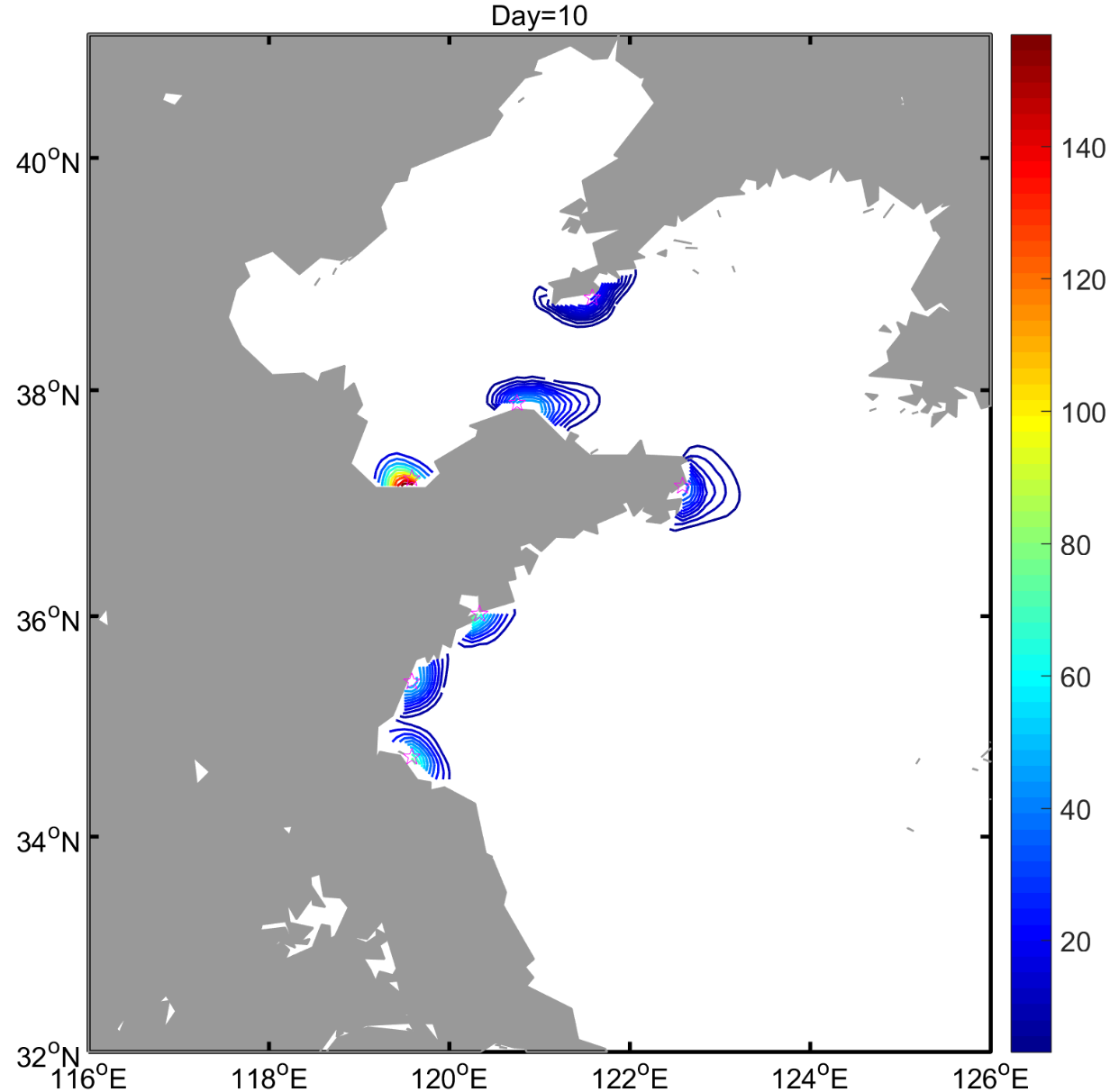

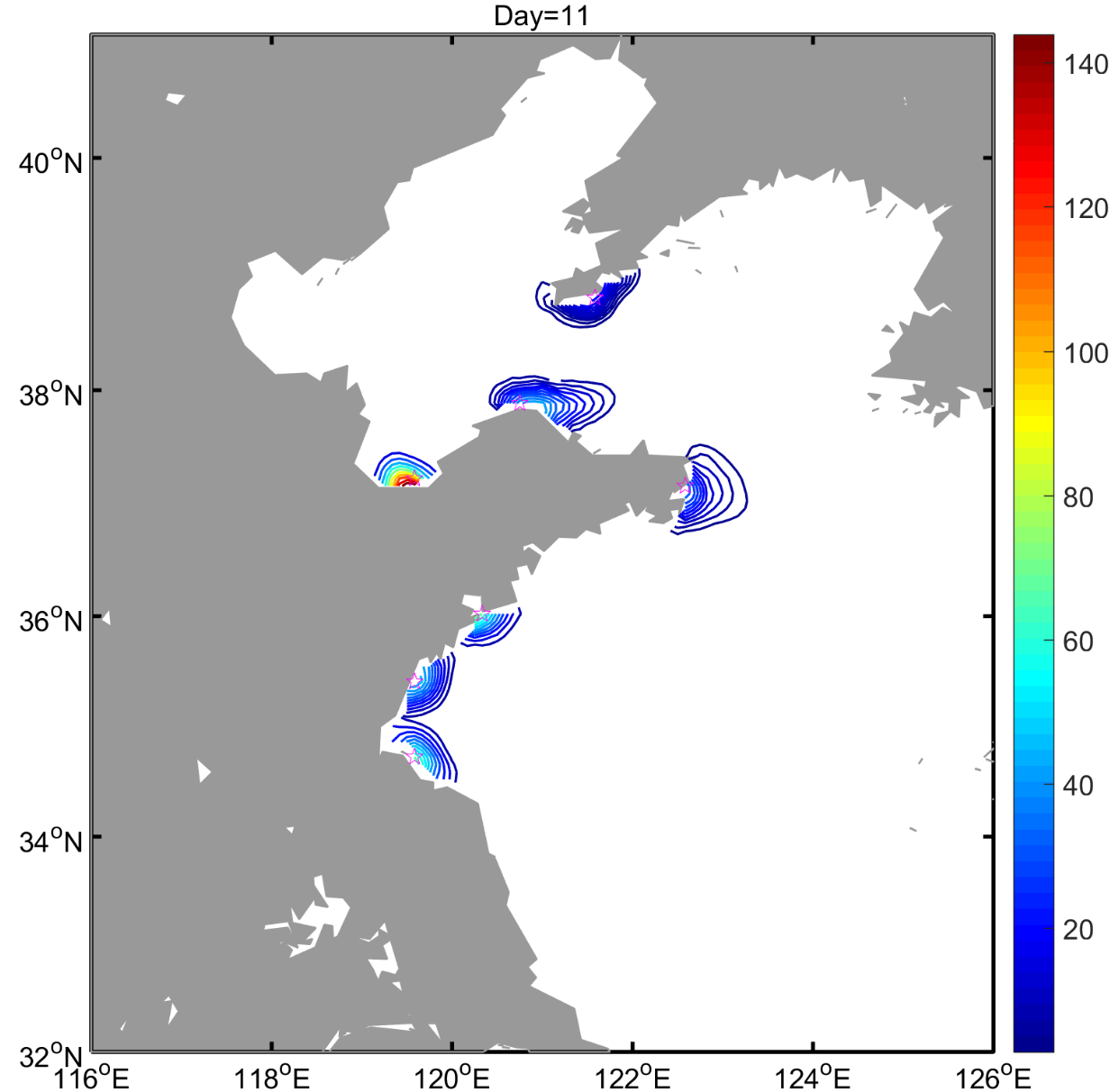

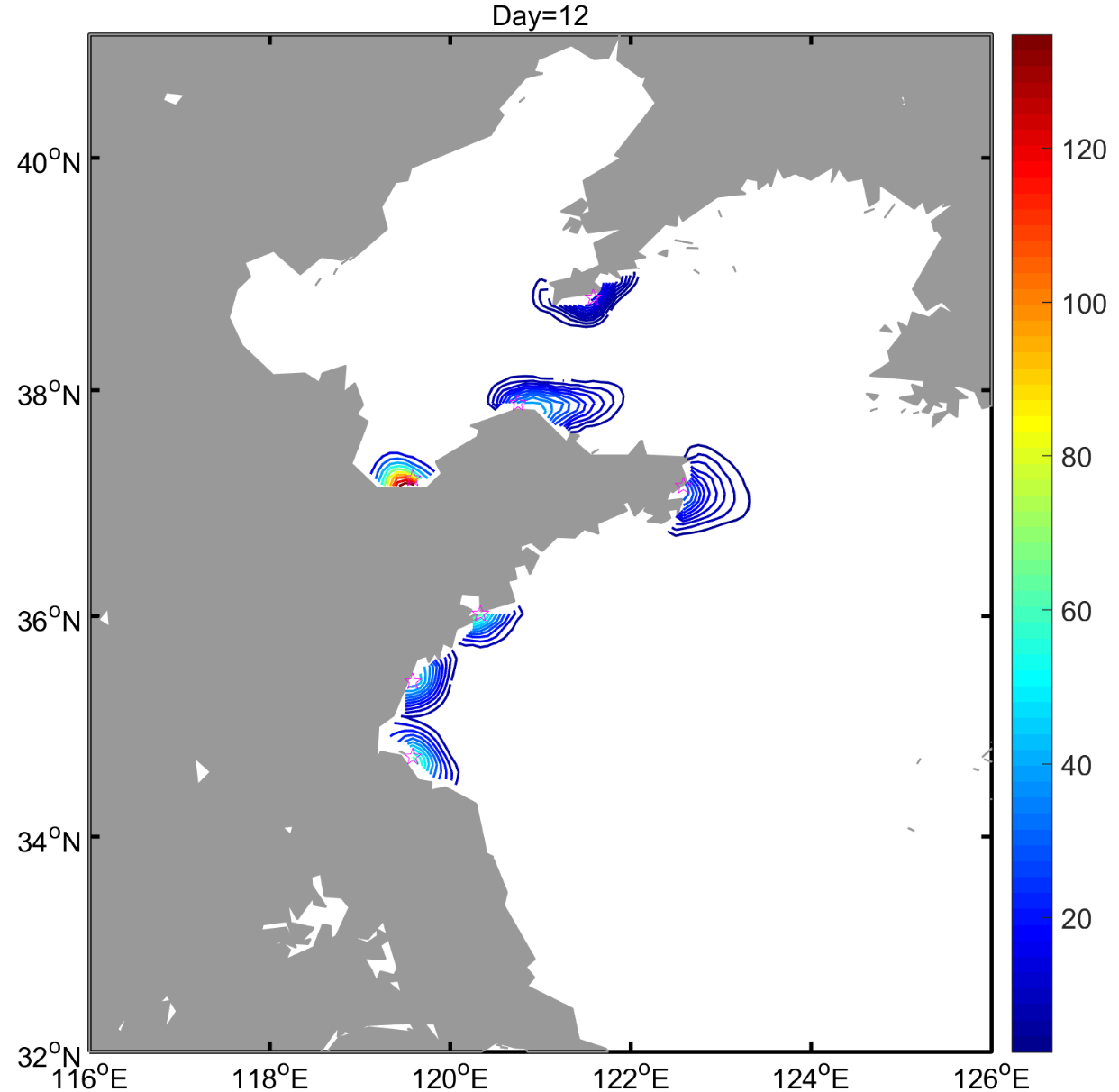

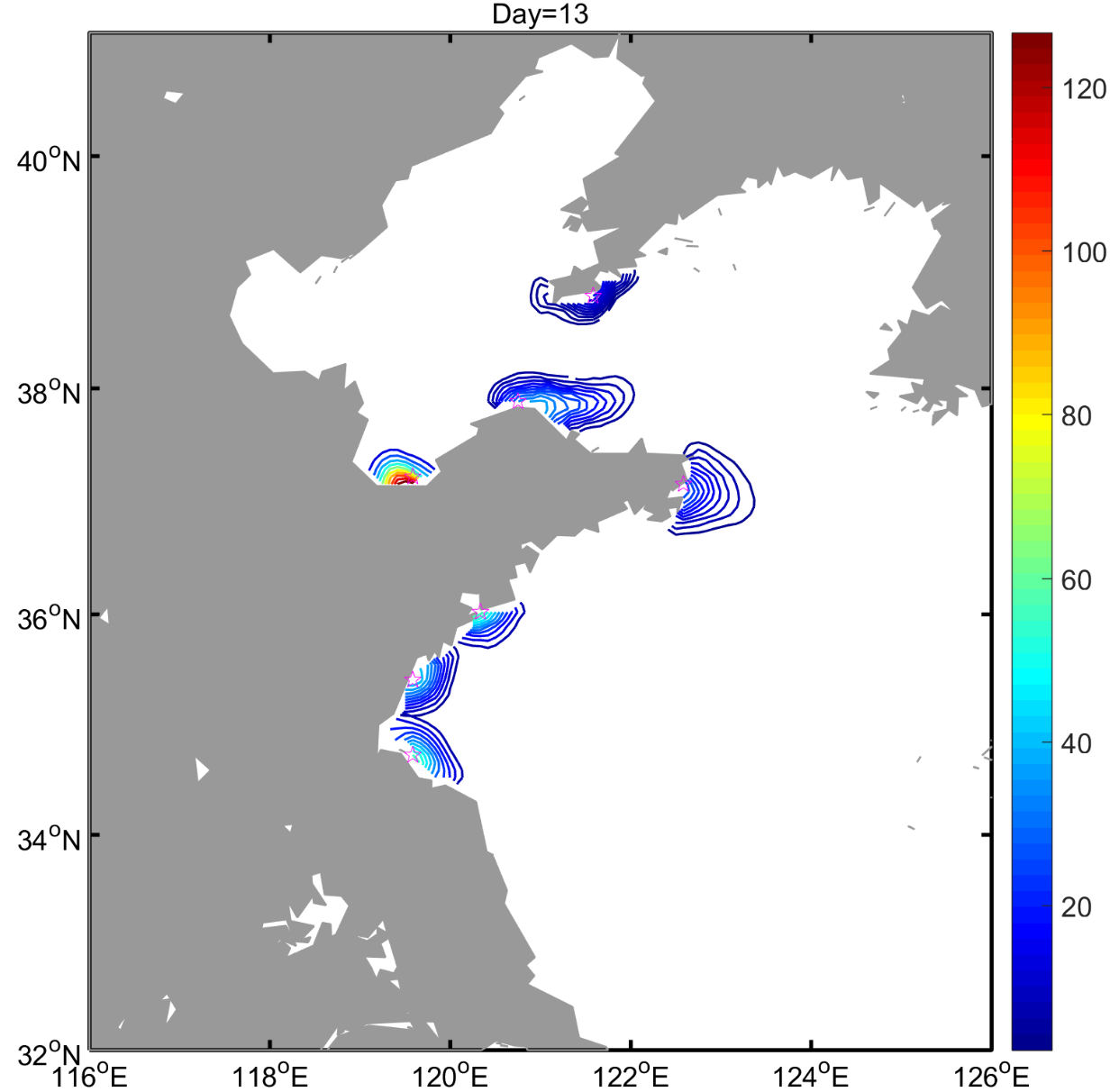

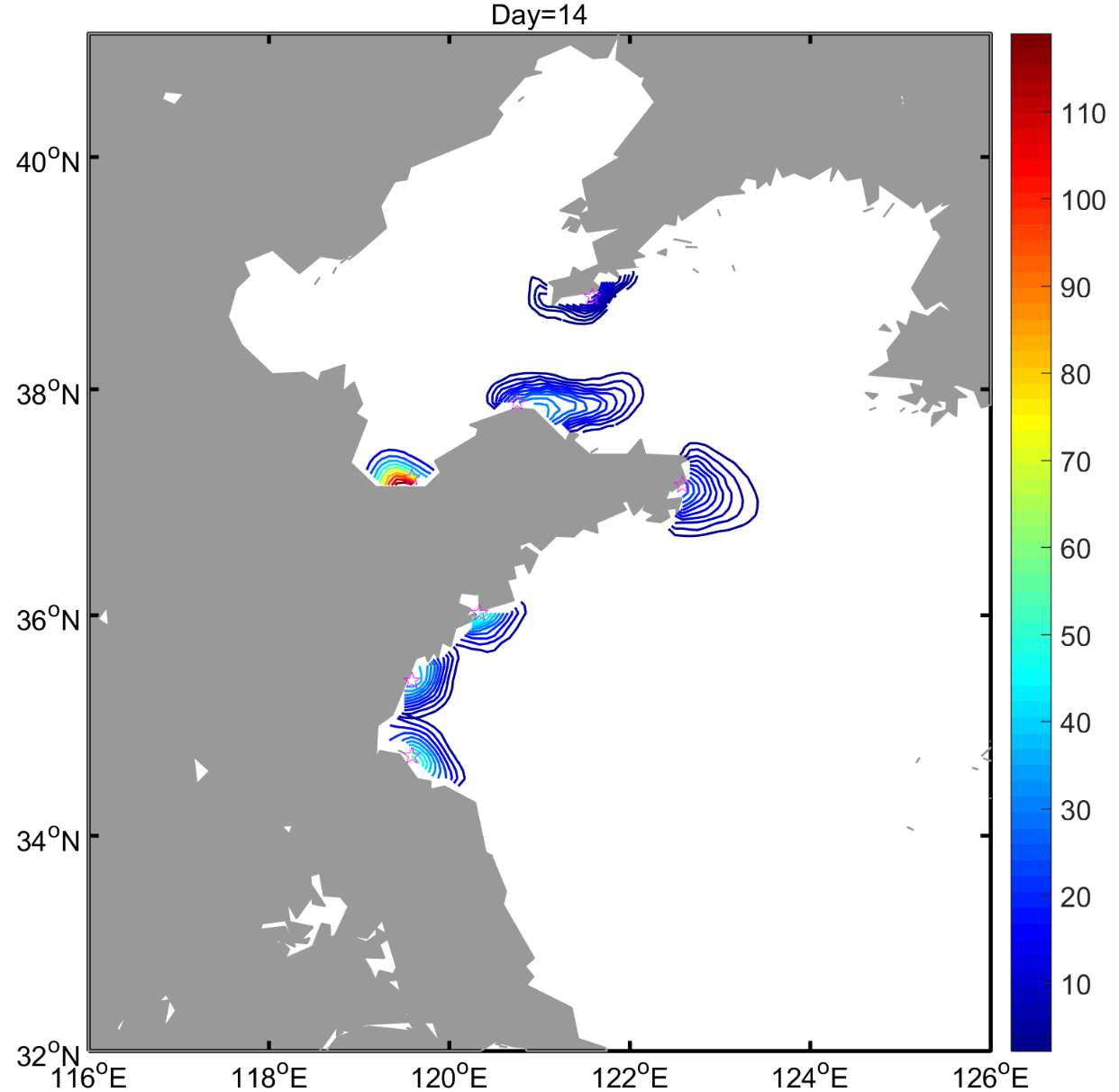

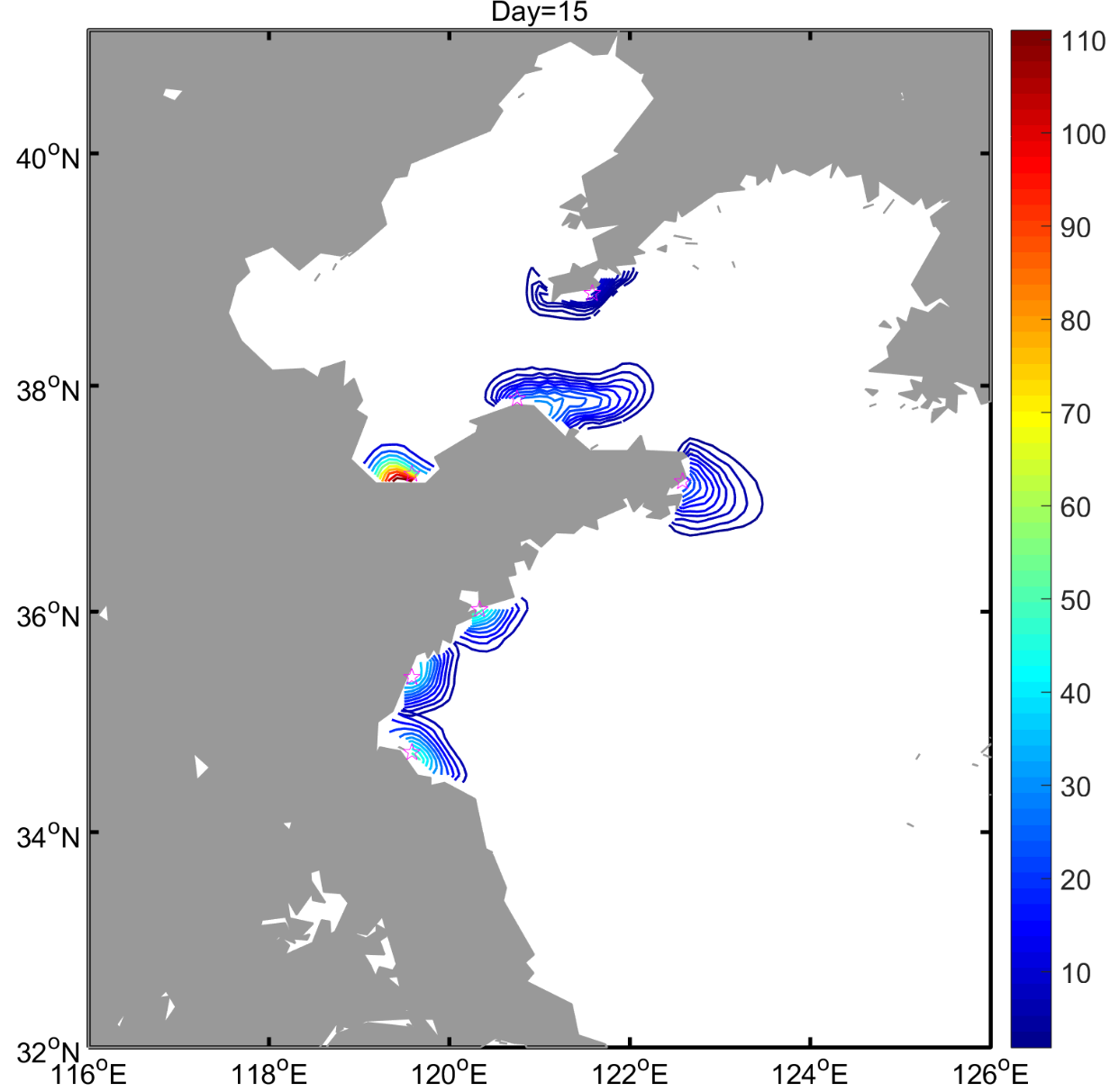

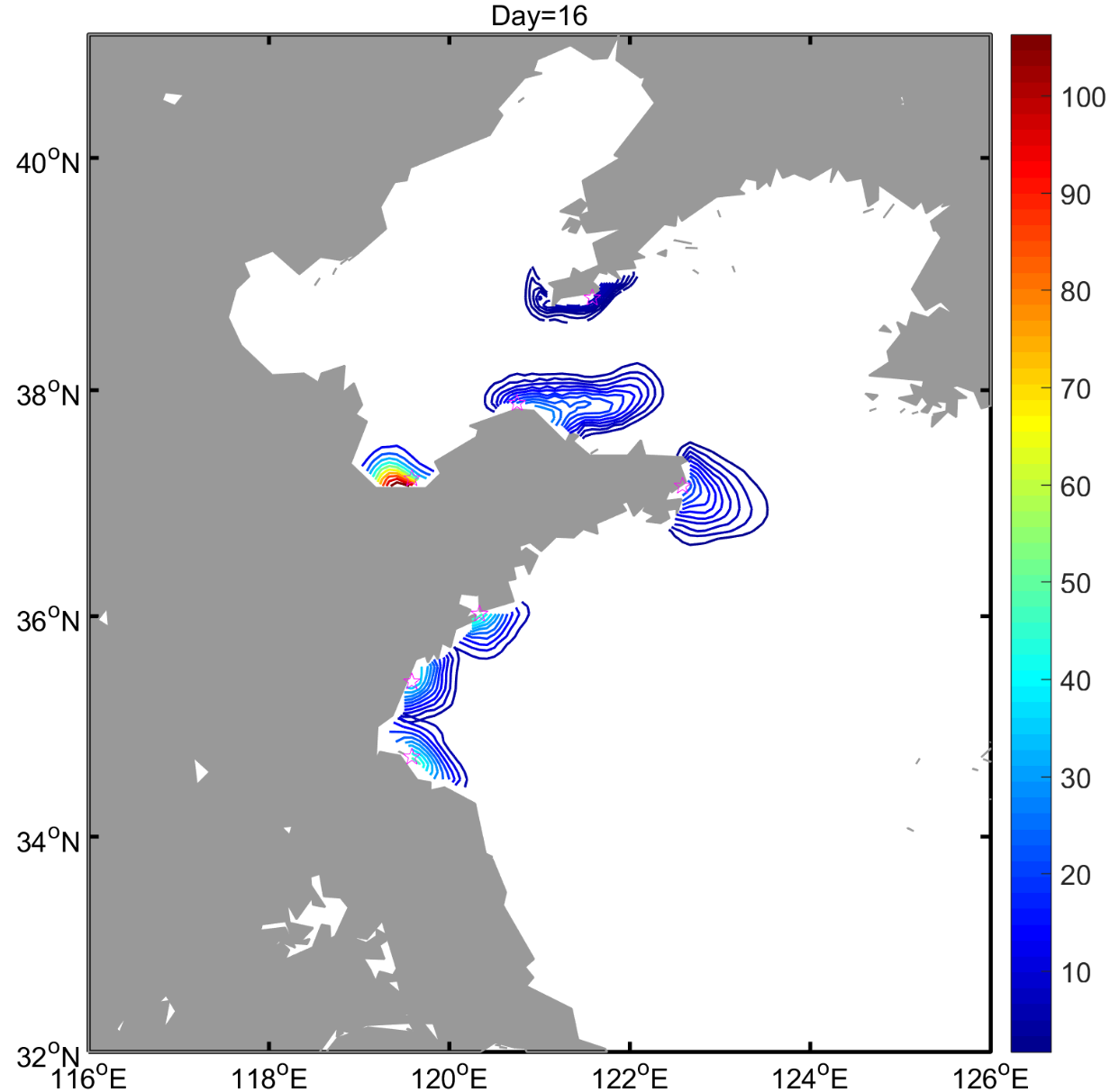

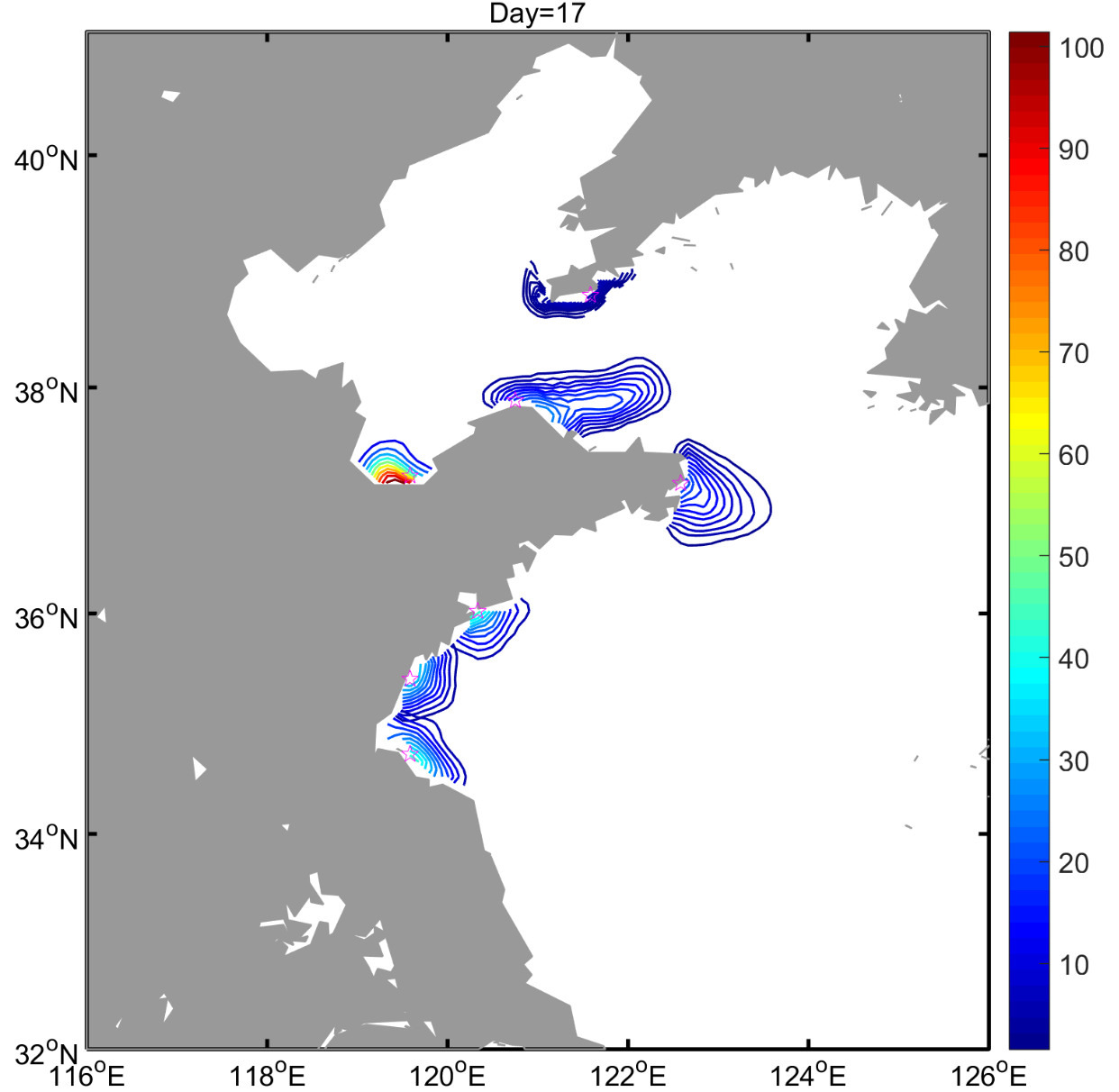

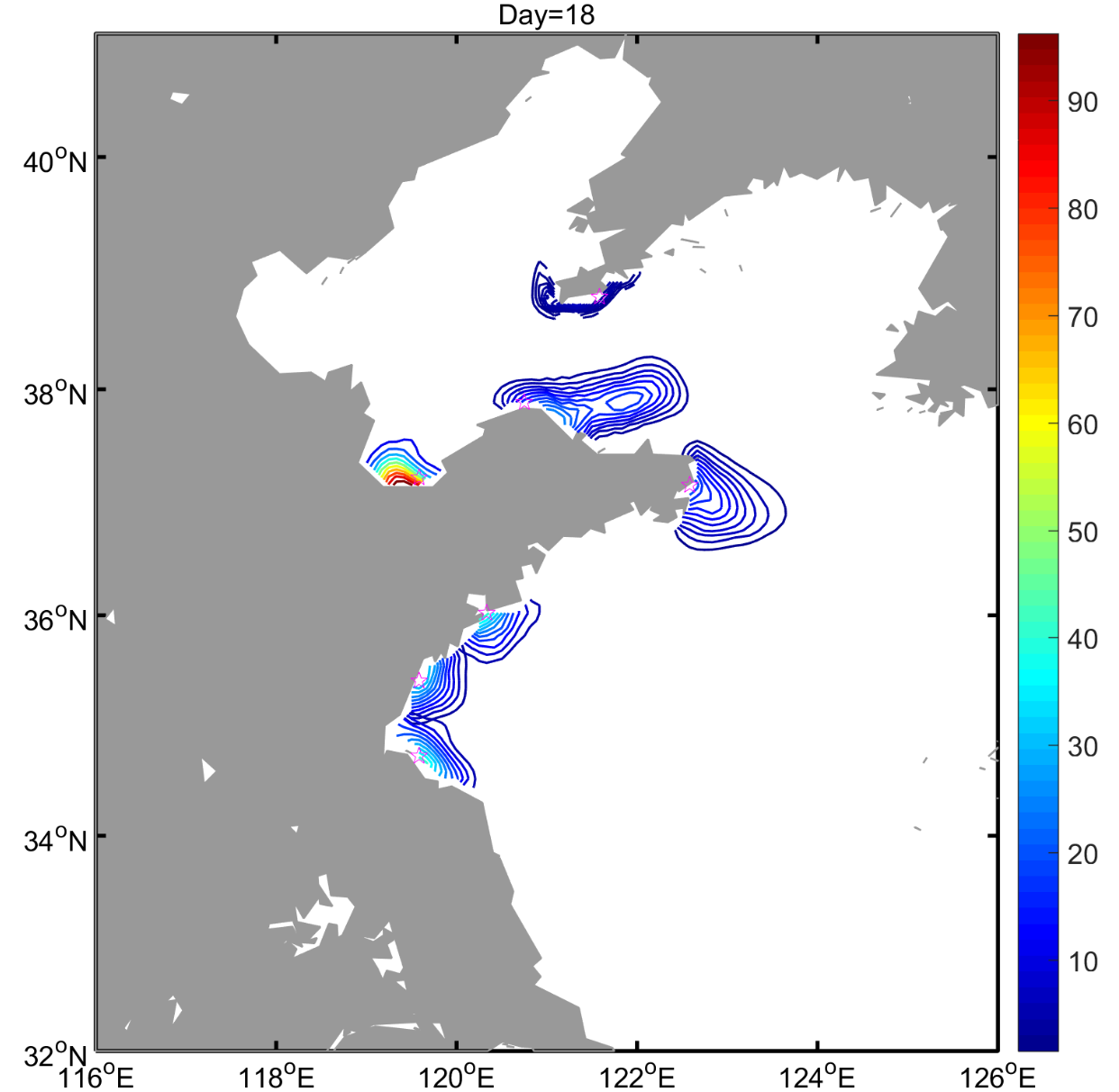

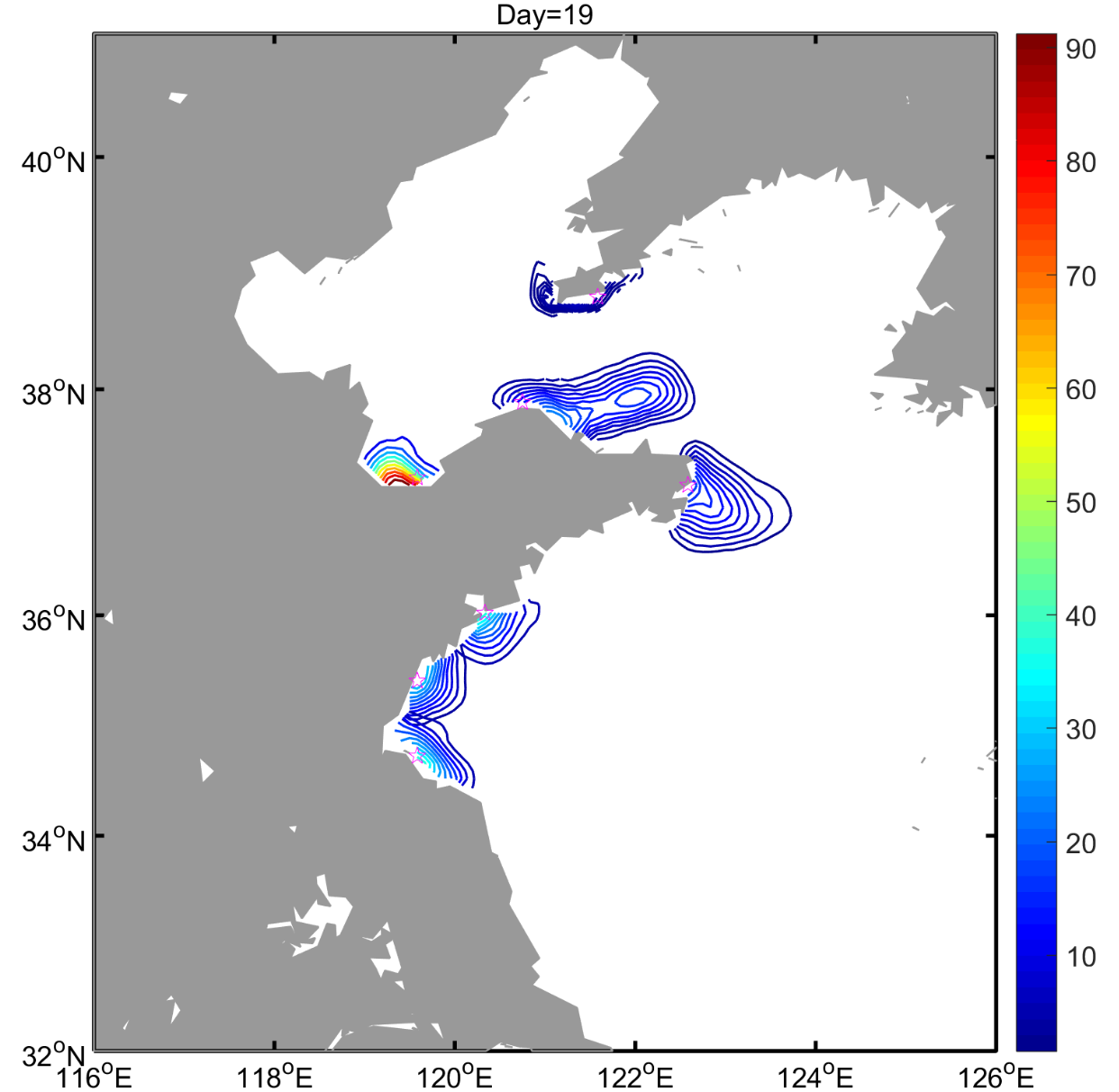

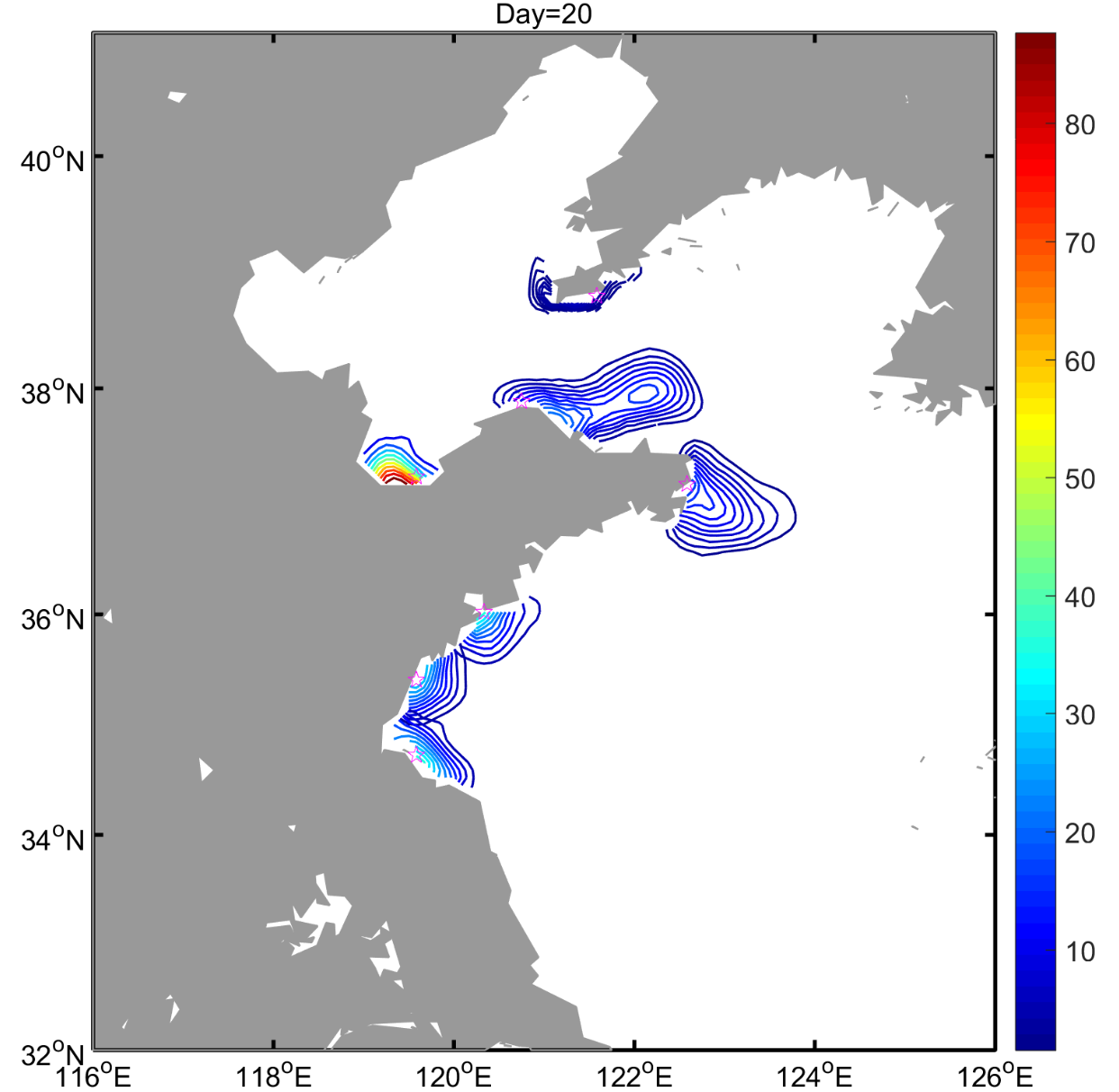

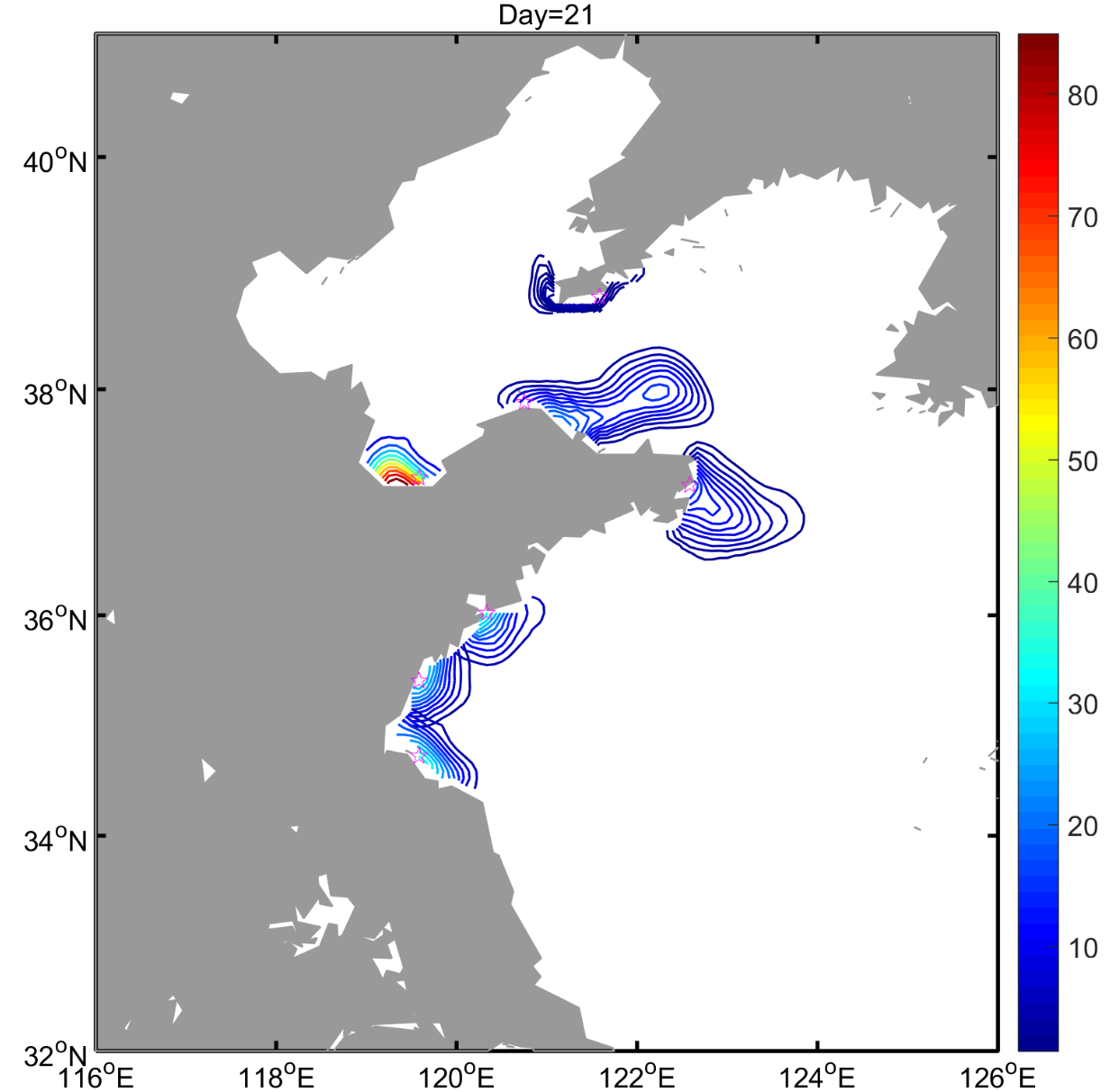

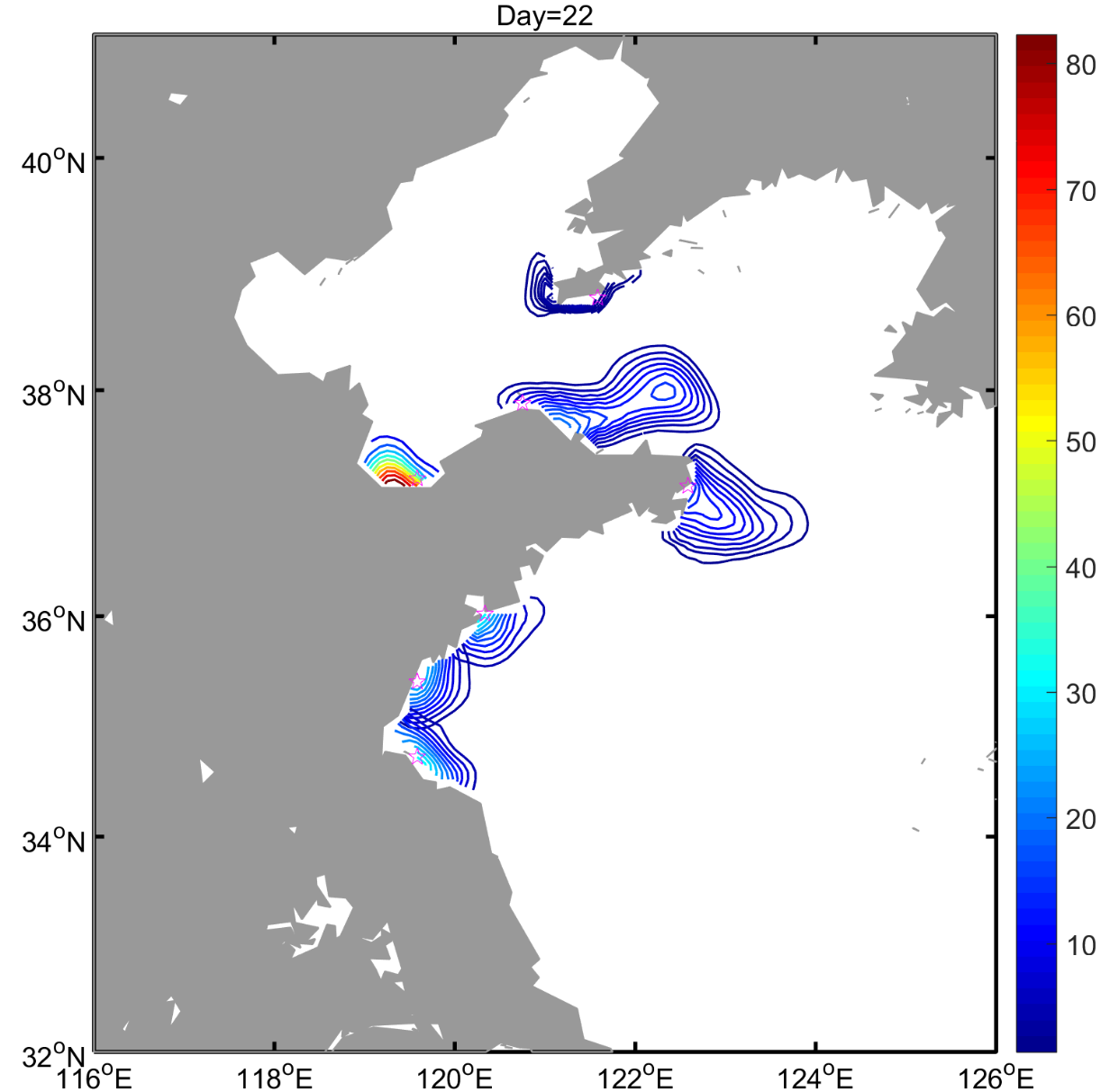

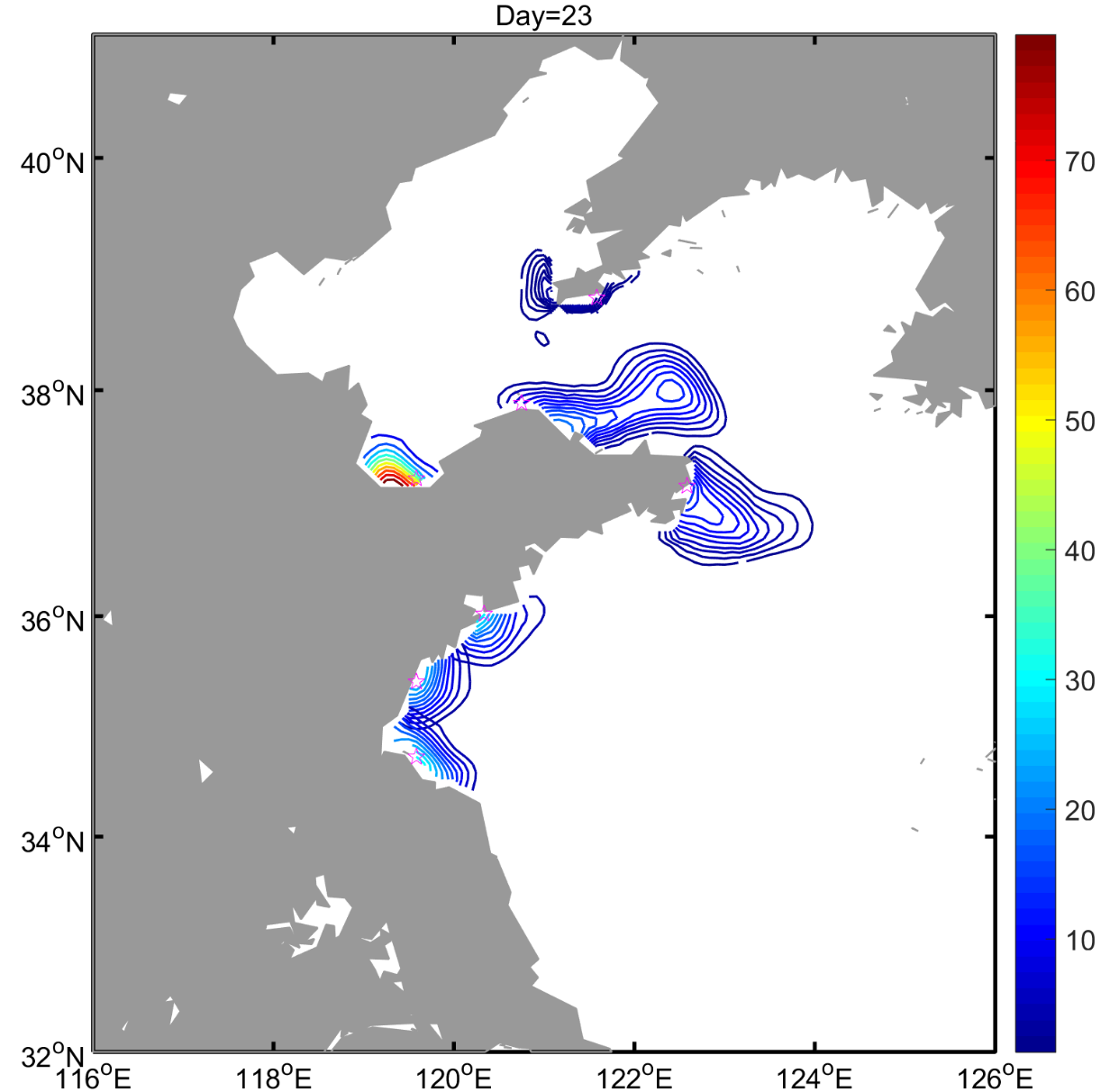

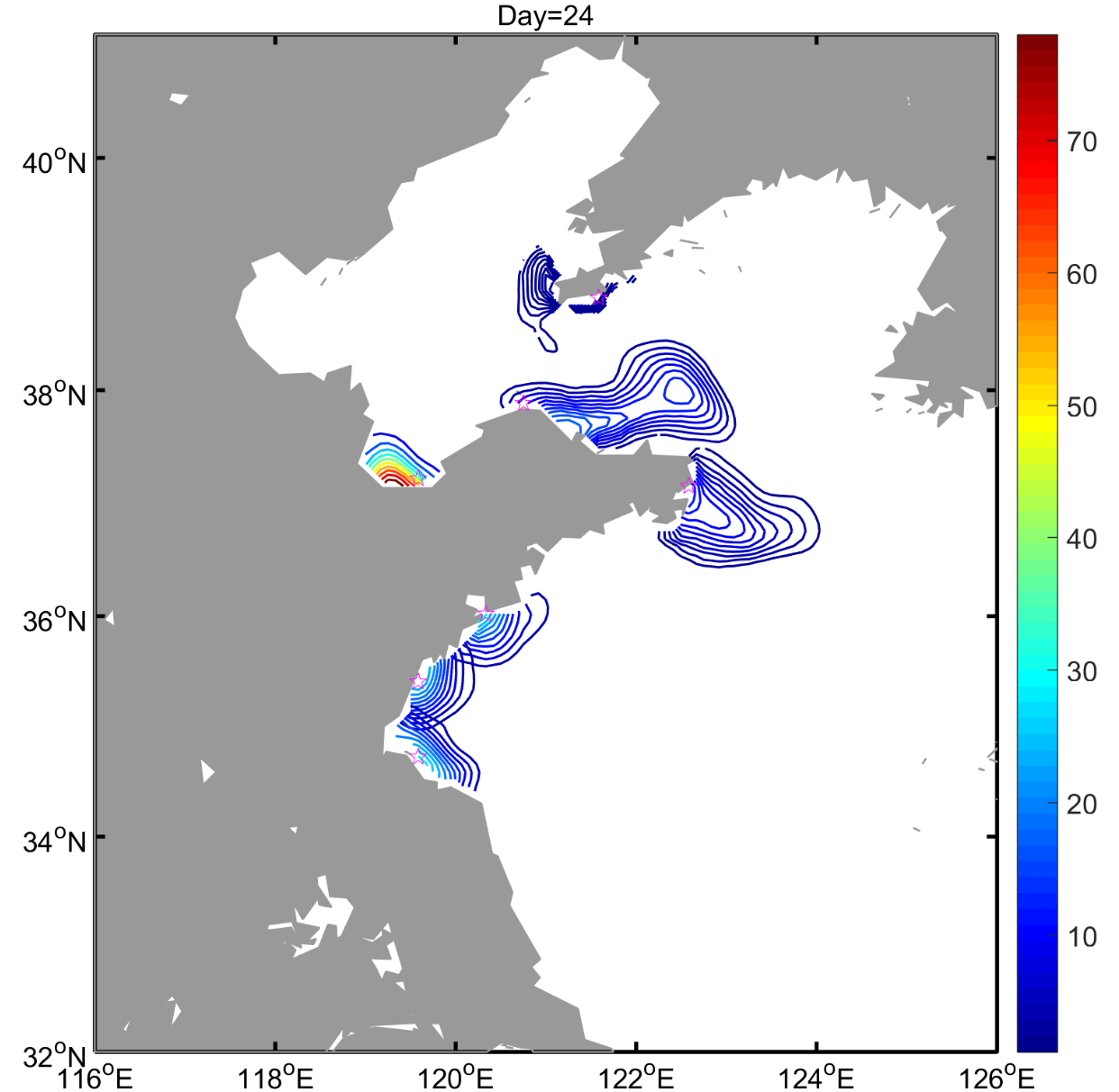

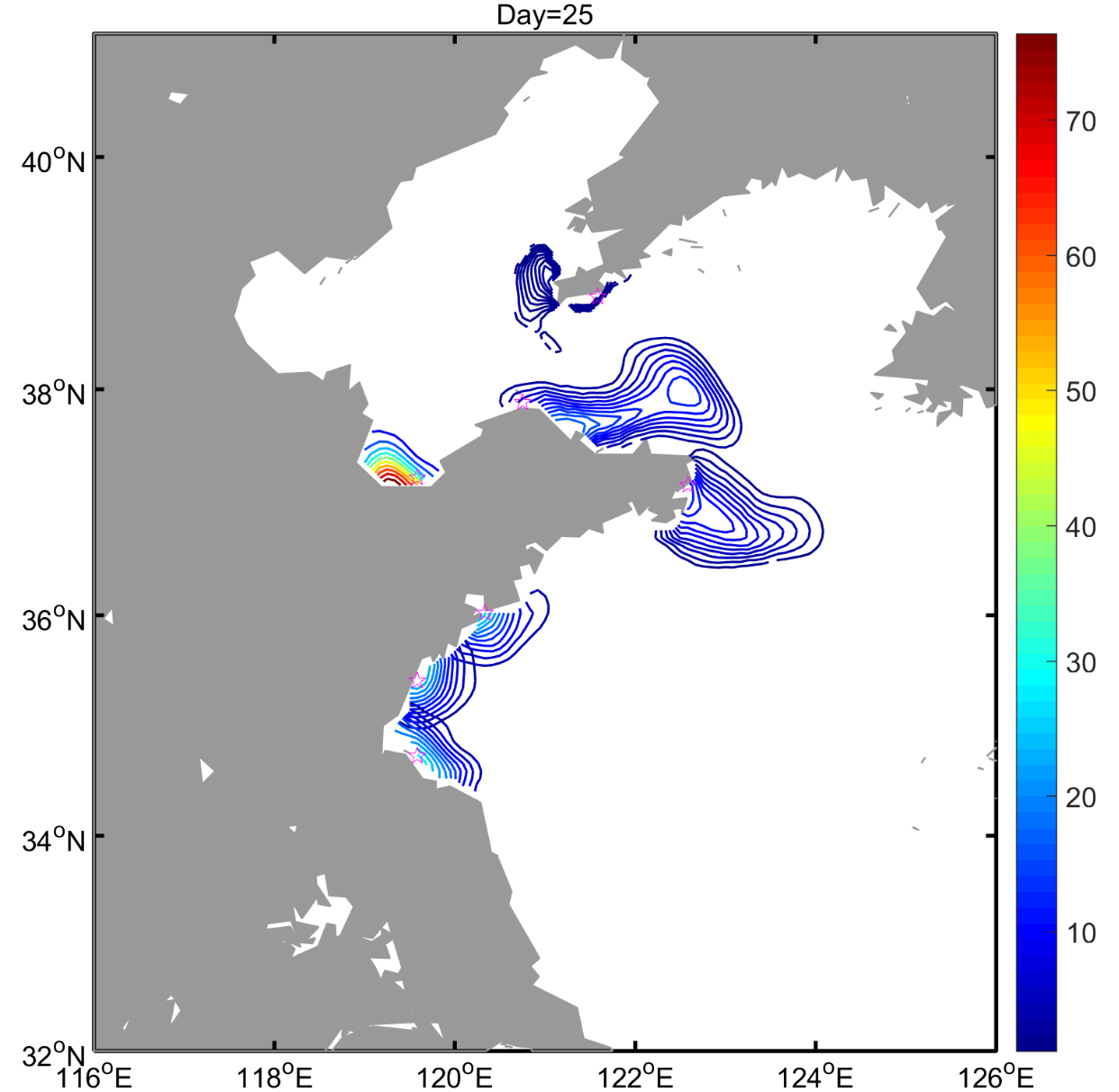

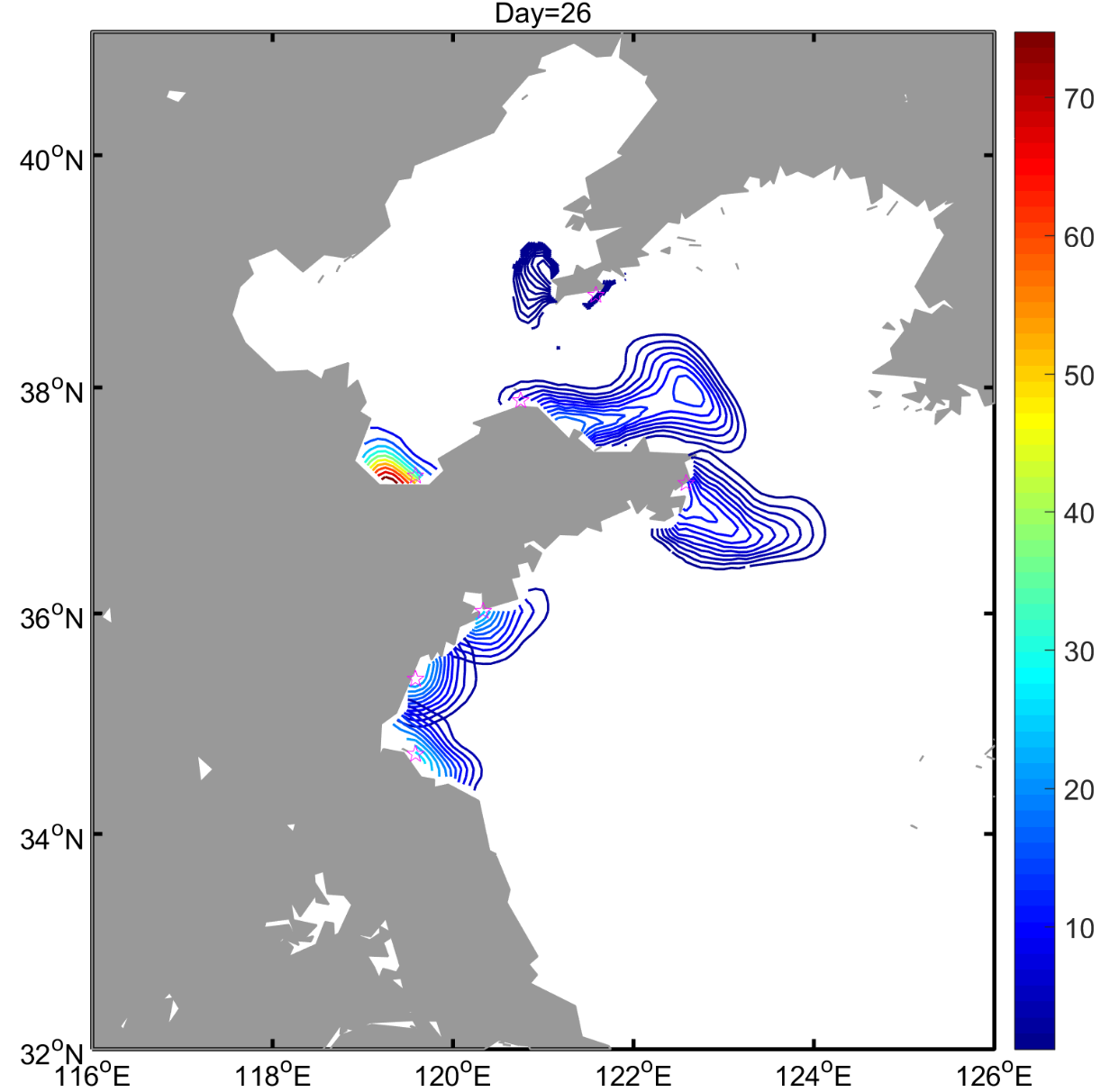

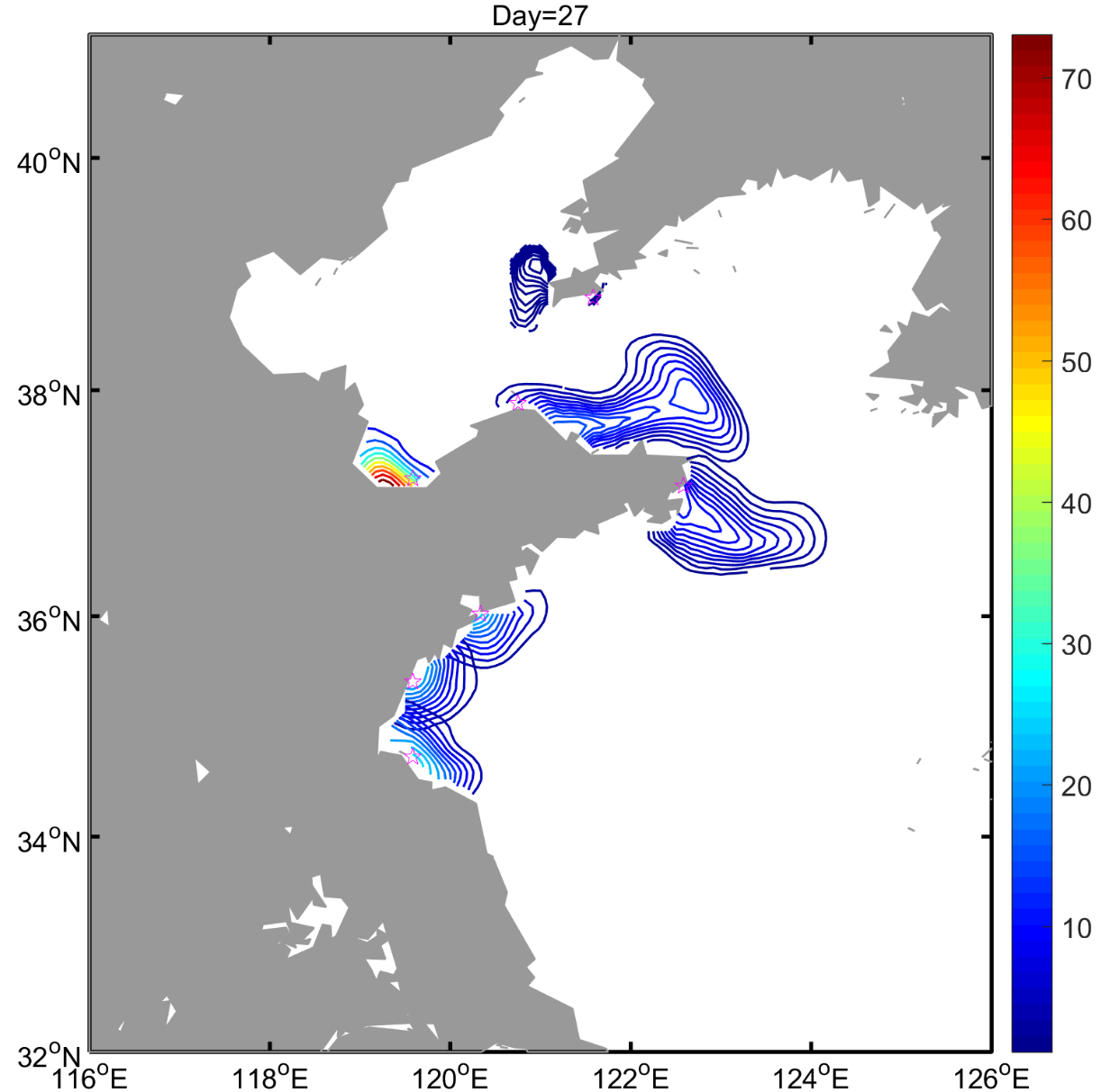

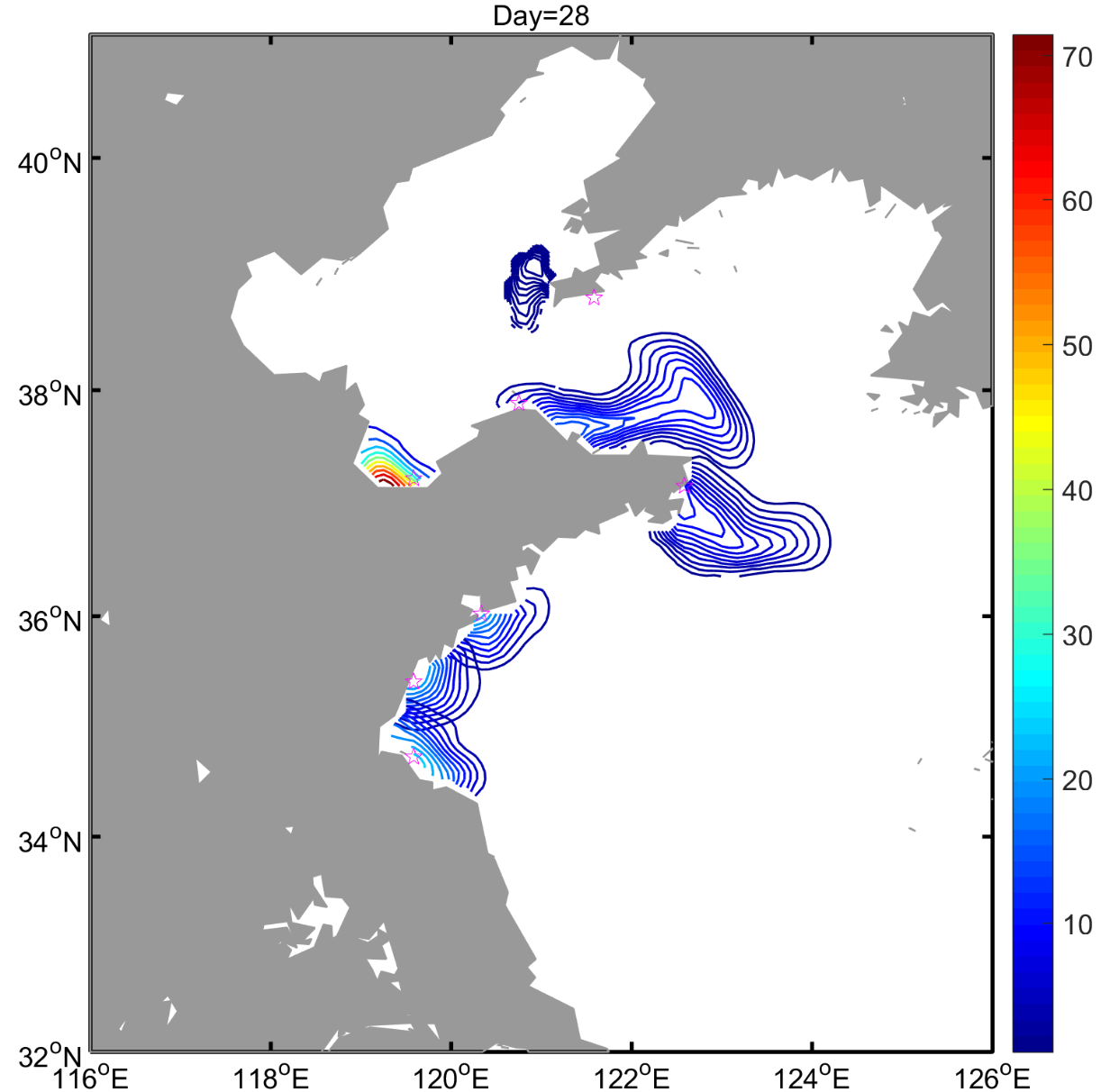

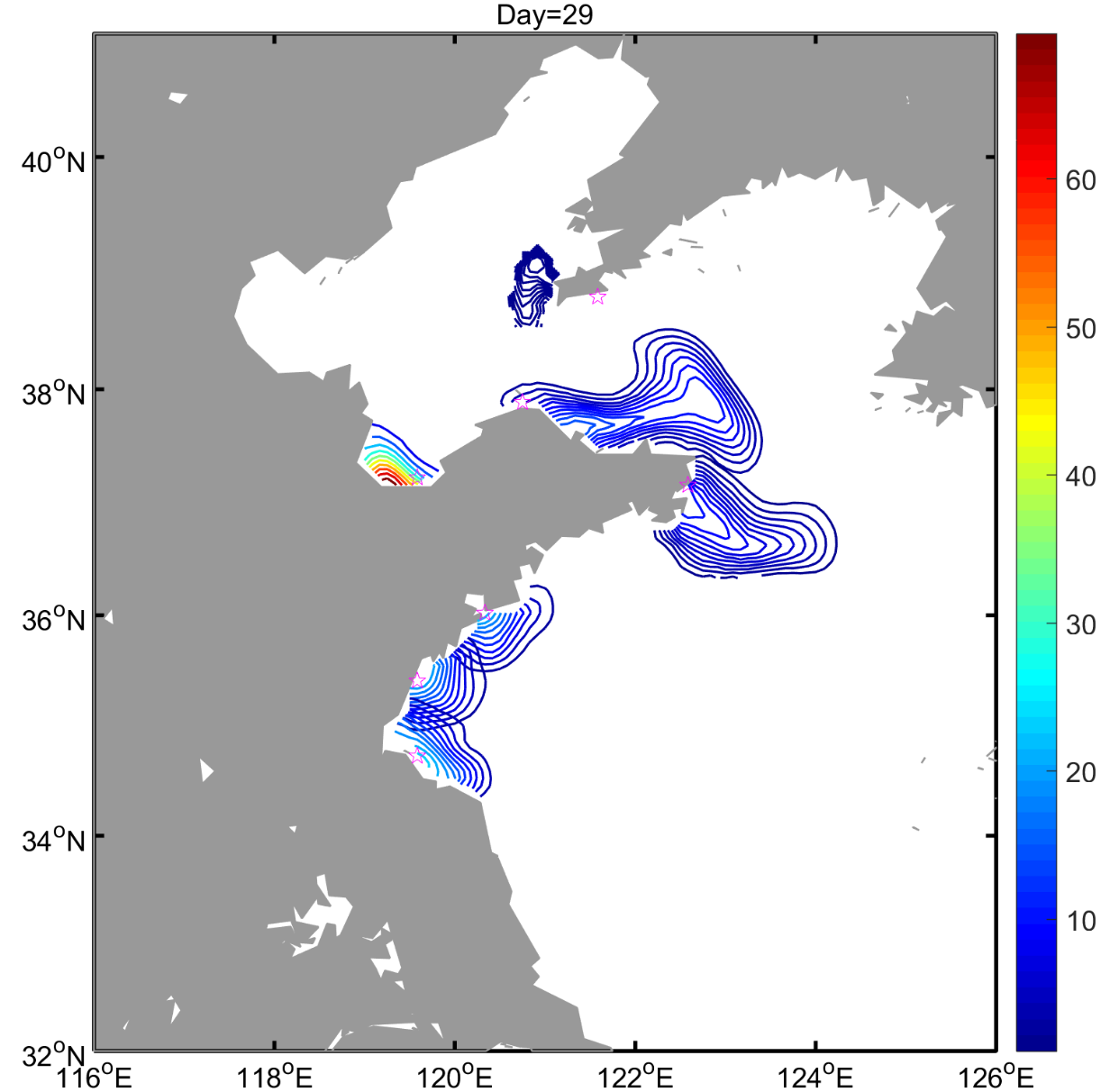

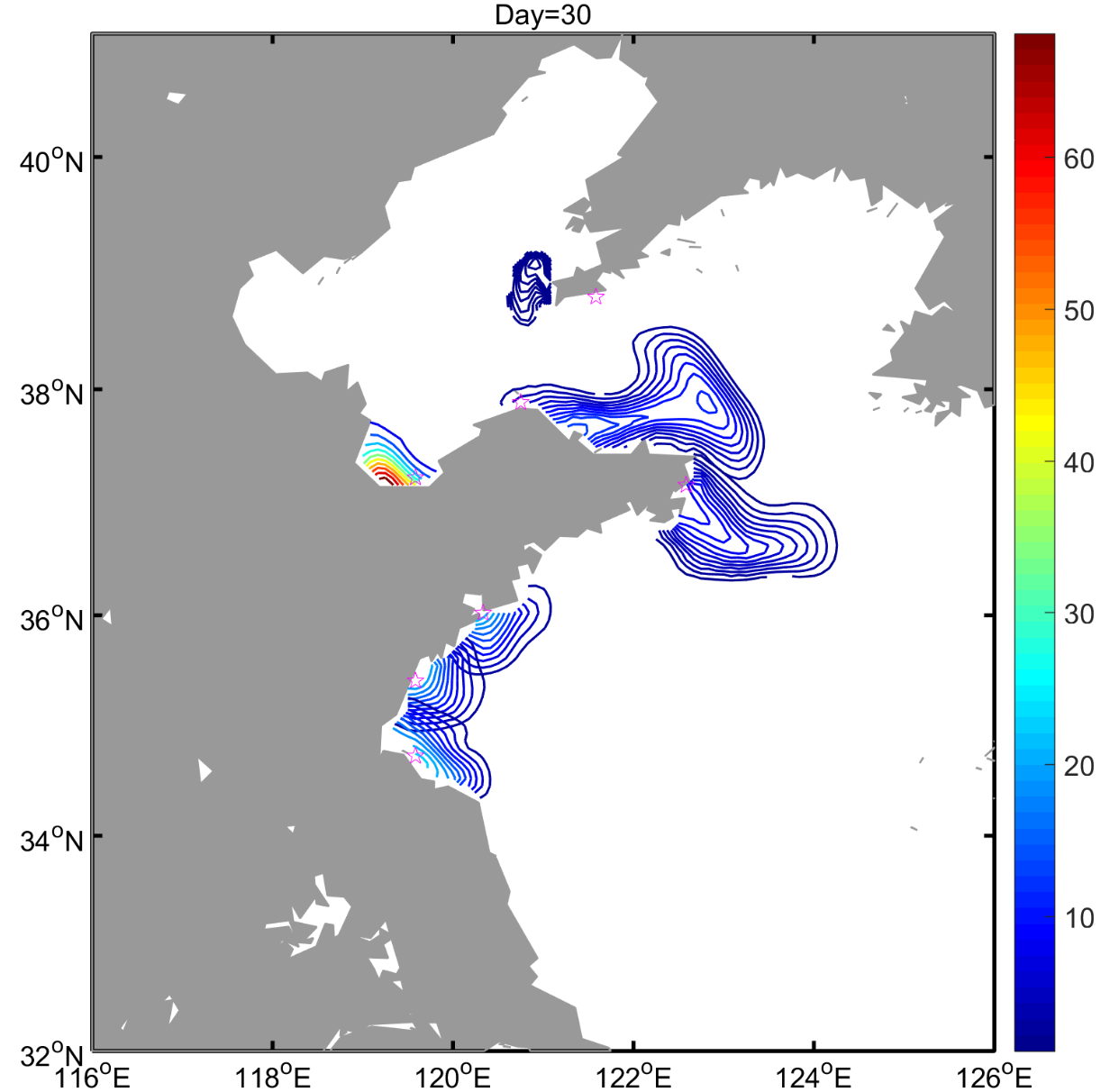


## **Figure S9** Dispersal path and relative content of particles originating from seven distinct localities within the Northern populations were modeled using the OGCM for one month.


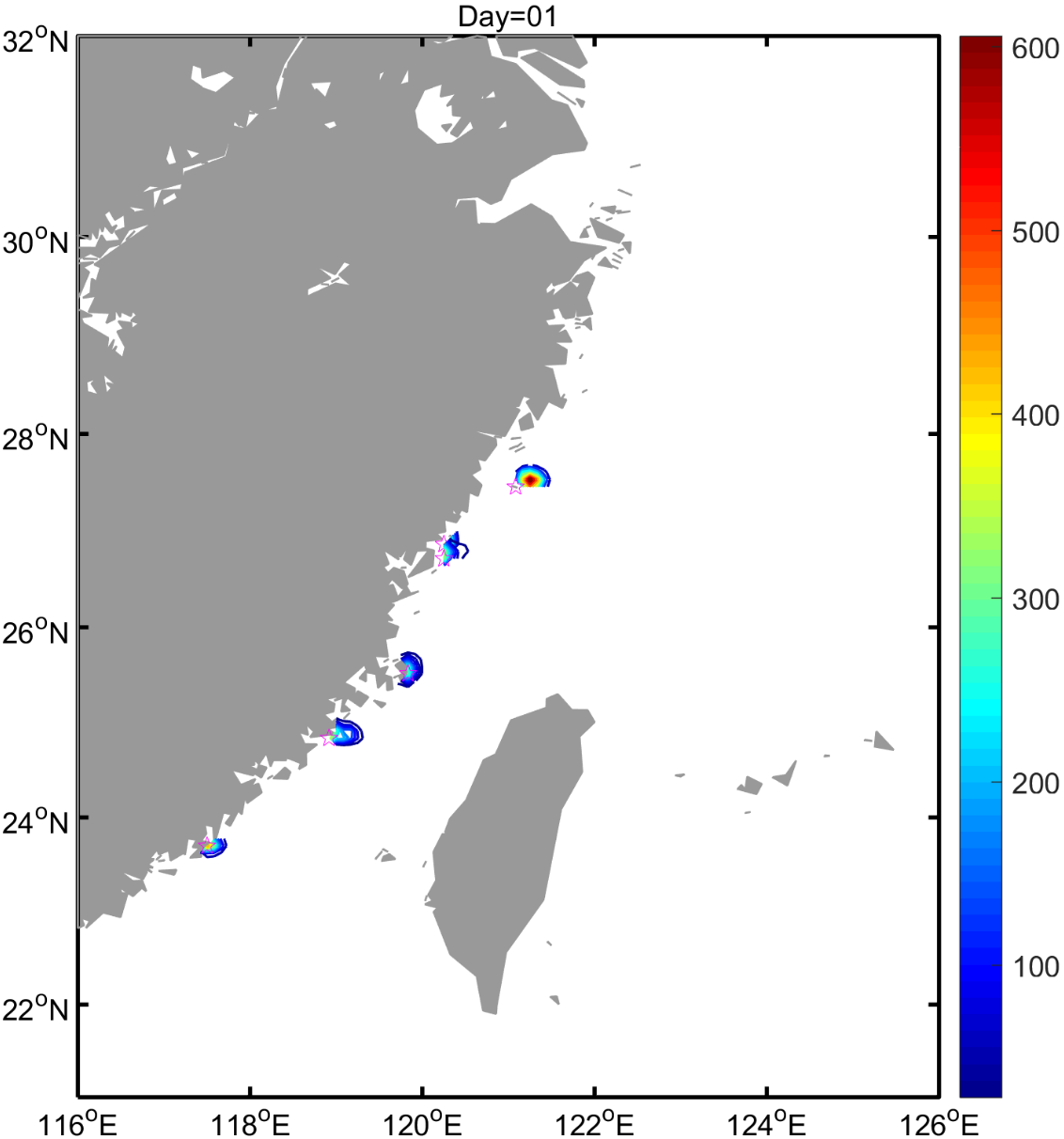

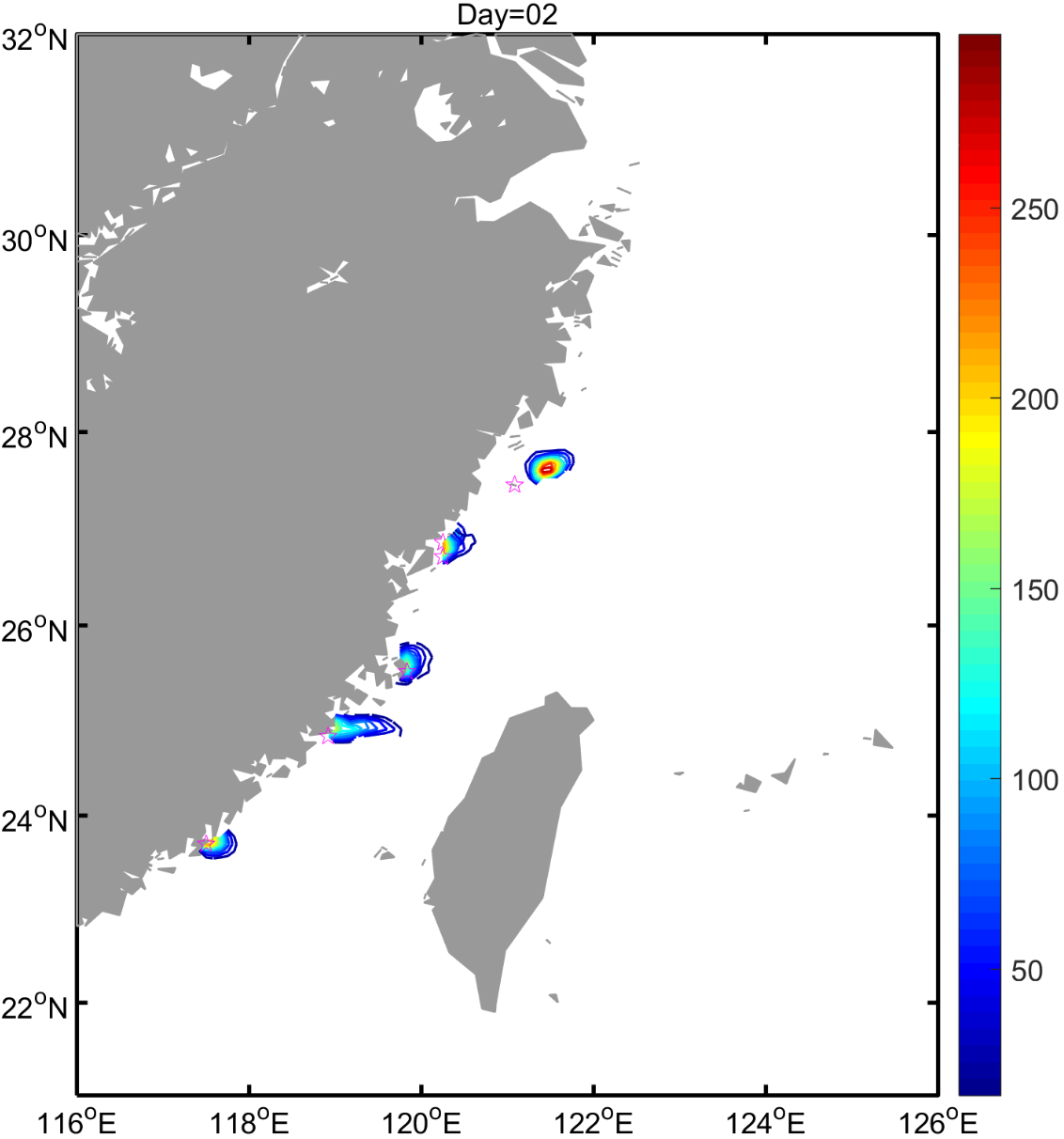

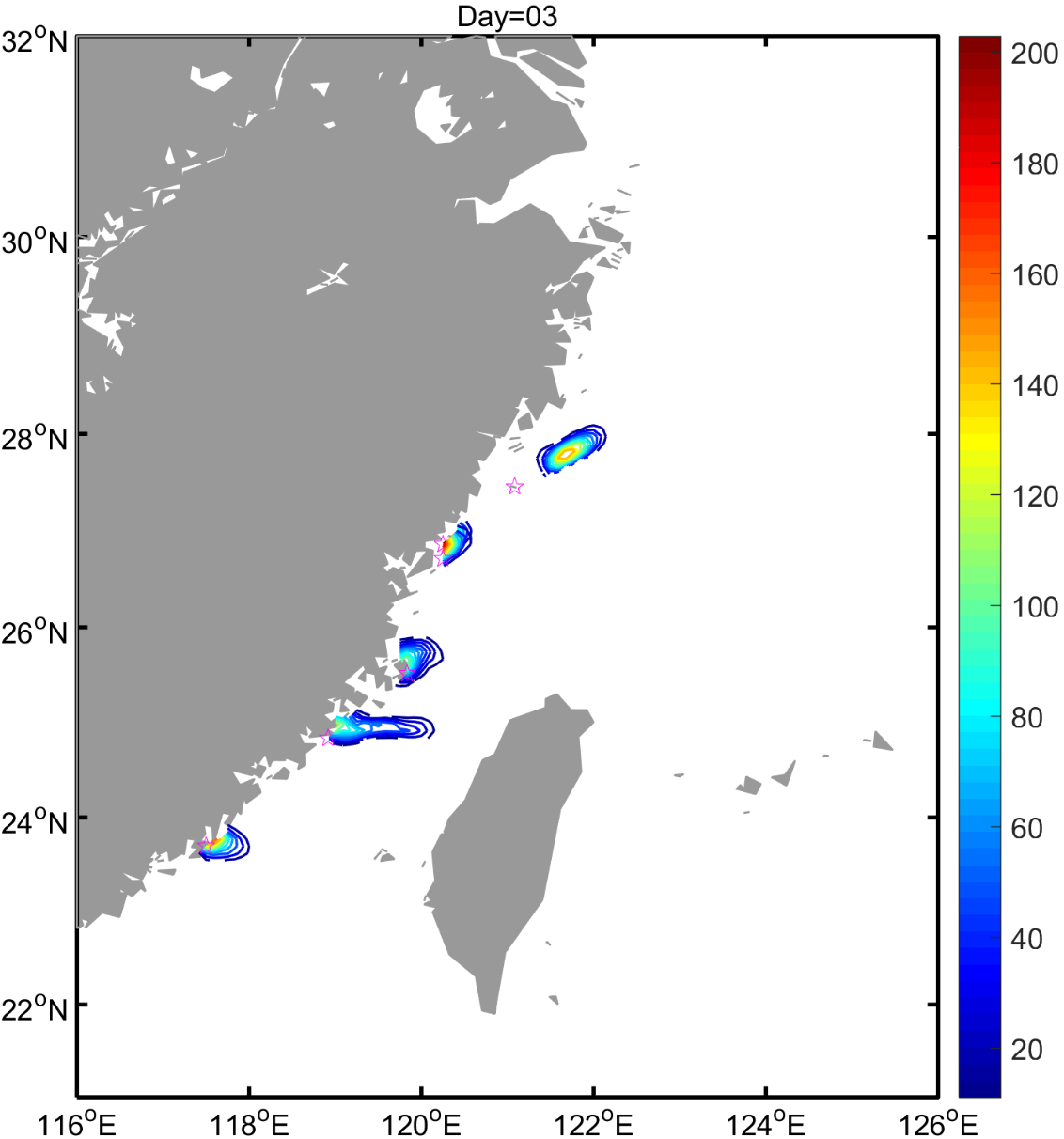

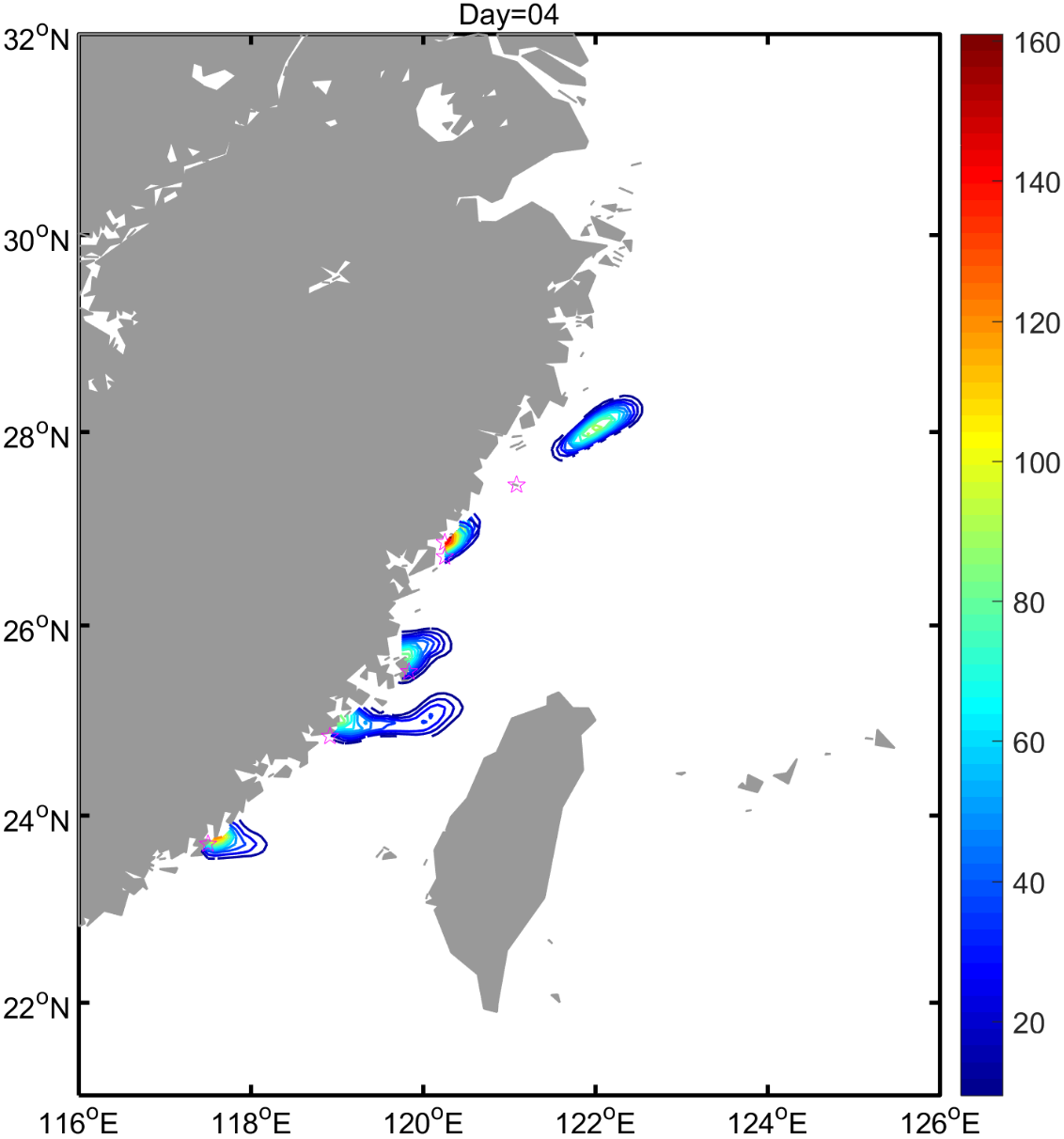

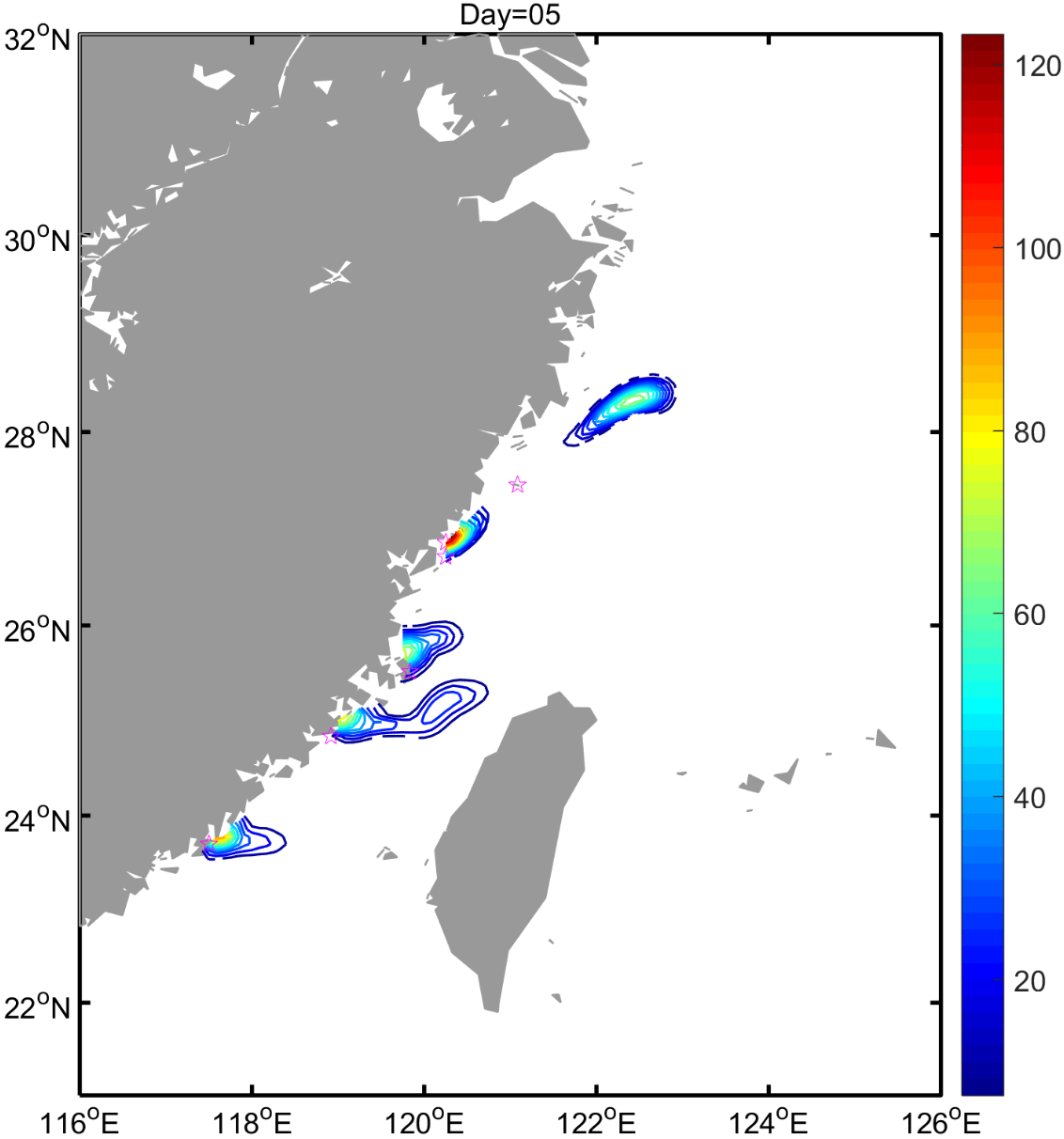

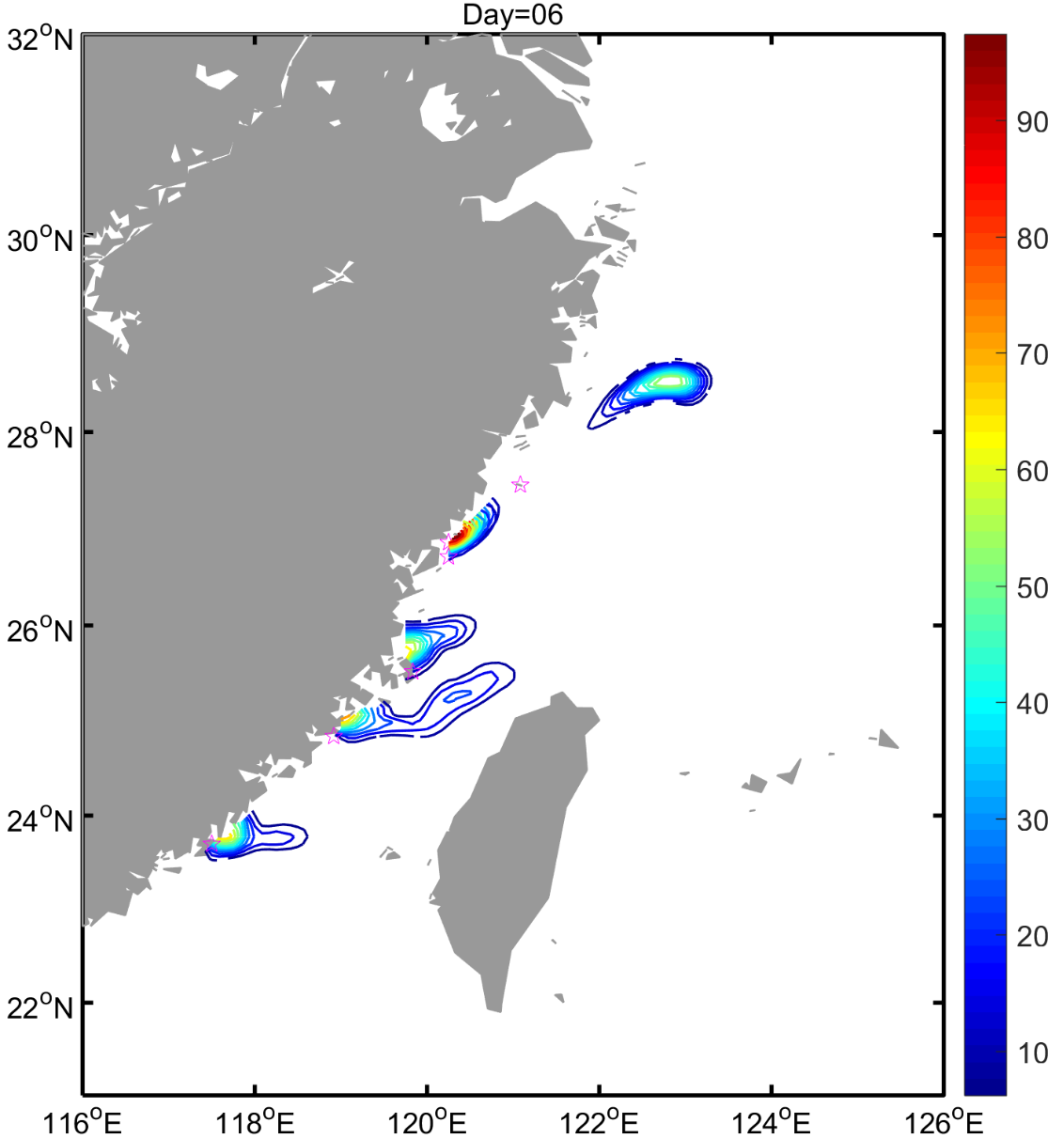

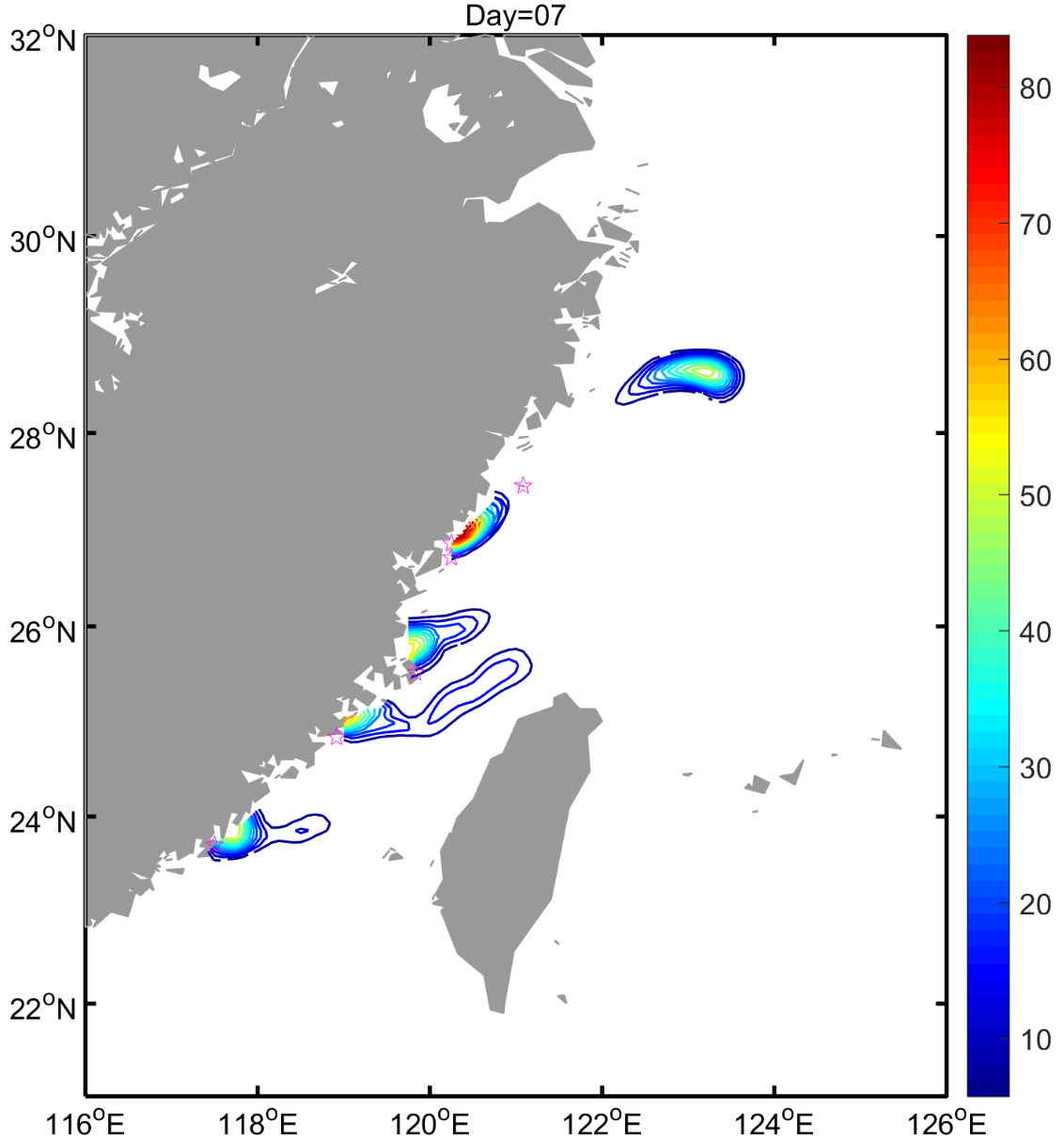

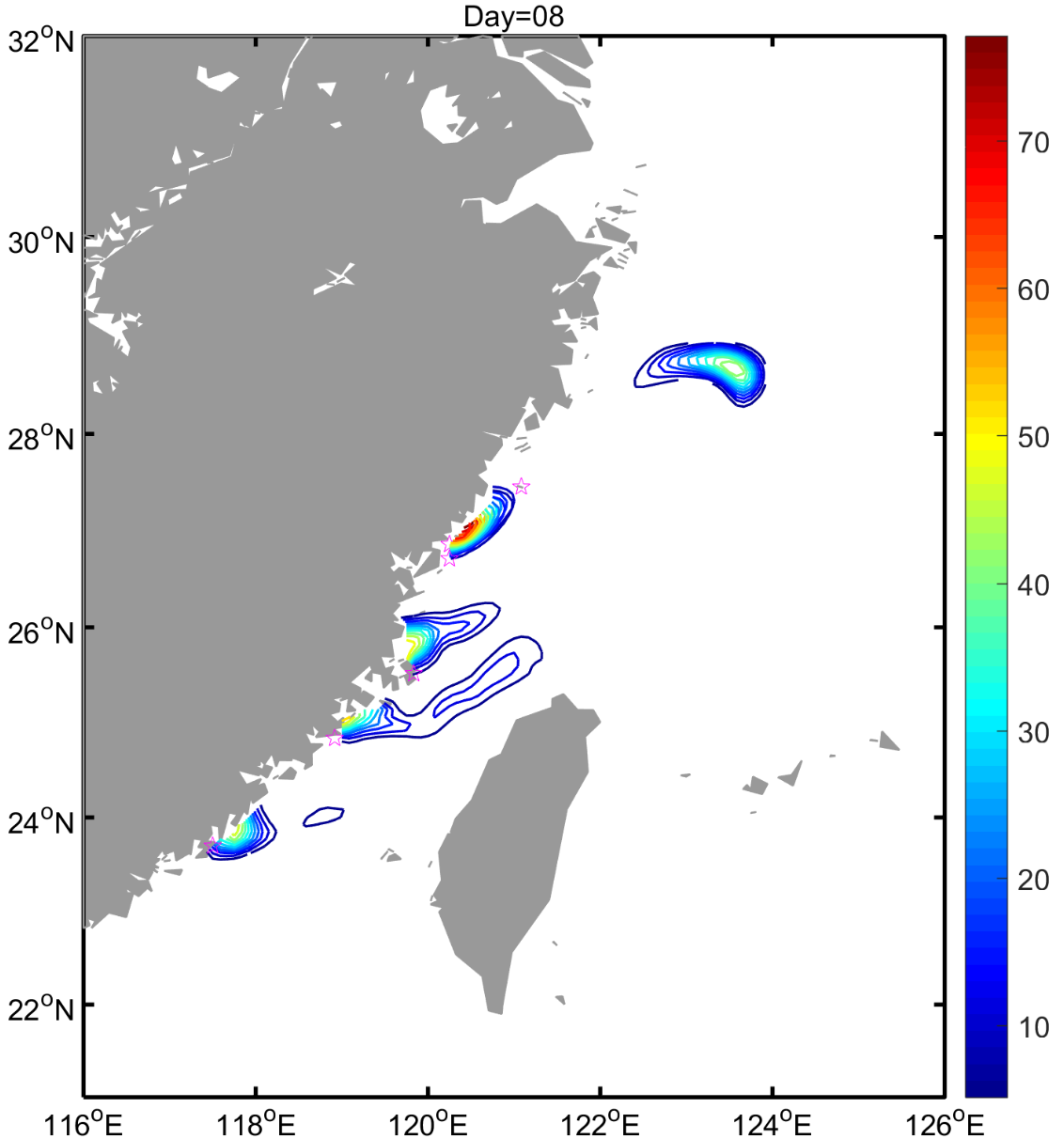

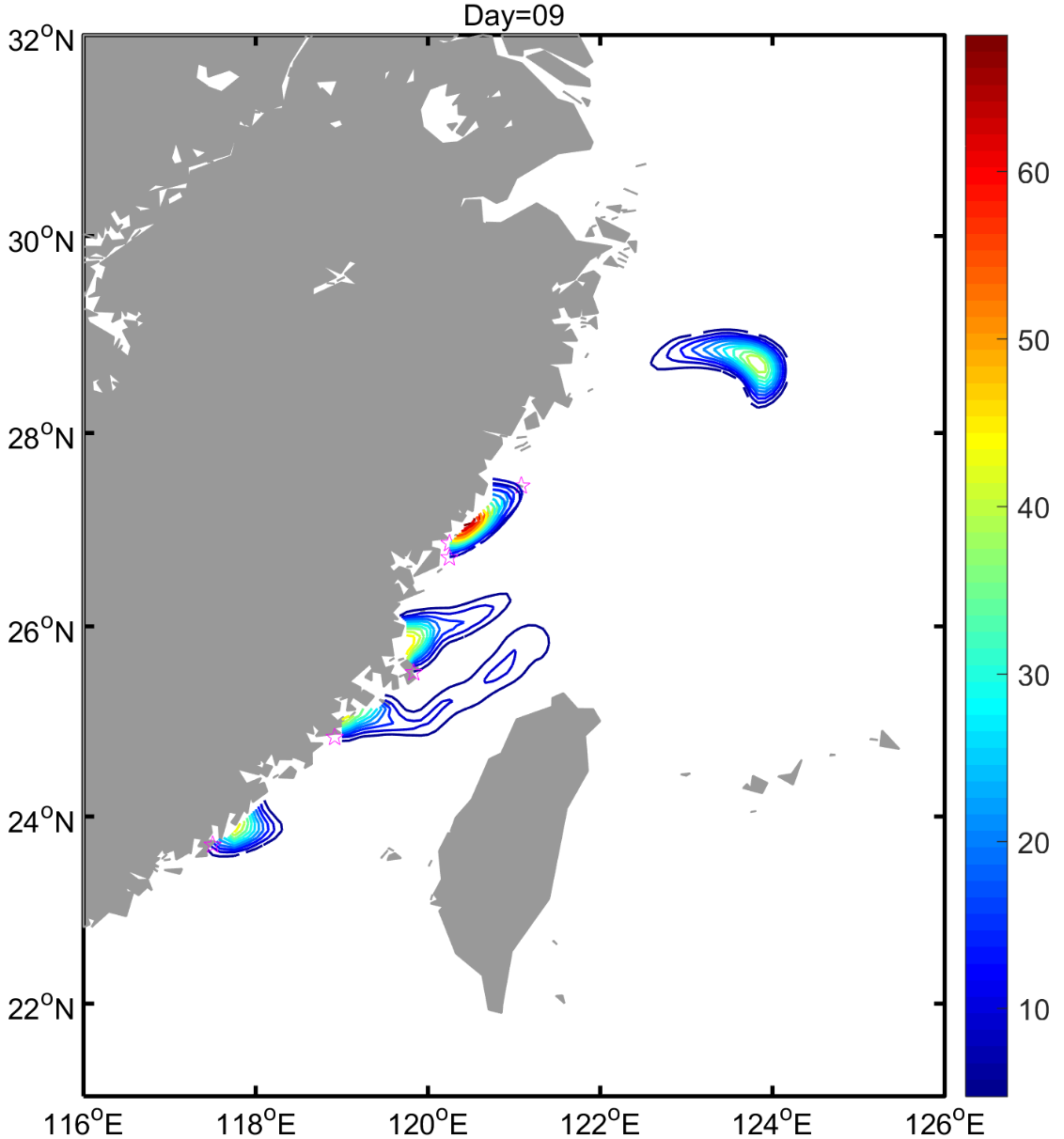

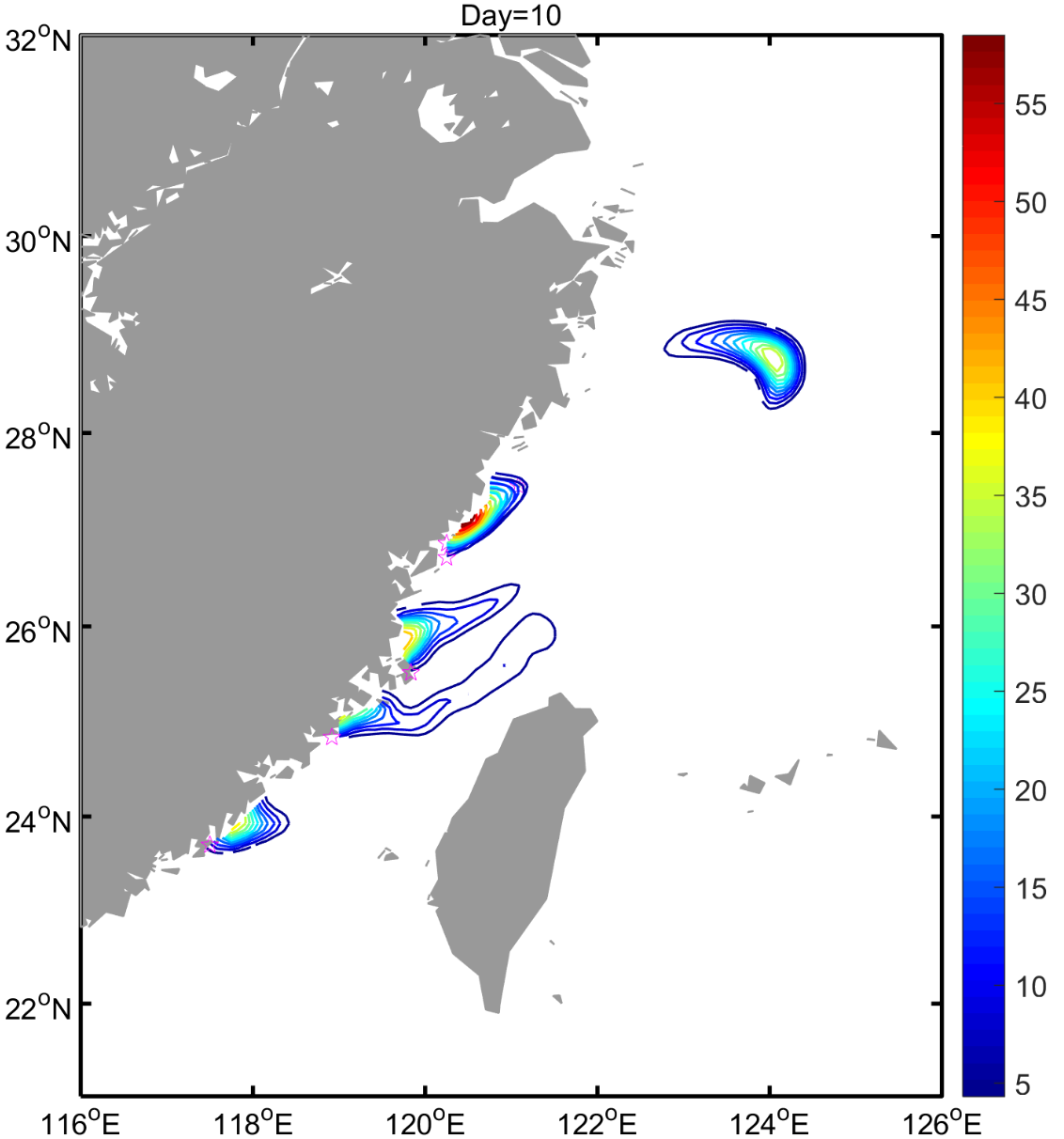

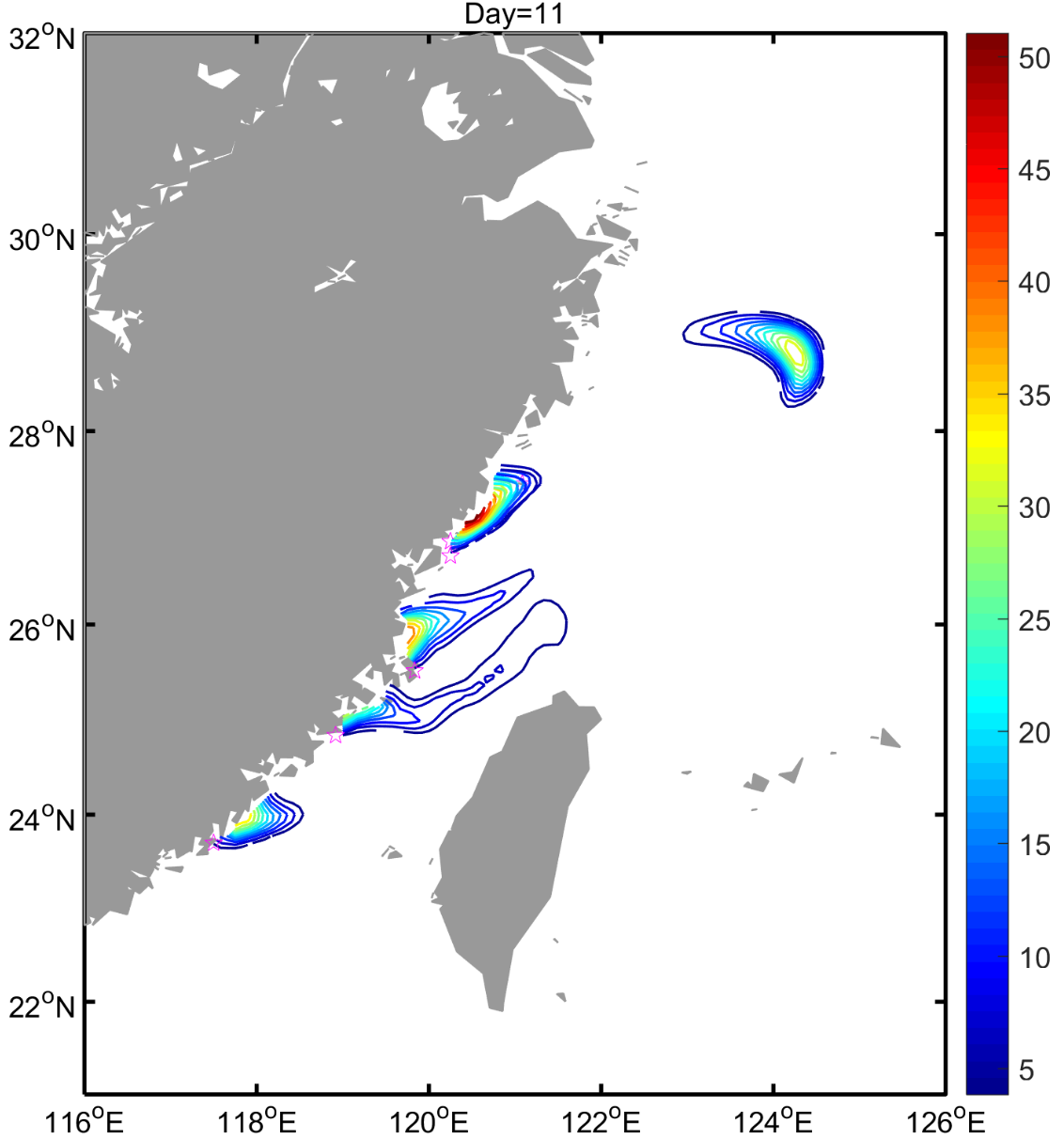

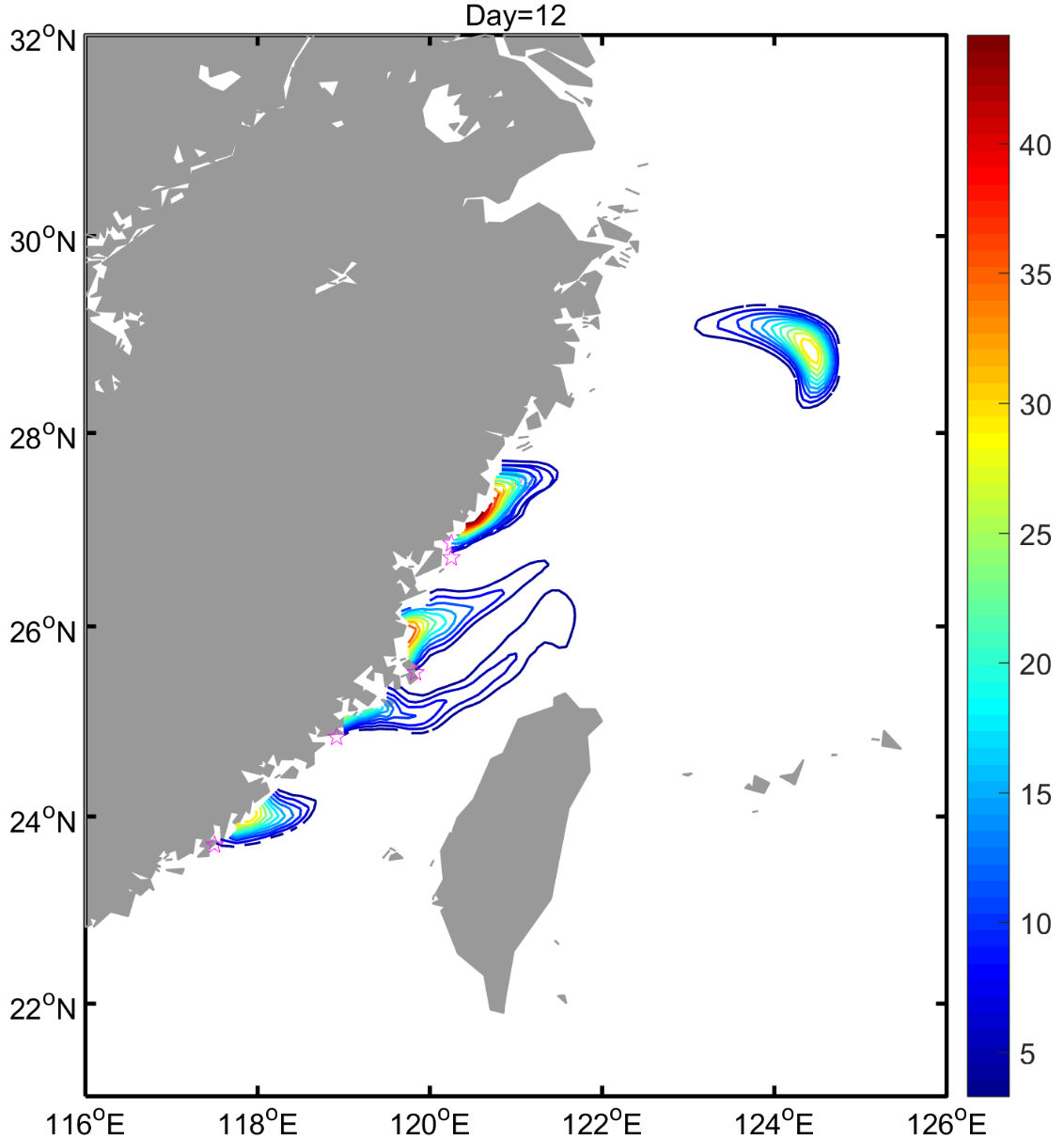

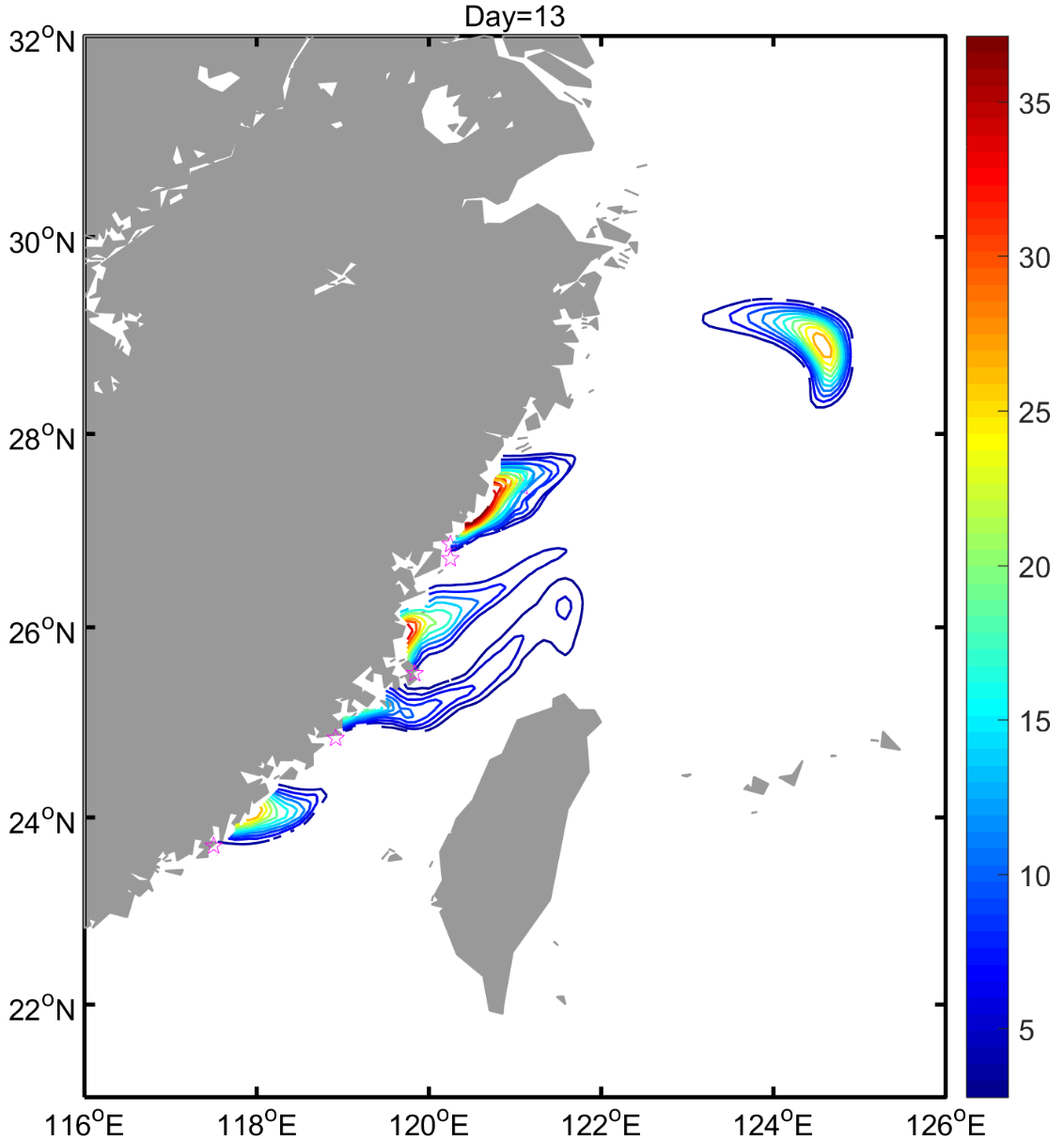

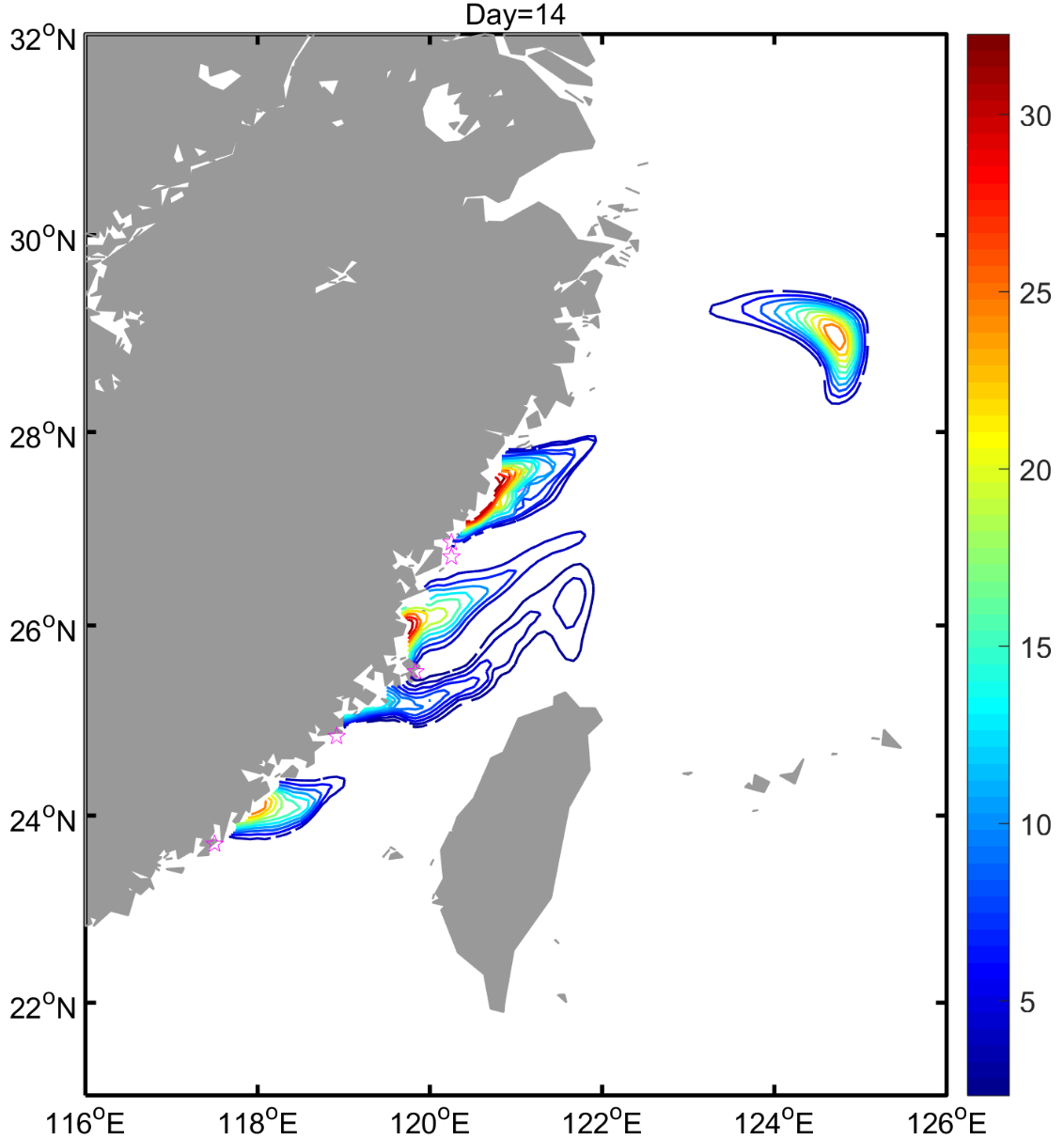

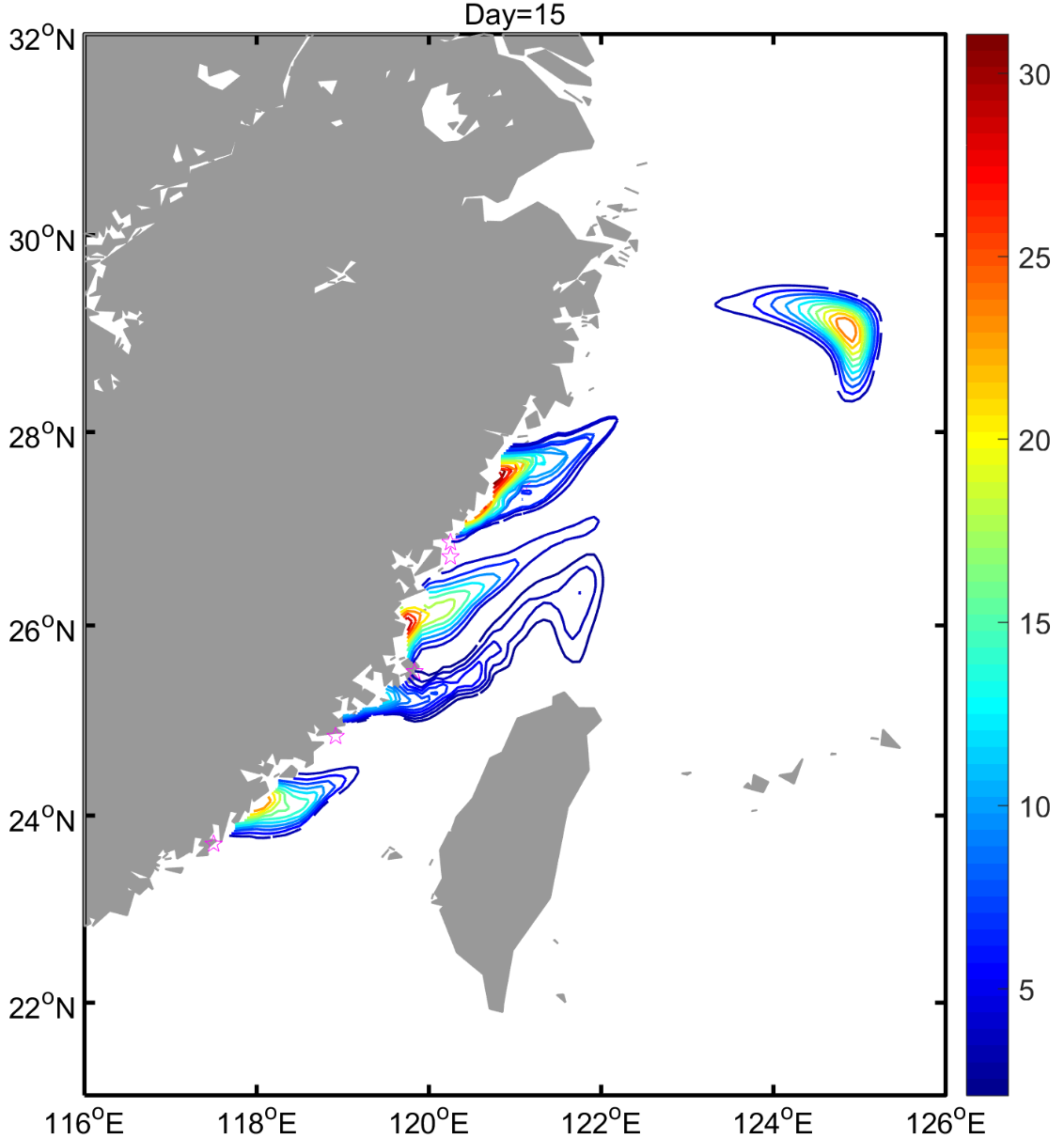

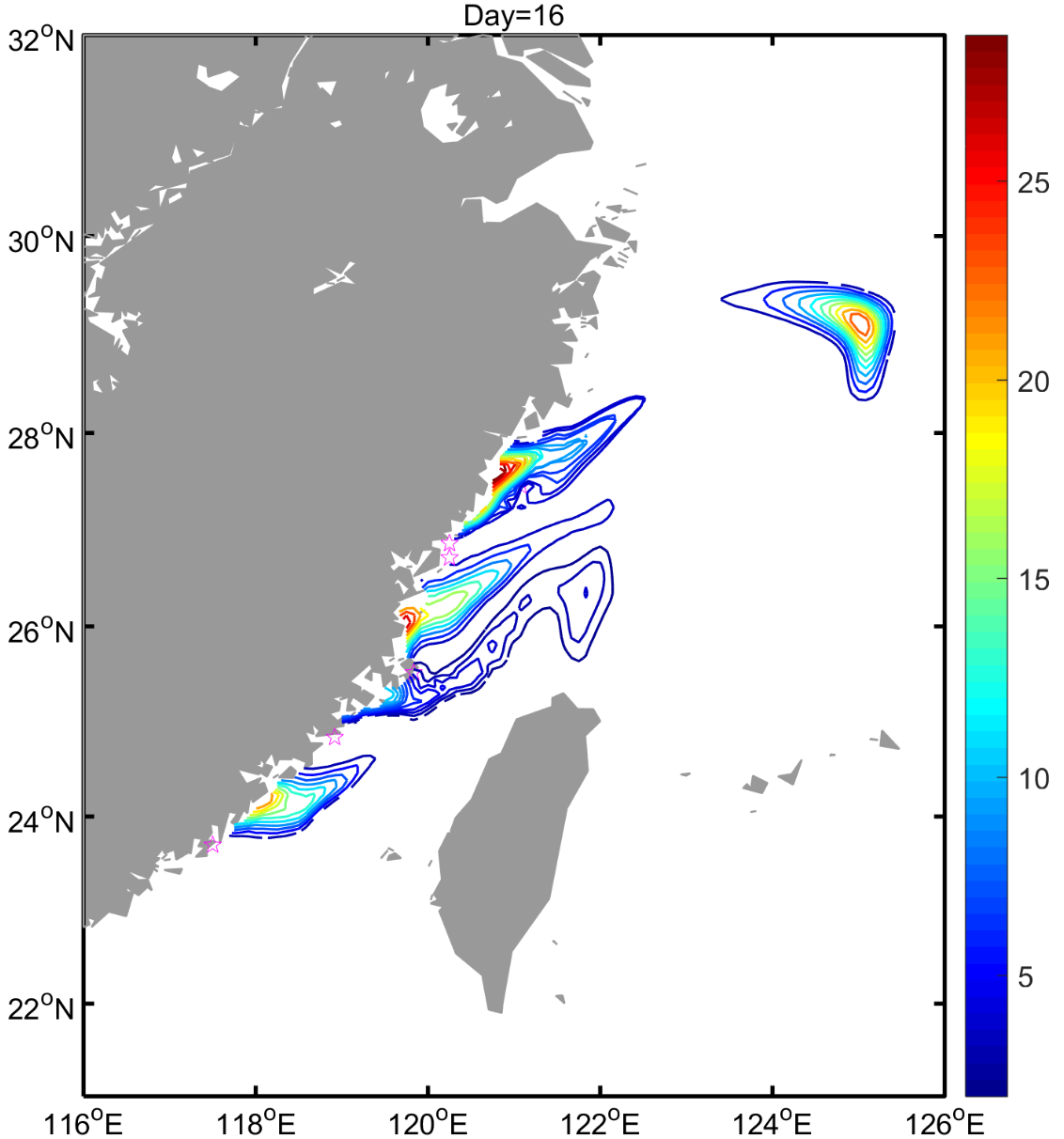

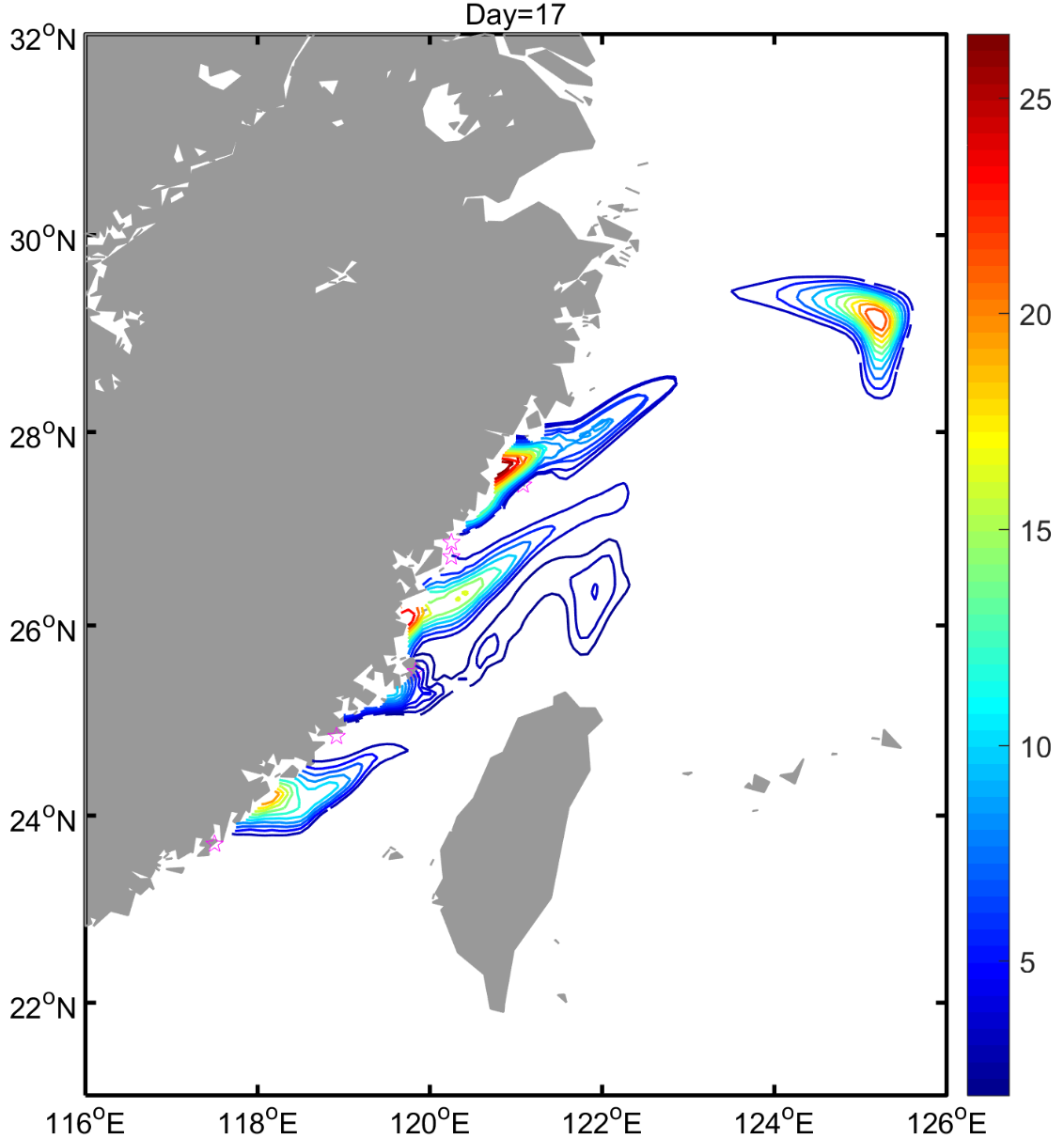

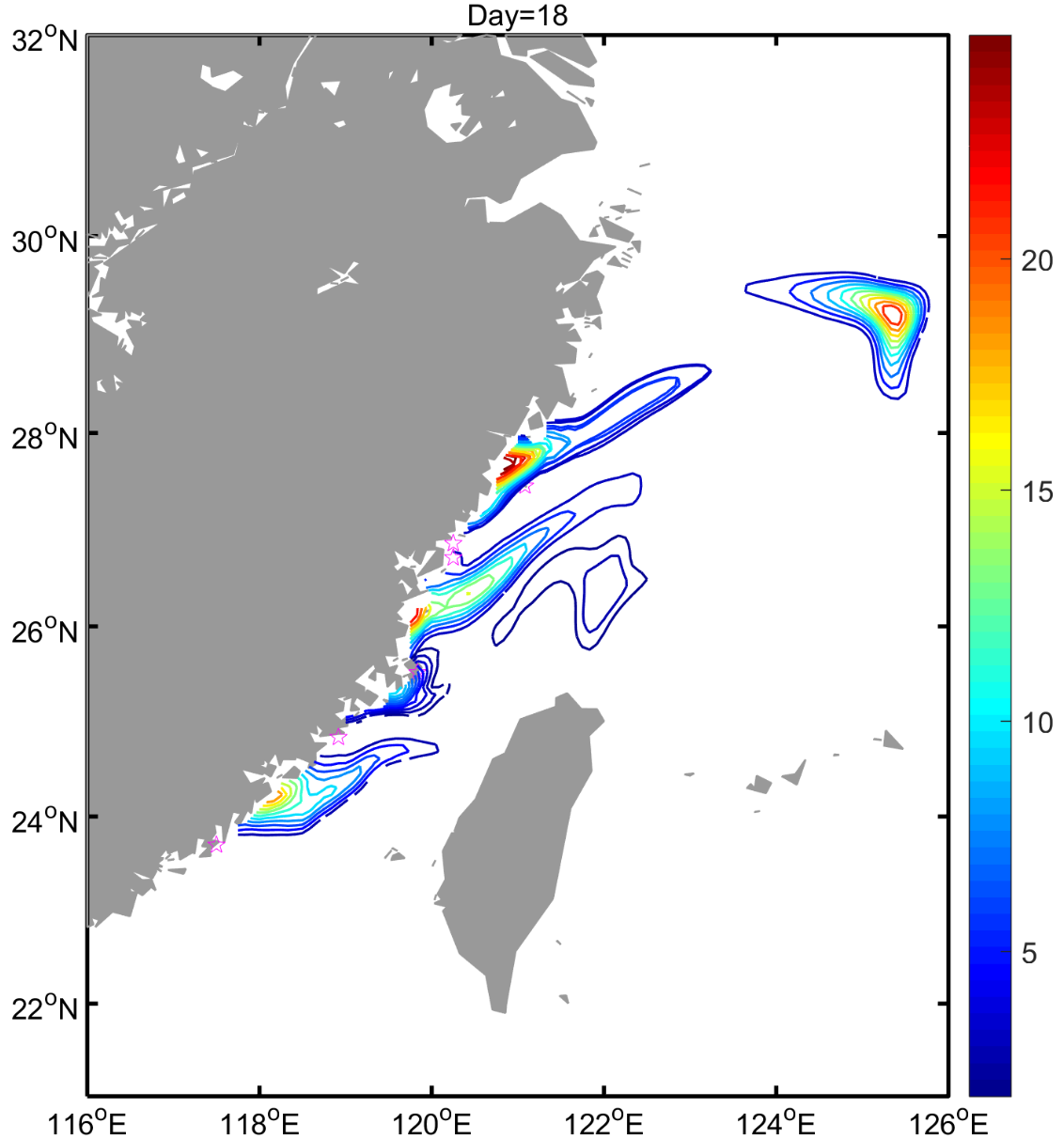

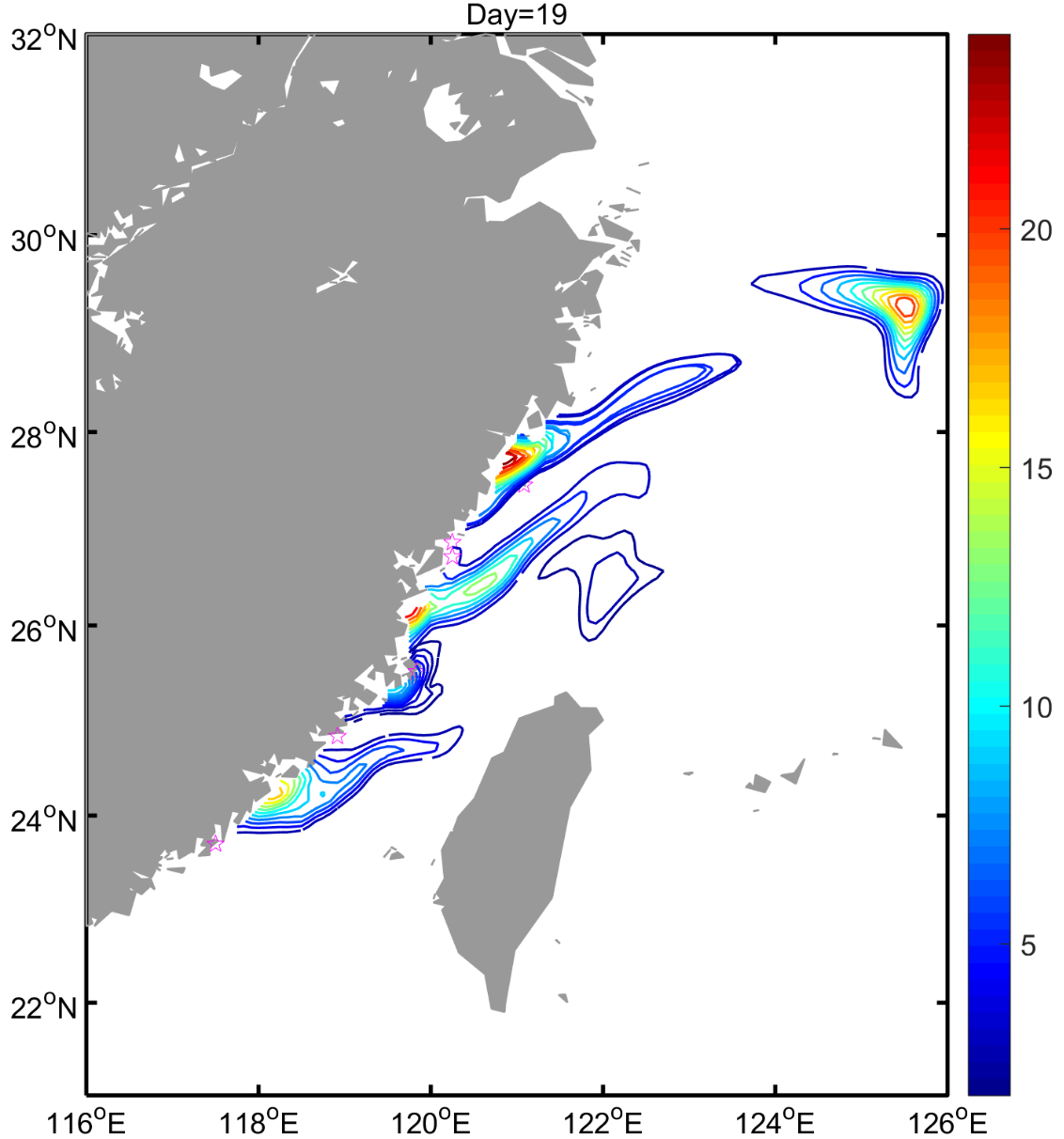

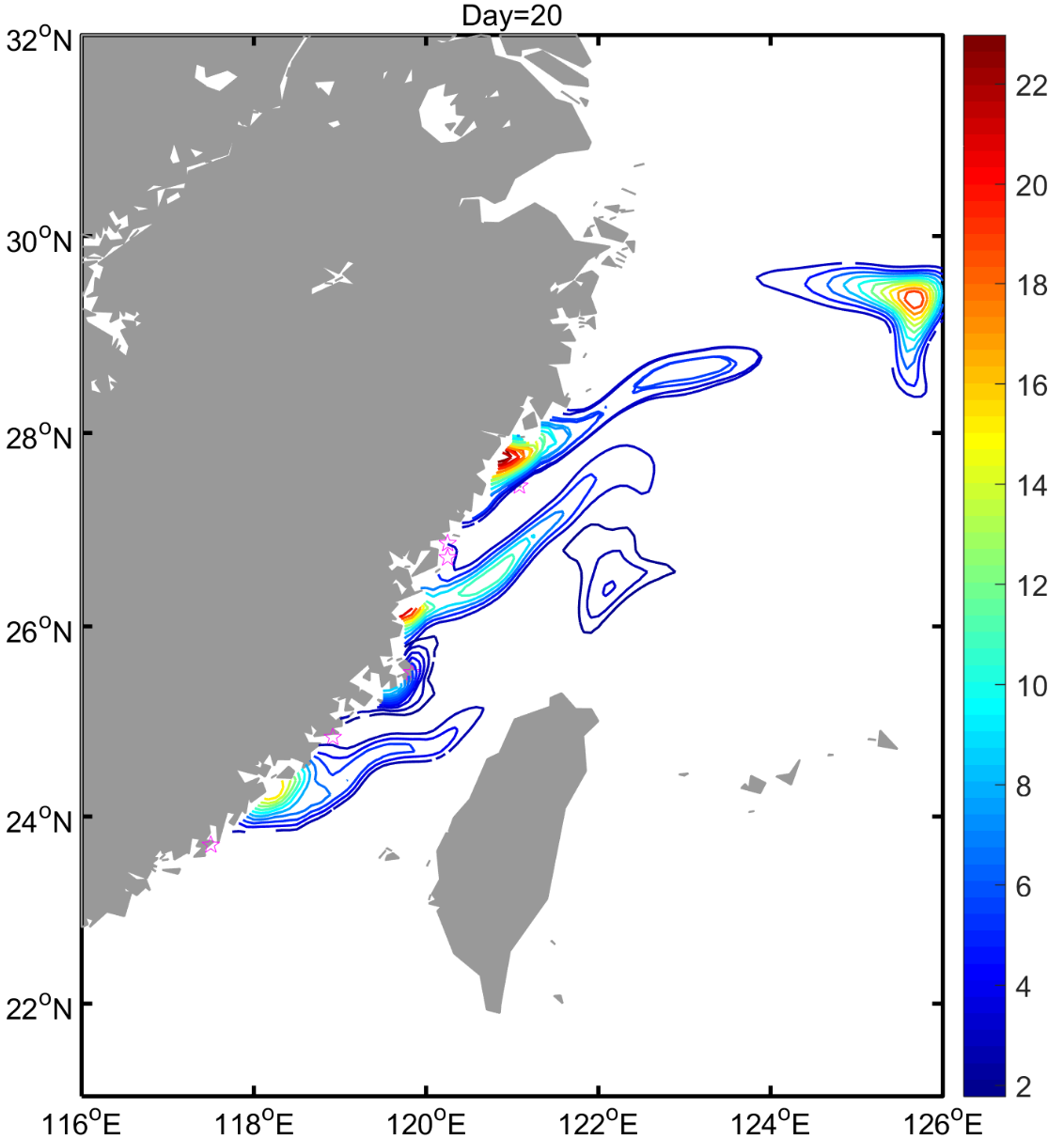

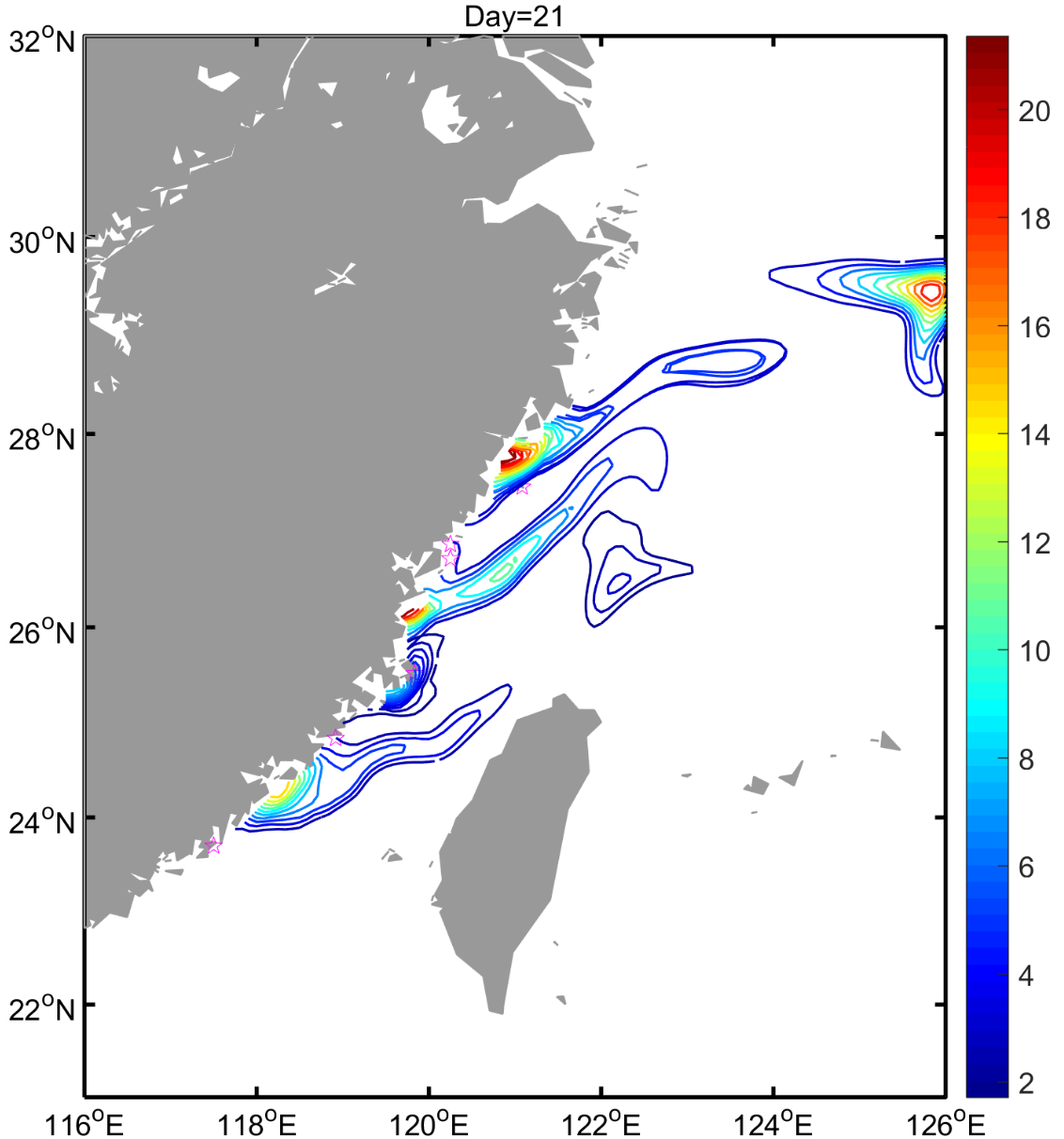

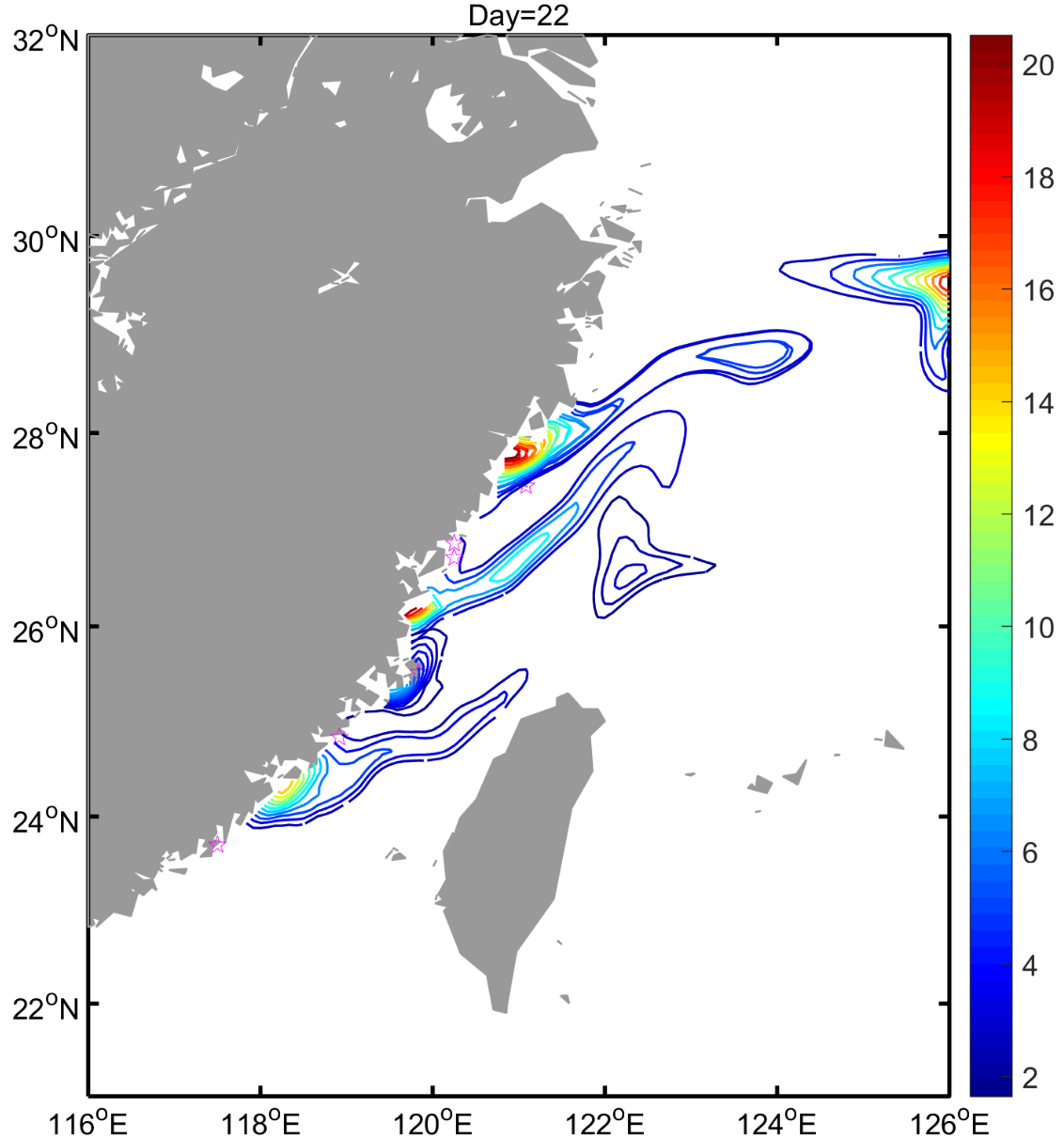

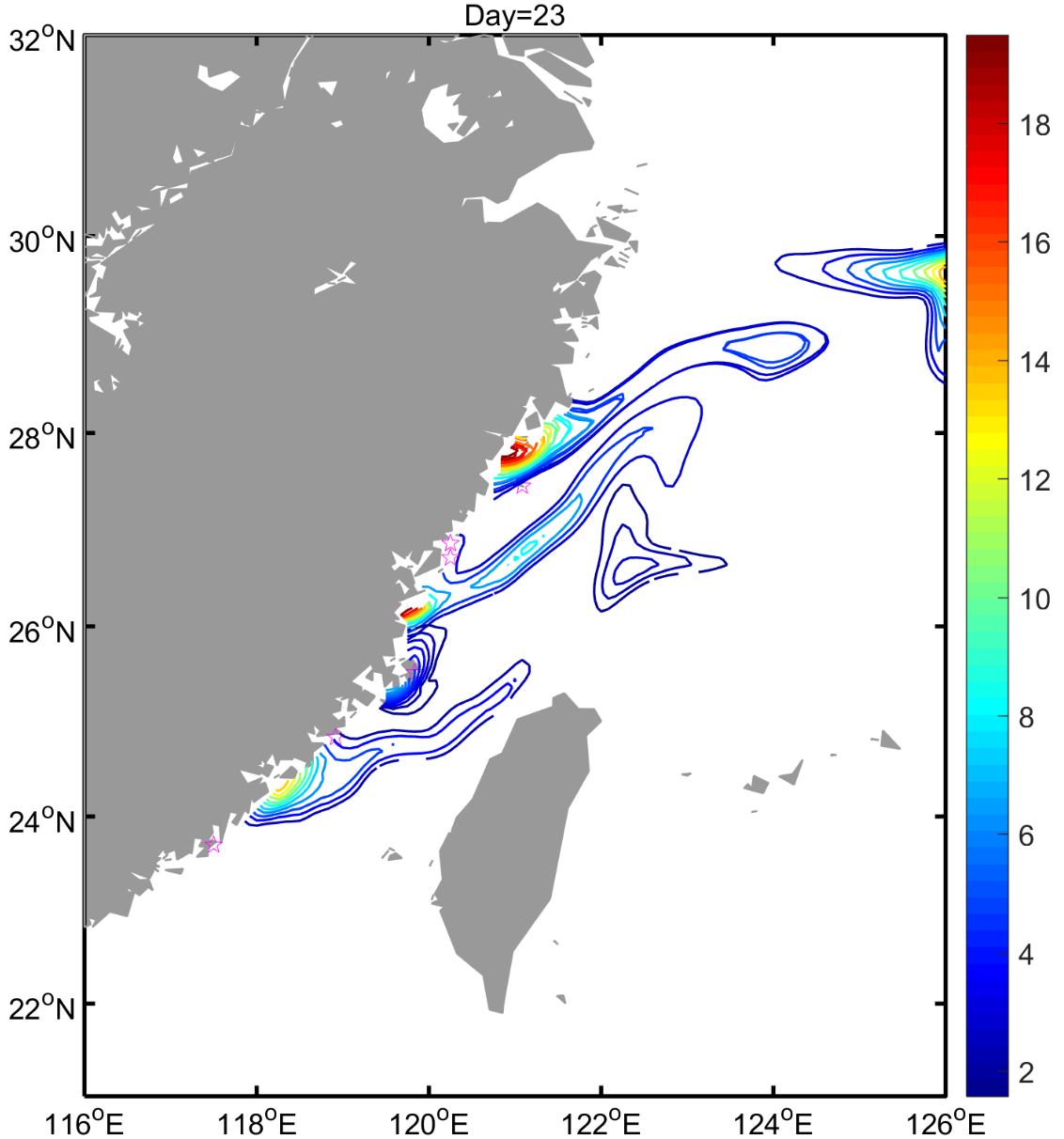

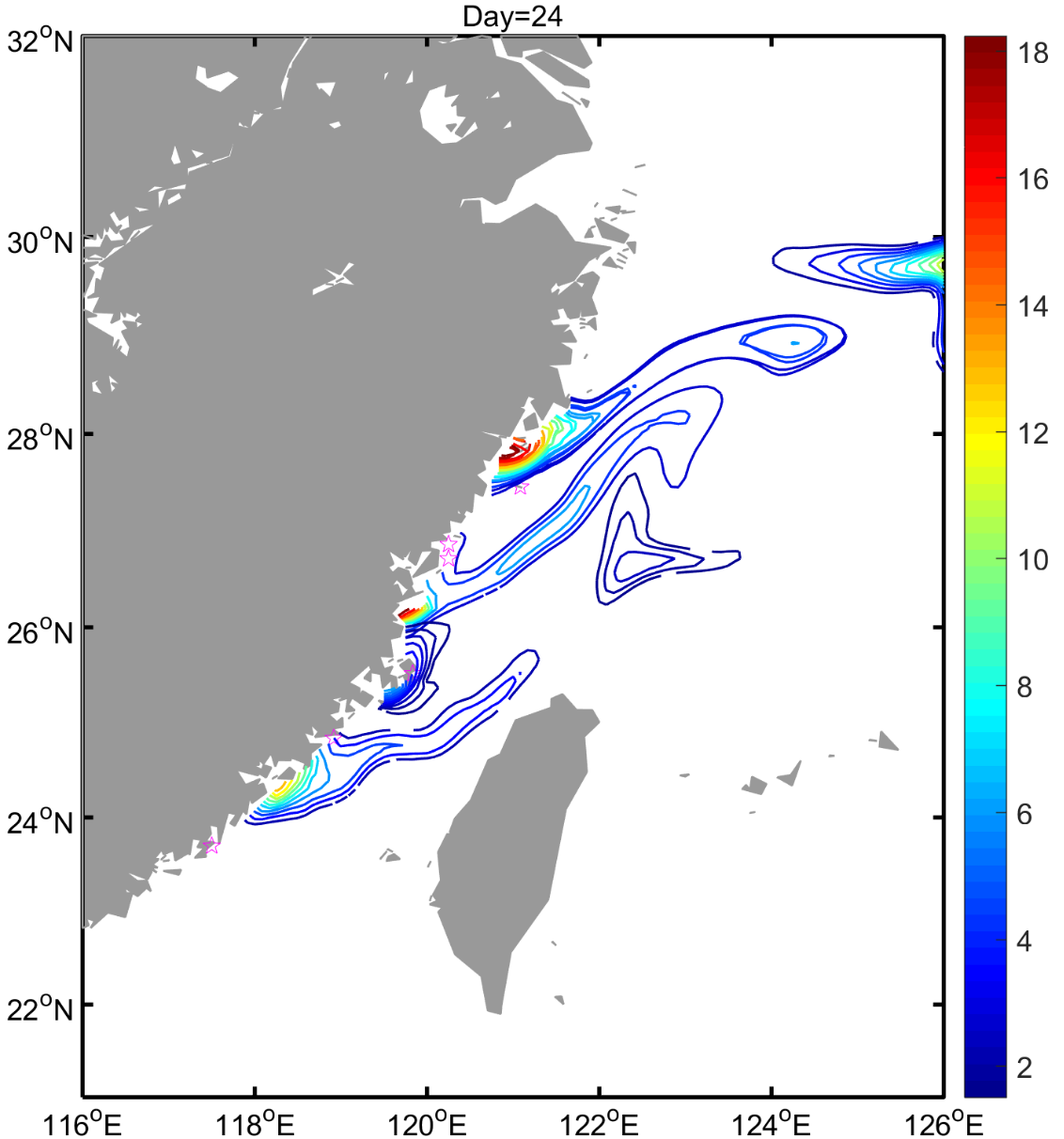

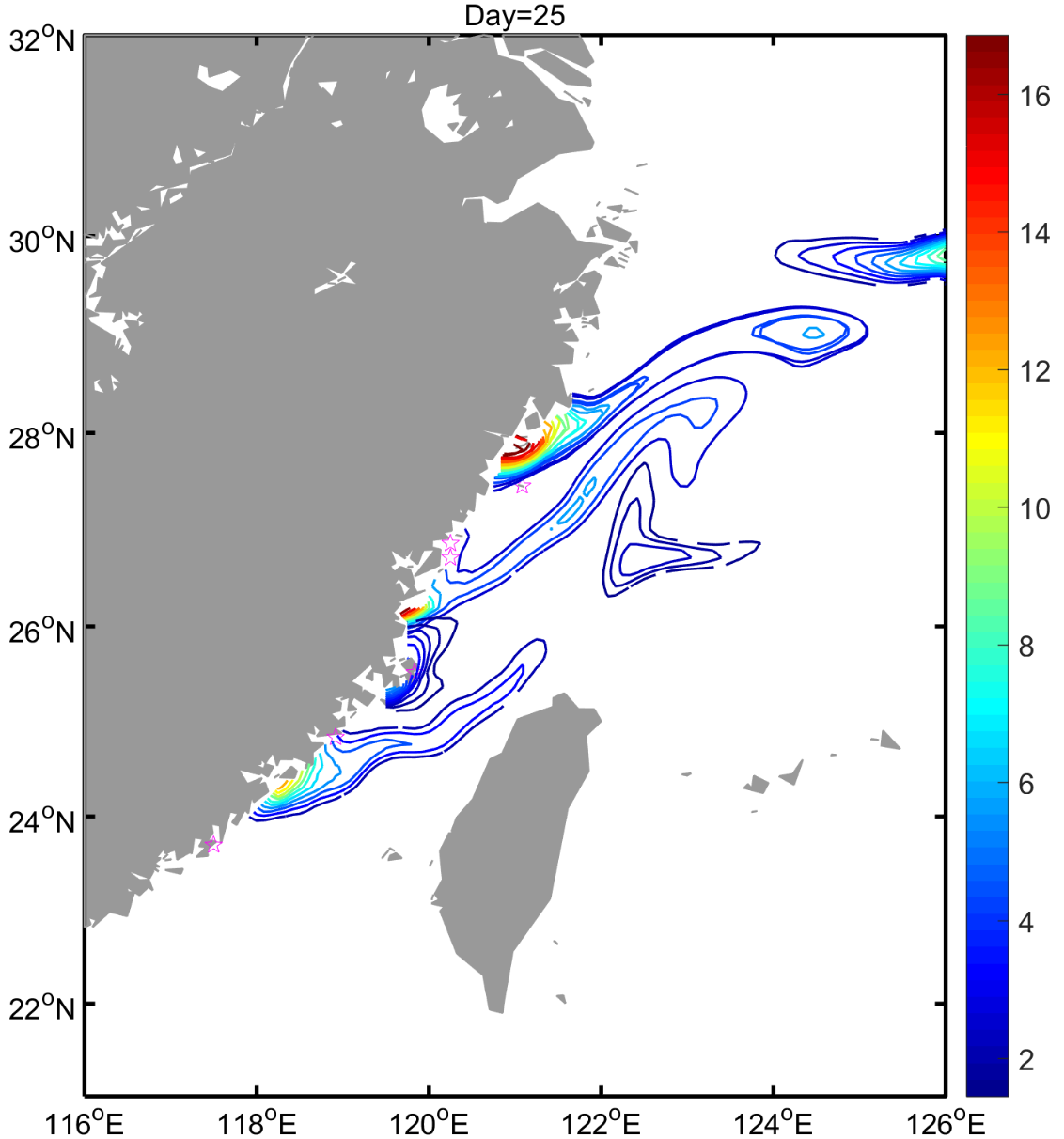

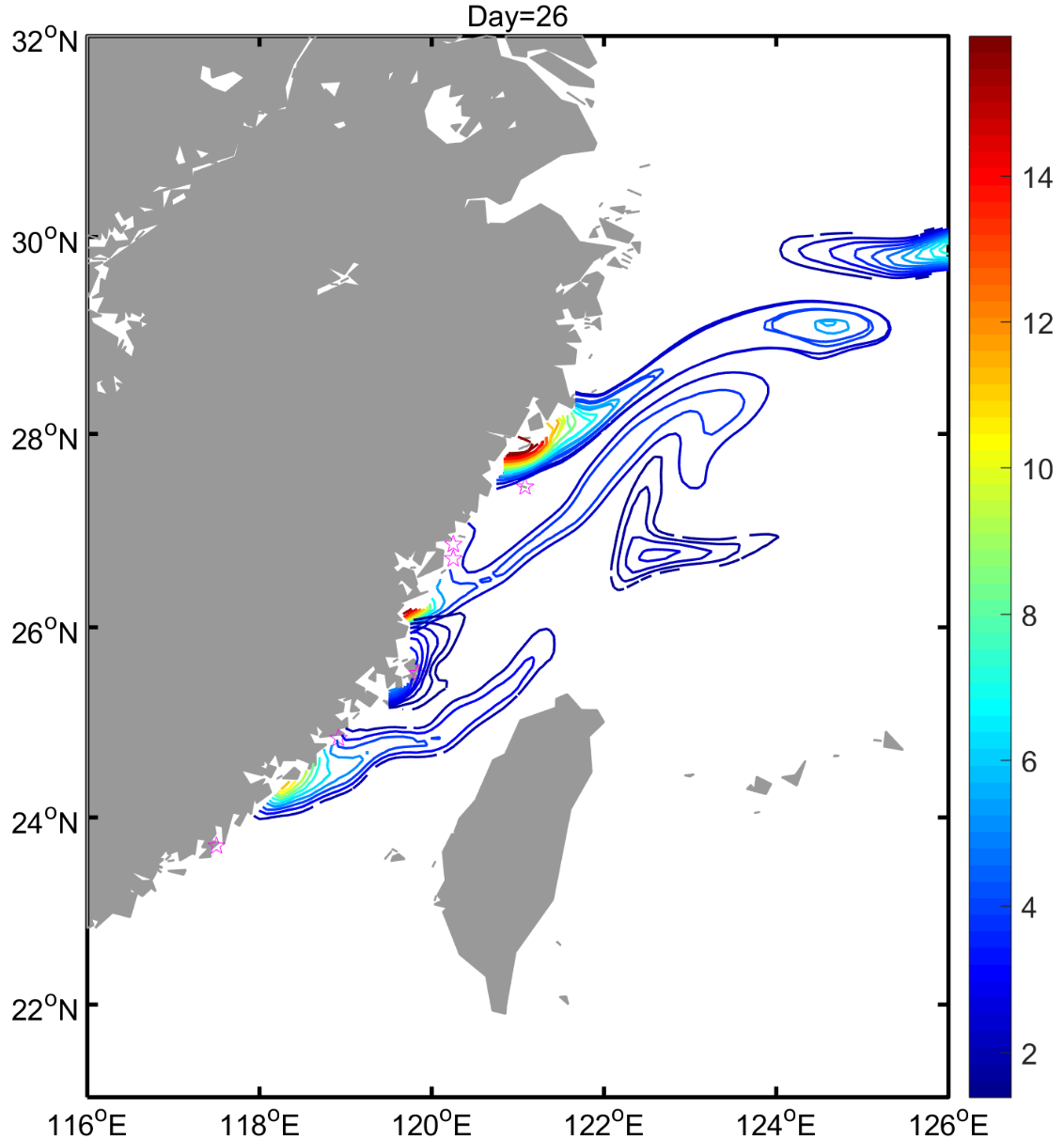

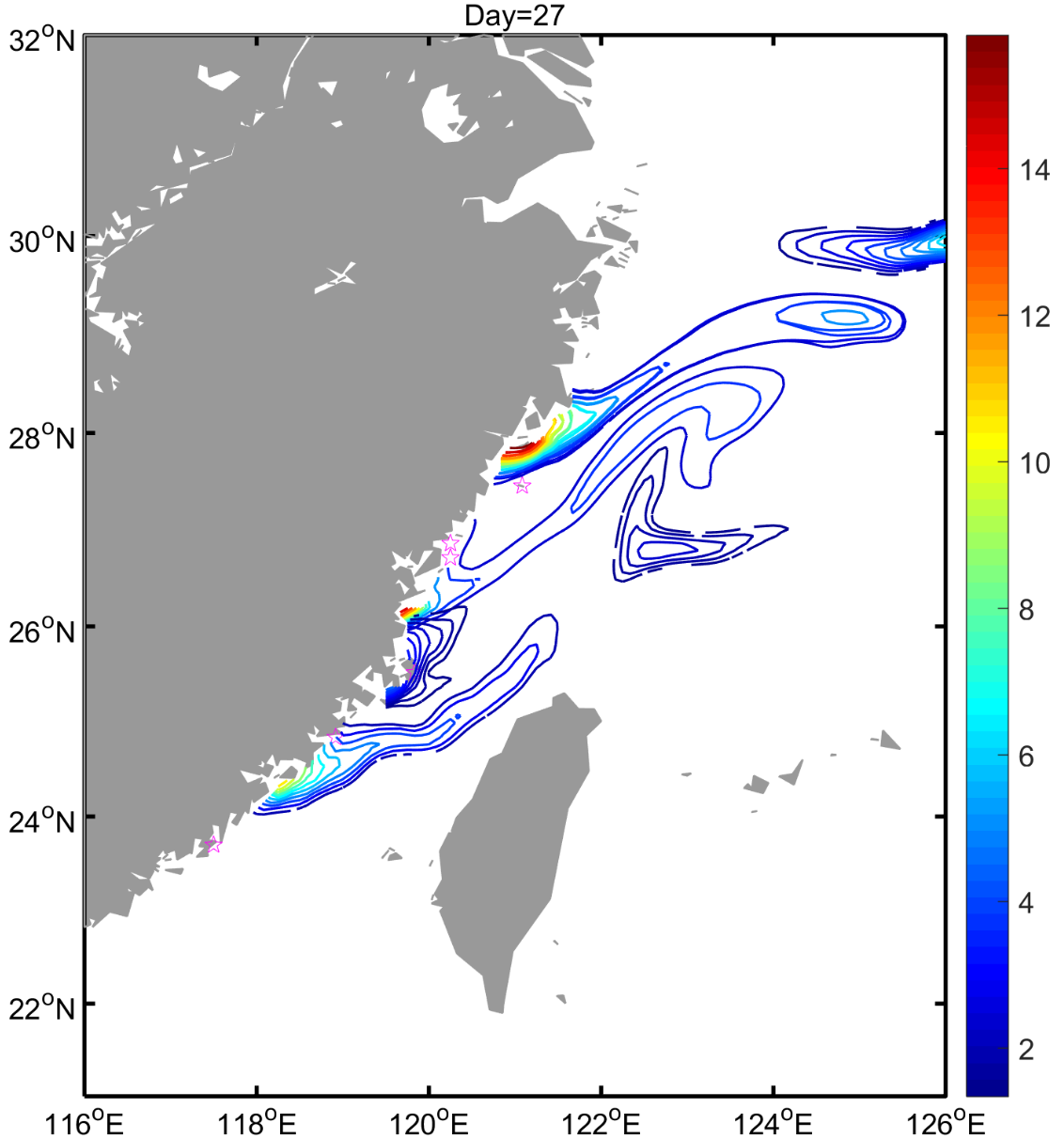

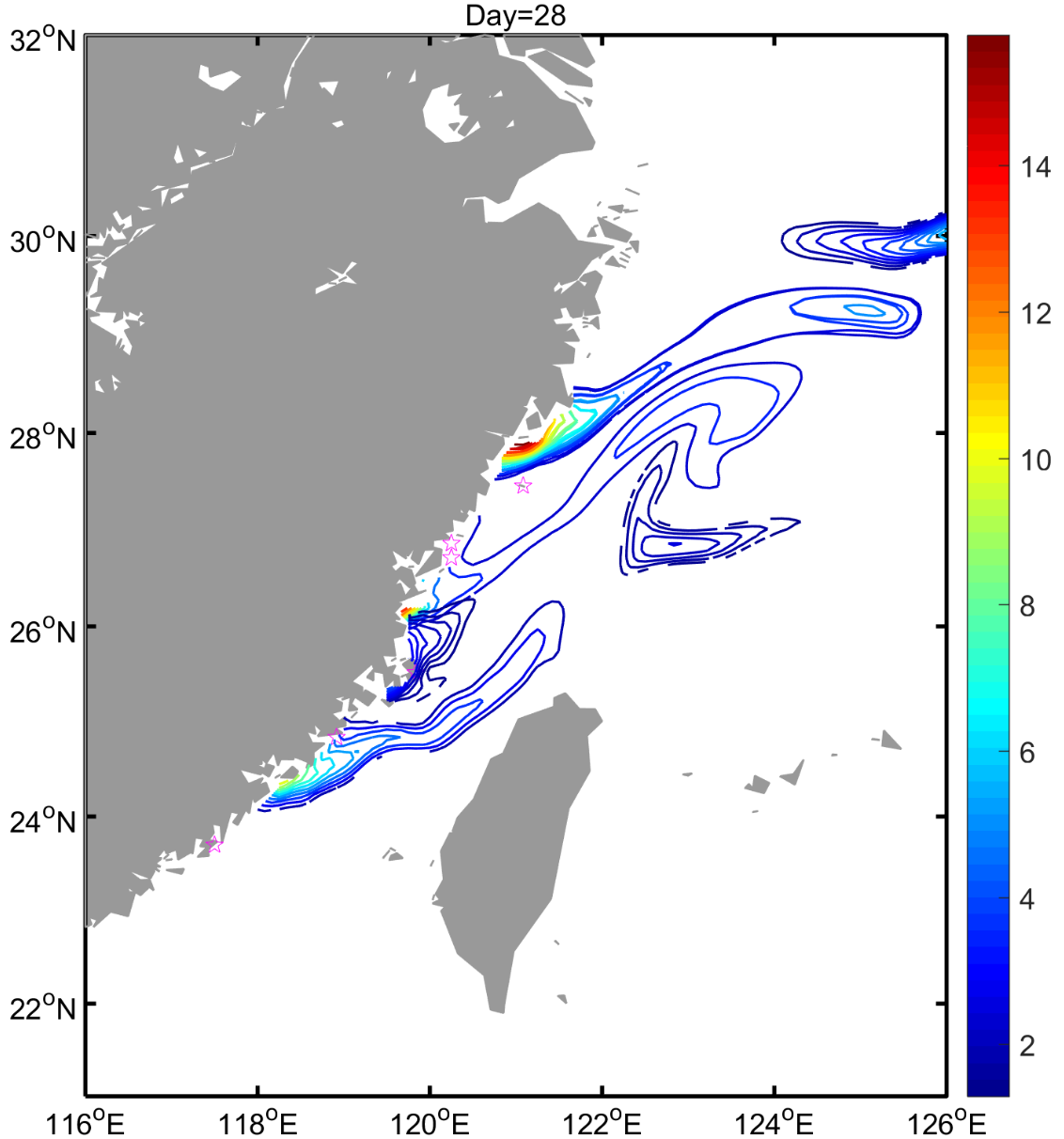

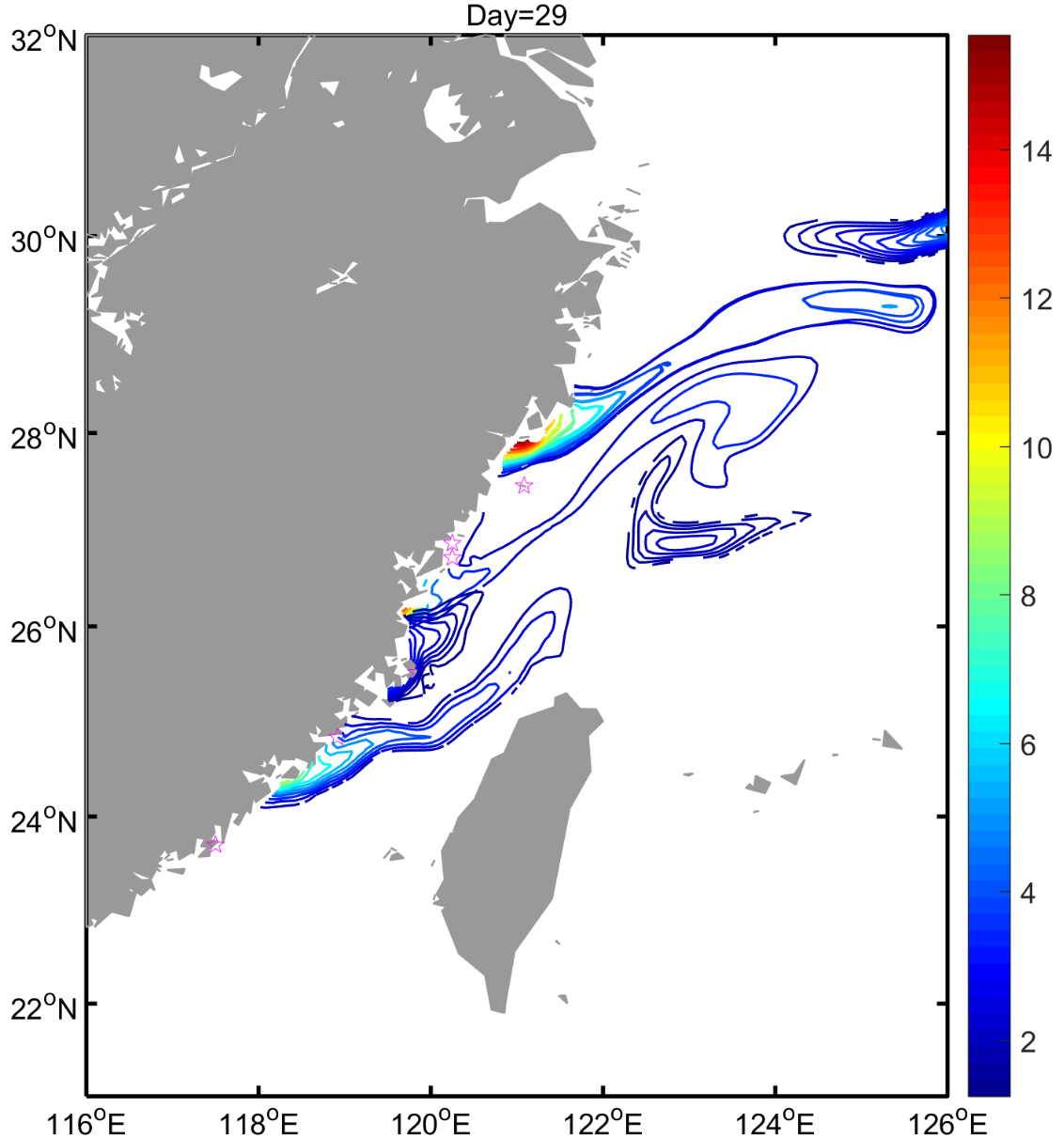

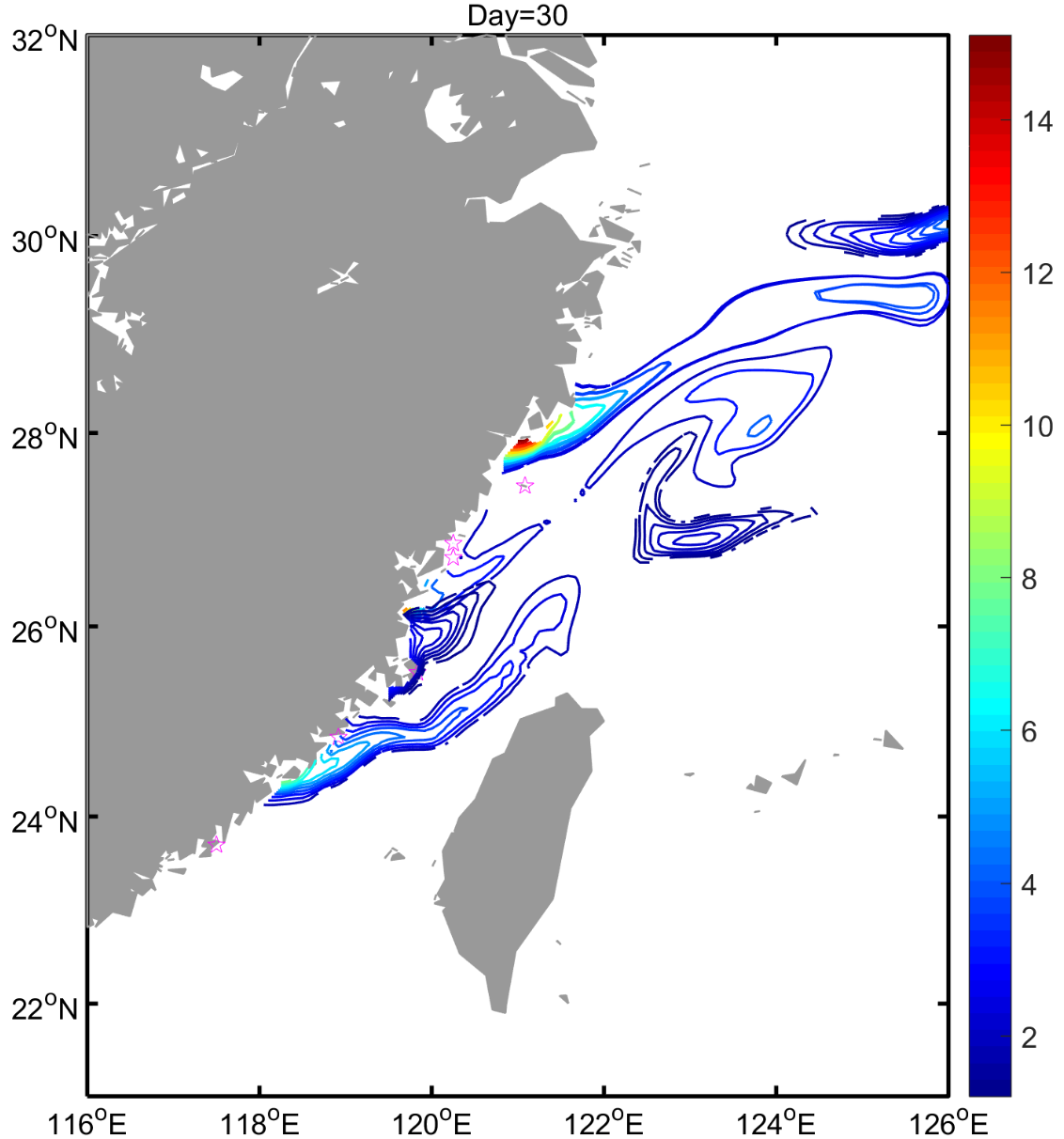


## **Figure S10** Dispersal path and relative content of particles originating from seven distinct localities within the Southern populations were modeled using the OGCM for one month.

## Table S1 Site locations, date of collection and sample size for all samples included in this study.

| Sites | Code | Sample size | Sample date | Longitude | Latitude |
| --- | --- | --- | --- | --- | --- |
| Dalian | DL | 20 | 2019.11.5 | 121°33′19″E | 38°52′9″N |
| Laizhou | LZ | 20 | 2019.10.10 | 119°35′19″°E | 37°15′9″N |
| Penglai | PL | 20 | 2021.4.27 | 120°44'48"°E | 37°49'41"N |
| Rongcheng | RC | 20 | 2019.11.1 | 122°35′57″E | 37°11′37″N |
| Qingdao | QD | 20 | 2019.10.9 | 120°20′39″E | 36°29′1″N |
| Rizhao | RZ | 20 | 2020.11.16 | 119°33'40"E | 35°23'40"N |
| Lianyungang | LYG | 20 | 2020.11.17 | 119°29'12"E | 34°45'39"N |
| Nanji | NJ | 20 | 2020.9.9 | 121°4'0"E | 27°26'25"N |
| Xiapu | XP | 20 | 2021.5.13 | 120°13′19″E | 26°51′11″N |
| Pingtan | PT | 20 | 2021.5.12 | 119°51′17″E | 25°28′59″N |
| Lianjiang | LJ | 20 | 2021.5.14 | 120°6′6″E | 26°44′43″N |
| Quanzhou | QZ | 20 | 2021.5.11 | 118°53′39″E | 24°53′28″N |
| Dongshan | DS | 20 | 2021.5.10 | 117°30′5″E | 23°43′25″N |

## Table S2 Prior distributions of demographic and historical parameters were used as summary statistics for simulations of population size change in DIYABC (Figure S1). Times of changes in effective population size are defined from the present (0) back in time: t1, t2. All time events are expressed in numbers of generations. Conditions: N1 < N, N2 < N, N3 < N, N4 < N, t3 > t1, t3 > t2.

| Parameter | Prior distribution |
| --- | --- |
| N1 | Uniform (10-10,000) |
| N2 | Uniform (10-10,000) |
| N3 | Uniform (10-10,000) |
| N4 | Uniform (10-10,000) |
| N | Uniform (10-10,000) |
| t1 | Uniform (10-10,000) |
| t2 | Uniform (10-10,000) |
| t3 | Uniform (10-1,000,000) |

In the scenario, t# represents the time-scale in terms of the number of generations, and N represents the effective population size of an unknown ancestral population. N1: effective population size (*N*_e_) of Dalian; N2: *N*_e_ of Lianyungang; N3: *N*_e_ of Nanji; N4: *N*_e_ of Dongshan. The common ancestral population of the Northern and Southern groups diverged at time t3 from an ancestral population of size N. Then the Northern group populations were simultaneously derived from the common ancestral population at time t2. The Southern group populations were simultaneously derived from the common ancestral population at time t1.

## Table S3 Summary of posterior probabilities of four distinct demographic history scenarios assessed through DIYABC analysis utilizing 1000 SNPs data.

| n | scenario 1 | scenario 2 | scenario 3 | scenario 4 |
| --- | --- | --- | --- | --- |
| 40000 | 0  [0.0000,1.0000] | 0  [0.0000,1.0000] | 0.004  [0.0000,1.0000] | 0.996  [0.9716,1.0000] |

n: number of data utilized; values in each bracket signify the 95% credibility interval values for each model. Posterior probabilities for all four scenarios are estimated using a subset of 1% of the simulated data sets. Scenario 4 is the most strongly supported scenario due to the highest probabilities.

**Table S4** Posterior distributions of population demographic parameters from the scenario with the highest posterior probability (Scenario 4) inferred by DIYABC analysis utilizing 1000 SNPs data.

| Parameter | mean | median | Mode | q025 | q050 | q250 | q750 | q950 | q975 |
| --- | --- | --- | --- | --- | --- | --- | --- | --- | --- |
| N1 | 9930 | 9950 | 9960 | 9790 | 9830 | 9920 | 9970 | 9990 | 9990 |
| N2 | 4390 | 4240 | 4120 | 1540 | 1880 | 3190 | 5360 | 7630 | 8530 |
| N3 | 481 | 321 | 223 | 87 | 112 | 210 | 490 | 1250 | 2150 |
| N4 | 9700 | 9760 | 9800 | 9080 | 9290 | 9630 | 9840 | 9950 | 9970 |
| N5 | 2230 | 1890 | 1570 | 555 | 701 | 1290 | 2700 | 4970 | 6440 |
| N6 | 9940 | 9950 | 9960 | 9810 | 9850 | 9930 | 9970 | 9990 | 9990 |
| N7 | 6580 | 6680 | 6890 | 3390 | 3930 | 5630 | 7590 | 8990 | 9380 |
| N8 | 4620 | 4470 | 4490 | 1700 | 2140 | 3470 | 5560 | 7730 | 8600 |
| N9 | 9270 | 9380 | 9470 | 7990 | 8360 | 9080 | 9600 | 9840 | 9910 |
| N10 | 556 | 400 | 310 | 117 | 146 | 269 | 597 | 1380 | 2140 |
| N12 | 8000 | 8180 | 8420 | 5330 | 5990 | 7460 | 8740 | 9480 | 9700 |
| N12 | 876 | 647 | 457 | 187 | 240 | 436 | 952 | 2260 | 3450 |
| N13 | 5460 | 5440 | 5240 | 2440 | 2910 | 4380 | 6460 | 8250 | 8930 |
| t1 | 682 | 662 | 677 | 370 | 417 | 550 | 787 | 1030 | 1120 |
| Na | 1980 | 1550 | 870 | 227 | 371 | 933 | 2520 | 5200 | 6670 |
| t2 | 1090 | 956 | 922 | 515 | 576 | 777 | 1180 | 1730 | 2130 |
| Nb | 10 | 10 | 10 | 10 | 10 | 10 | 10 | 10 | 10 |
| t3 | 578000 | 577000 | 584000 | 445000 | 469000 | 536000 | 617000 | 686000 | 720000 |
| N | 376 | 110 | 10 | 12 | 15 | 44 | 301 | 1660 | 2870 |

N: effective population size (*N*_e_) of an ancestral population, N1: *N*_e_ of Dalian, N2: *N*_e_ of Laizhou, N3: *N*_e_ of Penglai, N4: *N*_e_ of Rongcheng, N5: *N*_e_ of Qingdao, N6: *N*_e_ of Rizhao, N7: *N*_e_ of Lianyungang, N8: *N*_e_ of Nanji, N9: *N*_e_ of Xiapu, N10: *N*_e_ of Pingtan, N11: *N*_e_ of Lianjiang, N12: *N*_e_ of Quanzhou, N13: *N*_e_ of Dongshan, Na: *N*_e_ of common ancestral population of the Northern group, Nb: *N*_e_ of common ancestral population of the Southern group, t1: time of divergence between the Southern group and the common ancestral population (Nb) in generations; t2: time of divergence between the Northern group and the common ancestral population (Na) in generations; t3: time of divergence between the common ancestral population of the Northern (Na) and Southern groups (Nb) and ancestral population (N) in generations.

**Table S5** Summary of the top 20 ranked ‘simple’ model comparisons with model structure set to one time interval before the population split and one time interval after the split (1,1). Models are ordered by rank (1 to 20) according to Log-likelihood. LYG vs. NJ and DL vs. DS are shown in the top and bottom of the table, respectively.

| **LYG (pop1) vs. NJ (pop2)** | | | | | | | | | | | |
| --- | --- | --- | --- | --- | --- | --- | --- | --- | --- | --- | --- |
| **Rank** | **Log Likelihood** | **AIC** | **N ancestral** | **t1** | **nu11** | **nu12** | **m1_12** | **m1_21** | **contraction type pop1** | **contraction type pop2** | **theta** |
| 1 | -160.33 | 346.66 | 20901 | 59193.439 | 3288.772 | 3883.216 | 0.00E+00 | 0.00E+00 | Exp | Lin | 108.69 |
| 2 | -160.34 | 346.68 | 10036 | 116172.413 | 5738.062 | 4777.946 | 4.98E-08 | 0.00E+00 | Exp | Lin | 52.19 |
| 3 | -160.37 | 346.74 | 4046 | 38444.105 | 4046.54 | 196.677 | 0.00E+00 | 0.00E+00 | Lin | Lin | 21.04 |
| 4 | -160.48 | 346.95 | 12298 | 88401.261 | 12298.58 | 14994.67 | 0.00E+00 | 0.00E+00 | Lin | Sud | 63.95 |
| 5 | -199.69 | 425.38 | 41273 | 145747.584 | 7482.008 | 1015.404 | 1.07E-06 | 1.45E-07 | Exp | Lin | 214.62 |
| 6 | -199.96 | 425.93 | 43367 | 210854.042 | 5780.987 | 2346.286 | 1.38E-06 | 1.15E-08 | Lin | Lin | 225.51 |
| 7 | -200.47 | 426.93 | 58326 | 206377.604 | 5155.305 | 1982.252 | 1.15E-06 | 4.71E-10 | Lin | Lin | 303.3 |
| 8 | -200.57 | 427.13 | 54845 | 196485.975 | 7005.95 | 2475.127 | 1.09E-06 | 3.49E-08 | Exp | Lin | 285.2 |
| 9 | -201.37 | 428.74 | 31688 | 210160.031 | 5268.44 | 2345.964 | 8.61E-07 | 3.74E-07 | Exp | Lin | 164.78 |
| 10 | -202.31 | 430.62 | 51659 | 112205.974 | 7718.848 | 2046.15 | 9.16E-07 | 9.04E-09 | Exp | Lin | 268.63 |
| 11 | -202.86 | 431.71 | 19735 | 211915.14 | 8948.299 | 2909.334 | 1.28E-06 | 2.53E-08 | Sud | Lin | 102.63 |
| 12 | -203.00 | 431.99 | 78903 | 75370.34 | 8594.891 | 1803.968 | 6.64E-07 | 0.00E+00 | Sud | Lin | 410.3 |
| 13 | -203.49 | 432.98 | 55537 | 114633.007 | 6418.232 | 2190.884 | 9.38E-07 | 3.48E-08 | Lin | Lin | 288.79 |
| 14 | -204.56 | 435.12 | 71250 | 153380.968 | 4950.114 | 3311.643 | 8.95E-07 | 6.80E-08 | Lin | Lin | 370.51 |
| 15 | -204.99 | 435.99 | 60942 | 125844.942 | 7174.087 | 3774.932 | 8.47E-07 | 7.25E-09 | Exp | Lin | 316.9 |
| 16 | -205.05 | 436.09 | 36600 | 151339.863 | 8577.917 | 3783.568 | 1.20E-06 | 1.37E-08 | Sud | Lin | 190.32 |
| 17 | -205.49 | 436.99 | 18294 | 150213.122 | 4047.258 | 6695.832 | 1.03E-06 | 2.73E-08 | Lin | Lin | 95.13 |
| 18 | -205.64 | 437.29 | 96395 | 113551.29 | 4986.293 | 3912.587 | 7.61E-07 | 0.00E+00 | Lin | Lin | 501.26 |
| 19 | -205.74 | 437.48 | 73713 | 138736.818 | 5734.308 | 3645.454 | 7.25E-07 | 2.65E-11 | Exp | Lin | 383.31 |
| 20 | -205.87 | 437.74 | 35557 | 137435.87 | 5483.551 | 898.947 | 1.41E-08 | 1.17E-06 | Lin | Lin | 184.9 |
| **DL (pop1) vs. DS (pop2)** | | | | | | | | | | | |
| **Rank** | **Log Likelihood** | **AIC** | **N ancestral** | **t1** | **nu11** | **nu12** | **m1_12** | **m1_21** | **contraction type pop1** | **contraction type pop2** | **theta** |
| 1 | -214.10 | 446.20 | 21968 | 415149.124 | 10110.74 | 2983.467 | 9.71E-07 | 1.97E-07 | Lin | Lin | 114.24 |
| 2 | -214.10 | 446.21 | 20961 | 419196.743 | 10137.89 | 3081.233 | 9.92E-07 | 1.99E-07 | Lin | Lin | 109 |
| 3 | -214.13 | 446.27 | 28294 | 388333.725 | 10055.57 | 2951.086 | 9.70E-07 | 1.76E-07 | Lin | Lin | 147.13 |
| 4 | -214.16 | 446.32 | 32404 | 372229.069 | 10040.57 | 2981.079 | 9.63E-07 | 1.85E-07 | Lin | Lin | 168.5 |
| 5 | -214.20 | 446.40 | 36920 | 353513.685 | 10027.36 | 2969.565 | 9.71E-07 | 1.77E-07 | Lin | Lin | 191.99 |
| 6 | -214.20 | 446.40 | 36958 | 353431.708 | 10028.32 | 2968.215 | 9.57E-07 | 1.77E-07 | Lin | Lin | 192.18 |
| 7 | -214.20 | 446.40 | 36987 | 353318.809 | 10026.73 | 2968.971 | 9.57E-07 | 1.77E-07 | Lin | Lin | 192.33 |
| 8 | -214.23 | 446.46 | 39415 | 342242.599 | 10023.1 | 2937.156 | 9.55E-07 | 1.68E-07 | Lin | Lin | 204.96 |
| 9 | -214.48 | 446.96 | 30187 | 379780.829 | 10625.95 | 2945.488 | 9.66E-07 | 1.73E-07 | Exp | Lin | 156.97 |
| 10 | -214.57 | 447.15 | 20510 | 410218.637 | 11205.65 | 1944.341 | 1.05E-06 | 0.00E+00 | Lin | Lin | 106.66 |
| 11 | -214.60 | 447.19 | 43405 | 324960.582 | 10535.37 | 2912.642 | 9.42E-07 | 1.47E-07 | Exp | Lin | 225.71 |
| 12 | -214.63 | 447.26 | 45910 | 314671.413 | 10504.84 | 2910.215 | 9.39E-07 | 1.40E-07 | Exp | Lin | 238.73 |
| 13 | -214.63 | 447.26 | 45910 | 314523.616 | 10506.51 | 2911.25 | 9.50E-07 | 1.40E-07 | Exp | Lin | 238.73 |
| 14 | -214.67 | 447.35 | 48413 | 303334.331 | 10493.77 | 2903.674 | 9.36E-07 | 1.32E-07 | Exp | Lin | 251.75 |
| 15 | -215.38 | 448.75 | 73105 | 198194.034 | 10097.82 | 2911.497 | 8.39E-07 | 8.42E-11 | Lin | Lin | 380.15 |
| 16 | -215.46 | 448.92 | 71564 | 204927.812 | 10473.94 | 2860.033 | 8.50E-07 | 3.38E-12 | Exp | Lin | 372.14 |
| 17 | -215.69 | 449.38 | 74814 | 191071.335 | 10495.14 | 2944.654 | 8.36E-07 | 2.30E-09 | Exp | Lin | 389.04 |
| 18 | -215.69 | 449.39 | 74882 | 190784.13 | 10546.76 | 2959.677 | 8.30E-07 | 2.20E-11 | Exp | Lin | 389.39 |
| 19 | -215.84 | 449.69 | 66044 | 237606.838 | 8928.988 | 2879.098 | 1.08E-06 | 2.63E-09 | Lin | Lin | 343.43 |
| 20 | -216.09 | 450.17 | 77402 | 184032.725 | 8985.992 | 2921.625 | 8.86E-07 | 7.73E-12 | Lin | Lin | 402.49 |

AIC: Akaike information criterion; N ancestral: estimated ancestral population size before the split; t1: estimated time since divergence from the ancestral population; nu11: estimated contemporary effective population size of pop1; nu12: estimated contemporary effective population size of pop2; m1_12: estimated relative migration rate from pop2 to pop1; m1_21: estimated relative migration rate from pop1 to pop2. Lin: linear contraction; Exp: exponential contraction; Sud: sudden contraction.

**Table S6** Summary of the top 20 ranked ‘complex’ model comparisons with model structure set to one time interval before the population split and two time intervals after the split (1,2). Models are ordered by rank (1 to 20) according to Log-likelihood. LYG vs. NJ and DL vs. DS are shown in the top and bottom of the table, respectively.

| **LYG (pop1) vs. NJ (pop2)** | | | | | | | | | | | | | | |
| --- | --- | --- | --- | --- | --- | --- | --- | --- | --- | --- | --- | --- | --- | --- |
| **Rank** | **Log Likelihood** | **AIC** | **N ancestral** | **t1** | **nu11** | **nu12** | **m1_12** | **m1_21** | **t2** | **nu21** | **nu22** | **m2_12** | **m2_21** | **theta** |
| 1 | -160.32 | 368.65 | 11430 | 64263.641 | 15992.12 | 24108.478 | 0.00E+00 | 5.74E-08 | 32400.505 | 7632.618 | 9329.259 | 3.59E-24 | 7.33E-08 | 59.44 |
| 2 | -160.33 | 368.65 | 9728 | 21812.019 | 24924.266 | 11583.263 | 0.00E+00 | 0.00E+00 | 24217.842 | 8791.308 | 8705.738 | 0.00E+00 | 0.00E+00 | 50.59 |
| 3 | -160.33 | 368.65 | 53676 | 42804.05 | 1148.798 | 36975.466 | 0.00E+00 | 0.00E+00 | 30609.474 | 4419.254 | 3487.53 | 6.18E-07 | 0.00E+00 | 279.12 |
| 4 | -160.33 | 368.66 | 18486 | 64263.641 | 14644.731 | 61990.579 | 0.00E+00 | 0.00E+00 | 29572.412 | 6098.499 | 13546.61 | 0.00E+00 | 0.00E+00 | 96.13 |
| 5 | -160.33 | 368.66 | 15284 | 36212.646 | 11566.257 | 28688.288 | 3.30E-08 | 0.00E+00 | 47624.86 | 4701.304 | 9343.987 | 1.68E-06 | 0.00E+00 | 79.48 |
| 6 | -160.33 | 368.66 | 17928 | 100746.529 | 17515.901 | 35842.754 | 0.00E+00 | 0.00E+00 | 81878.194 | 13356.63 | 8581.622 | 3.32E-16 | 3.21E-08 | 93.23 |
| 7 | -160.33 | 368.66 | 9339 | 29368.167 | 35866.102 | 19514.784 | 0.00E+00 | 0.00E+00 | 29386.846 | 5723.735 | 5928.337 | 0.00E+00 | 0.00E+00 | 48.56 |
| 8 | -160.34 | 346.67 | 10140 | 42185.045 | 3836.884 | 10192.563 | 0.00E+00 | 0.00E+00 |  |  |  |  |  | 52.73 |
| 9 | -160.39 | 364.77 | 2668 | 9966.219 | 6267.349 | 670.761 | 2.12E-05 | 6.81E-04 | 7069.962 | 298.274 | 62.048 | 0.00E+00 | 0.00E+00 | 13.87 |
| 10 | -160.41 | 368.82 | 28649 | 101123.56 | 28649.817 | 28649.817 | 4.59E-05 | 0.00E+00 | 88650.763 | 15719.15 | 9390.093 | 0.00E+00 | 0.00E+00 | 148.98 |
| 11 | -160.45 | 368.90 | 40524 | 439268.786 | 114996.4 | 213415.044 | 2.51E-05 | 5.62E-06 | 169008.43 | 6714.39 | 13284.27 | 0.00E+00 | 4.47E-08 | 210.73 |
| 12 | -180.31 | 408.62 | 5350 | 103807.923 | 5349.785 | 659.301 | 5.97E-05 | 1.19E-04 | 9776.604 | 2630.73 | 494.87 | 9.34E-08 | 4.07E-06 | 27.82 |
| 13 | -186.79 | 421.57 | 34012 | 147652.98 | 34012.888 | 56753.521 | 1.47E-08 | 0.00E+00 | 25086.132 | 2560.487 | 529.337 | 2.16E-06 | 1.72E-06 | 176.87 |
| 14 | -192.41 | 432.83 | 3543 | 54313.14 | 7847.493 | 930.676 | 1.48E-18 | 5.22E-05 | 24799.943 | 7306.011 | 3544.337 | 1.41E-07 | 1.60E-06 | 18.43 |
| 15 | -195.25 | 438.51 | 69289 | 70244.648 | 26223.76 | 61990.968 | 0.00E+00 | 0.00E+00 | 55633.382 | 5656.926 | 1754.913 | 1.33E-06 | 4.24E-08 | 360.31 |
| 16 | -195.53 | 439.06 | 56378 | 57331.787 | 11547.213 | 98716.915 | 1.10E-06 | 0.00E+00 | 52783.294 | 6798.453 | 563.78 | 1.27E-06 | 3.63E-07 | 293.17 |
| 17 | -195.68 | 439.35 | 40995 | 95856.088 | 22472.431 | 155300.903 | 2.75E-07 | 0.00E+00 | 95169.697 | 5839.626 | 409.958 | 1.32E-06 | 1.22E-08 | 213.18 |
| 18 | -196.96 | 441.92 | 25325 | 64462.117 | 30322.094 | 70648.884 | 1.51E-08 | 1.34E-09 | 51733.945 | 7505.902 | 1287.184 | 1.02E-06 | 7.89E-07 | 131.69 |
| 19 | -197.00 | 441.99 | 63930 | 50017.911 | 9950.99 | 3794.904 | 5.92E-08 | 0.00E+00 | 35445.559 | 6629.723 | 925.287 | 8.38E-07 | 0.00E+00 | 332.44 |
| 20 | -197.15 | 442.31 | 46202 | 55755.307 | 12975.532 | 87442.313 | 0.00E+00 | 0.00E+00 | 56380.571 | 7024.89 | 883.461 | 8.21E-07 | 1.08E-08 | 240.26 |
| **DL (pop1) vs. DS (pop2)** | | | | | | | | | | | | | | |
| **Rank** | **Log Likelihood** | **AIC** | **N ancestral** | **t1** | **nu11** | **nu12** | **m1_12** | **m1_21** | **t2** | **nu21** | **nu22** | **m2_12** | **m2_21** | **theta** |
| 1 | -186.08 | 404.17 | 19440 | 388808.543 | 38441.95 | 82517.001 | 0.00E+00 | 0.00E+00 | 11965.908 | 979.023 | 1671.138 | 2.69E-06 | 3.18E-06 | 101.09 |
| 2 | -186.56 | 405.11 | 32698 | 330505.361 | 48191.658 | 54735.857 | 0.00E+00 | 0.00E+00 | 18691.785 | 1352.263 | 326.981 | 2.13E-06 | 2.28E-06 | 170.03 |
| 3 | -187.98 | 407.96 | 61640 | 214468.566 | 57016.179 | 58475.695 | 0.00E+00 | 0.00E+00 | 22786.53 | 1397.006 | 616.409 | 1.97E-06 | 1.84E-06 | 320.53 |
| 4 | -188.96 | 409.93 | 18957 | 379153.896 | 46437.487 | 60607.712 | 0.00E+00 | 0.00E+00 | 25908.791 | 2701.787 | 585.671 | 1.60E-06 | 1.81E-06 | 98.58 |
| 5 | -189.12 | 410.24 | 27462 | 359224.1 | 27489.532 | 53769.393 | 0.00E+00 | 0.00E+00 | 10530.425 | 1716.017 | 1933.187 | 2.51E-06 | 2.93E-06 | 142.80 |
| 6 | -189.13 | 410.26 | 32328 | 333462.94 | 32328.131 | 149954.514 | 3.67E-10 | 0.00E+00 | 18987.979 | 3122.911 | 2053.681 | 1.80E-06 | 2.58E-06 | 168.11 |
| 7 | -189.38 | 410.75 | 29892 | 342776.356 | 29892.25 | 53566.281 | 0.00E+00 | 0.00E+00 | 17479.401 | 3384.885 | 298.923 | 1.76E-06 | 2.45E-06 | 155.44 |
| 8 | -190.17 | 412.35 | 62526 | 221613.41 | 33742.421 | 27657.557 | 0.00E+00 | 0.00E+00 | 9376.041 | 625.262 | 25.262 | 3.21E-06 | 2.49E-06 | 325.14 |
| 9 | -191.54 | 415.08 | 24881 | 367490.536 | 24906.449 | 49569.261 | 0.00E+00 | 0.00E+00 | 18106.389 | 4352.95 | 455.352 | 1.64E-06 | 2.26e-06( | 129.38 |
| 10 | -192.25 | 416.50 | 32371 | 324383.216 | 32403.883 | 61321.687 | 0.00E+00 | 0.00E+00 | 29671.575 | 4954.641 | 749.827 | 1.40E-06 | 1.50E-06 | 168.33 |
| 11 | -192.53 | 417.05 | 37916 | 308489.288 | 36355.039 | 36412.68 | 0.00E+00 | 0.00E+00 | 20096.134 | 3246.352 | 1180.792 | 1.77E-06 | 1.43E-06 | 197.16 |
| 12 | -193.32 | 418.64 | 29886 | 331552.693 | 29916.836 | 103237.087 | 0.00E+00 | 0.00E+00 | 36883.521 | 6249.615 | 298.87 | 1.24E-06 | 1.73E-06 | 155.41 |
| 13 | -199.20 | 430.39 | 40241 | 267468.367 | 22696.208 | 154747.086 | 0.00E+00 | 0.00E+00 | 65171.623 | 8683.827 | 402.415 | 8.92E-07 | 1.35E-06 | 209.26 |
| 14 | -200.35 | 432.71 | 71095 | 183364.845 | 16164.66 | 71092.972 | 6.06E-07 | 0.00E+00 | 31674.311 | 8875.107 | 710.955 | 9.17E-07 | 1.35E-06 | 369.70 |
| 15 | -200.43 | 432.85 | 17539 | 350790.768 | 23549.097 | 124163.705 | 0.00E+00 | 0.00E+00 | 68612.758 | 8678.139 | 1079.809 | 8.94E-07 | 1.16E-06 | 91.21 |
| 16 | -201.16 | 434.32 | 89253 | 106067.432 | 14991.565 | 50923.006 | 0.00E+00 | 0.00E+00 | 21619.657 | 8288.186 | 892.534 | 1.01E-06 | 1.20E-06 | 464.12 |
| 17 | -208.30 | 448.60 | 32520 | 207436.935 | 26490.736 | 196351.234 | 1.12E-07 | 7.68E-11 | 163171.388 | 9973.631 | 1059.697 | 1.16E-06 | 2.26E-07 | 169.10 |
| 18 | -209.42 | 450.84 | 17772 | 355441.764 | 40485.794 | 110830.647 | 0.00E+00 | 0.00E+00 | 54452.488 | 4411.667 | 782.937 | 2.81E-08 | 2.01E-06 | 92.41 |
| 19 | -210.06 | 452.12 | 32086 | 228223.751 | 31059.704 | 118435.456 | 0.00E+00 | 0.00E+00 | 138144.329 | 9033.419 | 1802.575 | 1.15E+00 | 1.56E-08 | 166.85 |
| 20 | -212.18 | 456.36 | 43517 | 181752.427 | 24180.787 | 105244.267 | 0.00E+00 | 0.00E+00 | 138649.119 | 9550.63 | 2196.346 | 1.05E-06 | 1.15E-08 | 226.29 |

AIC: Akaike information criterion; N ancestral: estimated ancestral population size before the split; t1: estimated time since divergence during the first time interval; nu11: estimated contemporary effective population size of pop1 during the first time interval; nu12: estimated contemporary effective population size of pop2 during the first time interval; m1_12: estimated relative migration rate from pop2 to pop1 during the first time interval; m1_21: estimated relative migration rate from pop1 to pop2 during the first time interval. t2: estimated time since divergence during the second time interval; nu21: estimated contemporary effective population size of pop1 during the second time interval; nu22: estimated contemporary effective population size of pop2 during the second time interval; m2_12: estimated relative migration rate from pop2 to pop1 during the second time interval; m2_21: estimated relative migration rate from pop1 to pop2 during the first time interval.
