# Supplementary material for: The Intertidal North‐South Split: Oceanographic Features and Life History Shape the Phylogeography of Chiton Acanthochitona rubrolineata
Source: Evol Appl. 2025 Mar 31;18(4):e70095. doi: 10.1111/eva.70095 (PMC11955844; doi:10.1111/eva.70095)
Supplement: Supplementary file 2 — Movies S1. Dispersal path and relative content of particles originating from seven distinct localities within the Northern populations were modeled using the OGCM over 30 days. From top to bottom, the locations marked with pink pentagon stars are as follows: DL, Dalian; LYG, LianyungangLZ, Laizhou; PL, Penglai; QD, Qingdao; RC, Rongcheng; RZ, Rizhao. Movie S2. Dispersal path and relative content of particles originating from six distinct localities within the Southern populations were modeled using the OGCM over 30 days. From top to bottom, the locations marked with pink pentagon stars are as follows: DS, Dongshan; LJ, Lianjiang; NJ, Nanji; PT, Pingtan; QZ, Quanzhou; XP, Xiapu. [file EVA-18-e70095-s001.zip › eva70095-sup-0002-MoviesS1-S2.docx]

**Movie S1.** Dispersal path and relative content of particles originating from seven distinct localities within the Northern populations were modeled using the OGCM over 30 days. From top to bottom, the locations marked with pink pentagon stars are as follows: DL: Dalian, PL: Penglai, LZ: Laizhou, RC: Rongcheng, QD: Qingdao, RZ: Rizhao, LYG: Lianyungang.

**Movie S2.** Dispersal path and relative content of particles originating from six distinct localities within the Southern populations were modeled using the OGCM over 30 days. From top to bottom, the locations marked with pink pentagon stars are as follows: NJ: Nanji, XP: Xiapu, PT: Pingtan, LJ: Lianjiang, QZ: Quanzhou and DS: Dongshan.
